# Annelid methylomes reveal ancestral developmental and aging-associated epigenetic erosion across Bilateria

**DOI:** 10.1186/s13059-024-03346-z

**Published:** 2024-08-01

**Authors:** Kero Guynes, Luke A. Sarre, Allan M. Carrillo-Baltodano, Billie E. Davies, Lan Xu, Yan Liang, Francisco M. Martín-Zamora, Paul J. Hurd, Alex de Mendoza, José M. Martín-Durán

**Affiliations:** 1 School of Biological and Behavioural Sciences, Queen Mary University of London, Mile End Road, London, E1 4NS UK; 2 Institute of Molecular Biotechnology of the Austrian Academy of Sciences (IMBA), Vienna BioCenter (VBC), Vienna, 1030 Austria; 3 Altos Labs, Cambridge, UK

**Keywords:** DNA methylation, Whole-genome bisulfite sequencing, EM-seq, Annelida, Spiralia, Spiral cleavage, Transposable element, Methylome, Methylation clock

## Abstract

**Background:**

DNA methylation in the form of 5-methylcytosine (5mC) is the most abundant base modification in animals. However, 5mC levels vary widely across taxa. While vertebrate genomes are hypermethylated, in most invertebrates, 5mC concentrates on constantly and highly transcribed genes (gene body methylation; GbM) and, in some species, on transposable elements (TEs), a pattern known as “mosaic”. Yet, the role and developmental dynamics of 5mC and how these explain interspecies differences in DNA methylation patterns remain poorly understood, especially in Spiralia, a large clade of invertebrates comprising nearly half of the animal phyla.

**Results:**

Here, we generate base-resolution methylomes for three species with distinct genomic features and phylogenetic positions in Annelida, a major spiralian phylum. All possible 5mC patterns occur in annelids, from typical invertebrate intermediate levels in a mosaic distribution to hypermethylation and methylation loss. GbM is common to annelids with 5mC, and methylation differences across species are explained by taxon-specific transcriptional dynamics or the presence of intronic TEs. Notably, the link between GbM and transcription decays during development, alongside a gradual and global, age-dependent demethylation in adult stages. Additionally, reducing 5mC levels with cytidine analogs during early development impairs normal embryogenesis and reactivates TEs in the annelid *Owenia fusiformis*.

**Conclusions:**

Our study indicates that global epigenetic erosion during development and aging is an ancestral feature of bilateral animals. However, the tight link between transcription and gene body methylation is likely more important in early embryonic stages, and 5mC-mediated TE silencing probably emerged convergently across animal lineages.

**Supplementary Information:**

The online version contains supplementary material available at 10.1186/s13059-024-03346-z.

## Background

The reversible and heritable methylation of DNA, predominantly in the fifth position of the aromatic ring of a cytosine (i.e., 5-methylcytosine, or 5mC) of CpG dinucleotides, is a base modification that modulates diverse biological processes in animals and other eukaryotic lineages [1–3]. DNA methyltransferases (DNMTs) deposit this DNA modification, which is erased by ten-eleven translocation (TET) dioxygenases and bound by proteins containing methyl-CpG-binding domains (MBD) proteins [4–6]. The main DNMT families in animals include the maintenance type DNMT1 and the de novo DNMT3, whereas DNMT2 is a conserved tRNA methyltransferase [5]. Although this genetic toolkit to deposit, read, and erase 5mC is generally conserved in animals [7, 8], the genomic distribution of this base modification varies dramatically across the species in which it has been investigated [1, 2, 6]. Vertebrate genomes are hypermethylated since 5mC is widespread and occurs at high levels except at promoters and active distal regulatory elements [9]. In contrast, 5mC is sparsely distributed in most invertebrate genomes, exhibiting a mosaic pattern primarily concentrated in active gene bodies and sometimes transposable elements (TEs) [10–12]. However, some invertebrates have secondarily diverged from this condition and display vertebrate-like hypermethylated [7] or, more frequently, unmethylated genomes [13, 14], including the two best-established invertebrate systems, the fly *Drosophila melanogaster* and the nematode worm *Caenorhabditis elegans* [13, 14].

These contrasting genomic landscapes correlate with potentially distinct roles of 5mC in animal lineages. While DNA methylation is essential for normal embryogenesis in the hypermethylated vertebrate genomes [15–17], the function of this methyl mark is less well understood in invertebrates because most of the work has focussed on lineages with generally low methylation levels, such as insects [18, 19]. Nonetheless, 5mC has been proposed to contribute to gene regulation in invertebrates by repressing intragenic spurious transcription start sites [20], modulating gene transcription levels in response to environmental cues [21, 22], and controlling the activity of *cis*-regulatory elements [23]. However, the “epigenetic reprogramming” occurring in early embryogenesis in vertebrates has not been observed in invertebrates [24], where developmental 5mC levels are generally constant, with recent reports of late 5mC developmental dynamics in invertebrate deuterostomes [23, 25]. The evolution and ancestral role of DNA methylation in animal genomes thus remains poorly understood, mainly because we lack comprehensive functional studies that profile 5mC at base resolution during development and across phylogeny for most invertebrate lineages.

Spiralia (also known as Lophotrochozoa) is one of the three major clades of bilaterally symmetrical animals, including species with economic, ecological, and societal importance [26]. Despite comprising nearly half of all animal phyla [27], our knowledge of genome regulation in this animal clade is limited [28, 29], which, together with the more divergent genomic features of traditional invertebrate models (e.g., insects and nematode worms), ultimately impacts our capacity to reconstruct ancestral characters for crucial nodes of the animal tree of life, most notably the last common bilaterian ancestor. The study of DNA methylation has mainly focused on only four of the 15 spiralian groups, namely rotifers, platyhelminthes, molluscs, and annelids, often using in silico predictions and low-resolution profiling techniques. Rotifers have a divergent DNA methylation toolkit, lacking DNMTs and TET genes [30, 31]. Instead of 5mC, they rely on a horizontally acquired bacterial methyltransferase that modifies their cytosines in the fourth position of the pyrimidine ring (4mC) to regulate TE activity [32]. 5mC DNA methylation is also absent in some platyhelminthes, such as the planarian *Schmidtea mediterranea* [33] and probably the parasite *Schistosoma mansoni* [34]. It is, however, present at low levels in *Macrostomum lignano* [35], where both DNMT1 and DNMT3 are conserved. Rotifers and platyhelminthes have fast molecular and genomic evolution rates, and thus, their divergent DNA methylation patterns are unlikely to represent the ancestral spiralian condition.

Molluscs and annelids are species-rich spiralian clades with more conservatively evolving genomes [28]. In molluscs, studies of DNA methylation have primarily focused on bivalves (e.g., the oyster *Crassostrea gigas*) and cephalopods [36–41], revealing generally low-to-moderate methylation levels (~ 10% mCG). Base-resolution profiling in adult tissues with whole-genome bisulfite sequencing confirmed a mosaic 5mC landscape that concentrates in gene bodies of highly expressed genes and young genic TEs of bivalves but not cephalopods [39, 41]. In contrast, some annelids display moderate to high (~ 40–80%) methylation levels, such as nereidids (e.g., *Platynereis dumerilii* and *Alitta succinea*) and perhaps the deep-sea siboglinid *Riftia pachyptila* [8, 36]. However, these methylation levels are not substantiated with a reference genome and unbiased genome-wide data. Yet, other deep-sea annelids have lower global 5mC levels (~ 20%) [42]. Genome-wide 5mC landscapes available for adult tissue of the deep-sea annelids support the enrichment of this epigenetic mark in actively transcribed gene bodies, but not TEs [42]. Notably, treatments with cytidine analogs aimed at inhibiting DNMTs impair molluscan development and annelid posterior regeneration [8, 40]. Therefore, despite their different methylation levels, molluscs and annelids display canonical mosaic methylation, unlike rotifers and most platyhelminthes. However, the limited taxonomic sampling, especially at critical nodes of the molluscan and annelid phylogeny, and the lack of temporal resolution for genome-wide methylomes during the life cycles of these animals prevent identifying how dynamic this epigenetic mark is, thereby hampering the reconstruction of DNA methylation evolution in Spiralia, and indeed Bilateria generally.

**Fig. 1:**
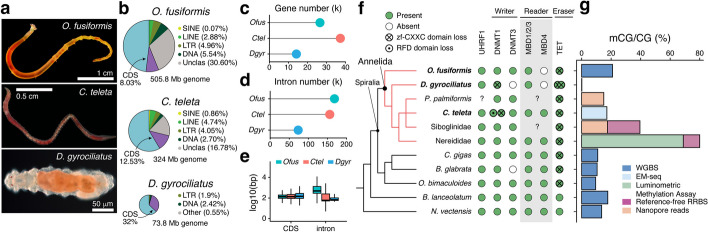
Annelids have different global DNA methylation levels. **a** Photographs of the three studied annelids. **b** Pie charts in scale represent genome size and percentage of repetitive elements and coding regions in *O. fusiformis*, *C. teleta*, and *D. gyrociliatus* genome*s*. Pie charts are scaled to genome size. **c**, **d** Dot plots comparing the number of genes and introns in the three focal taxa. **e** Box plots indicate the differences in coding sequence and intron lengths between the three annelid species. **f** On the left is a cladogram of the annelid lineages with existing global genome-wide 5mC and other representative metazoans. On the right is the composition of the DNA methylation toolkit for the lineages on the left. **g** Bar plot representing the global methylation levels for representative annelid and animal lineages, color-coded based on the methodology used

To address this gap, we comprehensively characterized the DNA methylation landscapes of three annelid species with distinct genomic features and phylogenetic positions within Annelida currently used as developmental model systems (Fig. 1a–e). *Owenia fusiformis* belongs to the sister clade of all remaining annelids [43], and its slow-evolving genome with ancestral indirect larval development has helped to infer ancestral characters to Annelida [29, 44]. *Dimorphilus gyrociliatus* underwent morphological miniaturization, has secondarily evolved direct development, and has one of the smallest known genomes for a free-living animal, almost devoid of TEs [45] (Fig. 1b). Finally, *Capitella teleta* has an indirect life cycle that can be closed in the lab and exhibits a slow-evolving genome [46, 47]. Our findings using base pair resolution genome-wide profiling demonstrate that 5mC levels vary during annelid development and across the annelid phylogeny, with *O. fusiformis* and *C. teleta* displaying moderate levels and a typical mosaic pattern whereas *D. gyrociliatus* showing negligible methylation as adults. DNA methylation positively correlates with transcriptional levels and stability, and normal methylation levels are essential for successful embryogenesis in *O. fusiformis*. However, the global DNA methylomes erode during the development and aging of these annelids. Altogether, our data reveal a dynamic DNA methylation landscape in Annelida, indicating that age-dependent methylation around active gene bodies is an ancestral genome regulatory state for bilaterians, with recurrent transitions to hyper- and unmethylated states being more frequent than anticipated.

## Results

### The developmental dynamics of the DNA methylation toolkit in Annelida

To investigate the evolution of DNA methylation in Annelida, we first characterized the repertoire of genes involved in transferring methyl groups to cytosines in DNA (DNMT1 and DNMT3 genes) and reading (MBD genes) and erasing (TET genes) this base modification in the genomes of *O. fusiformis*, *D. gyrociliatus*, and *C. teleta* (Fig. 1f; Additional File 1: Fig. S1–3). Consistent with previous results in the polychaete *P. dumerilii* and the leech *Helobdella robusta* [8], these three annelids have a DNMT1 gene, which in *C. teleta* is duplicated (Fig. 1f; Additional File 1: Fig. S1). Notably, only the DNMT1 of *O. fusiformis* has a protein domain composition identical to the human DNMT1 (Additional File 1: Fig. S4a). In *C. teleta* and *D. gyrociliatus*, the DNMT1 genes lack the most N-terminal DMAP domain (Additional File 1: Fig. S4a). In addition, the DNMT1 of *D. gyrociliatus* and one of the paralogs of *C. teleta* lack the zinc finger CXXC domain, while the other paralog of *C. teleta* has this domain but not the DNMT1-RFD (Additional File 1: Fig. S4a). However, despite these divergences in domain composition, the DNMT1 genes of these three annelids have conserved residues in the functionally active motifs of the methylase domain and could thus potentially methylate DNA (Additional File 1: Fig. S4b). UHRF1, a protein that recruits DNMT1 to hemimethylated DNA at replication foci during S-phase [48, 49], is present in all three species. Despite the duplication in DNMT1, the *C. teleta* UHRF1 has a domain composition like that of humans (Additional File 1: Fig. S4c). DNMT3, absent in *D. gyrociliatus*, shows a conserved protein domain architecture in *O. fusiformis* and *C. teleta* (Fig. 1f; Additional File 1: Fig. S1; Additional File 1: Fig. S4d). As for the readers, the three species have an MBD1/2/3 gene (Additional File 1: Fig. S2). Like *P. dumerilii* and *H. robusta* [8], *C. teleta* also has an MBD4 ortholog, absent in *O. fusiformis* and *D. gyrociliatus* (Additional File 1: Fig. S2). Additionally, these annelids have a TET gene, duplicated in *D. gyrociliatus* (Additional File 1: Fig. S3), and all have a conserved catalytic HxD motif. Like many other invertebrates and the TET2 paralog of humans, the TET genes lack a zinc finger CXXC domain in annelids (Fig. 1f; Additional File 1: Fig. S4f), which might affect its specificity and suggests ancestral or recurrent losses of this domain in Spiralia. Therefore, our findings support that a conserved DNA methylation machinery is an ancestral character of Annelida [8, 42]. Yet, this complement has diverged in specific lineages, particularly regarding the structural domain composition of DNMTs.

**Fig. 2:**
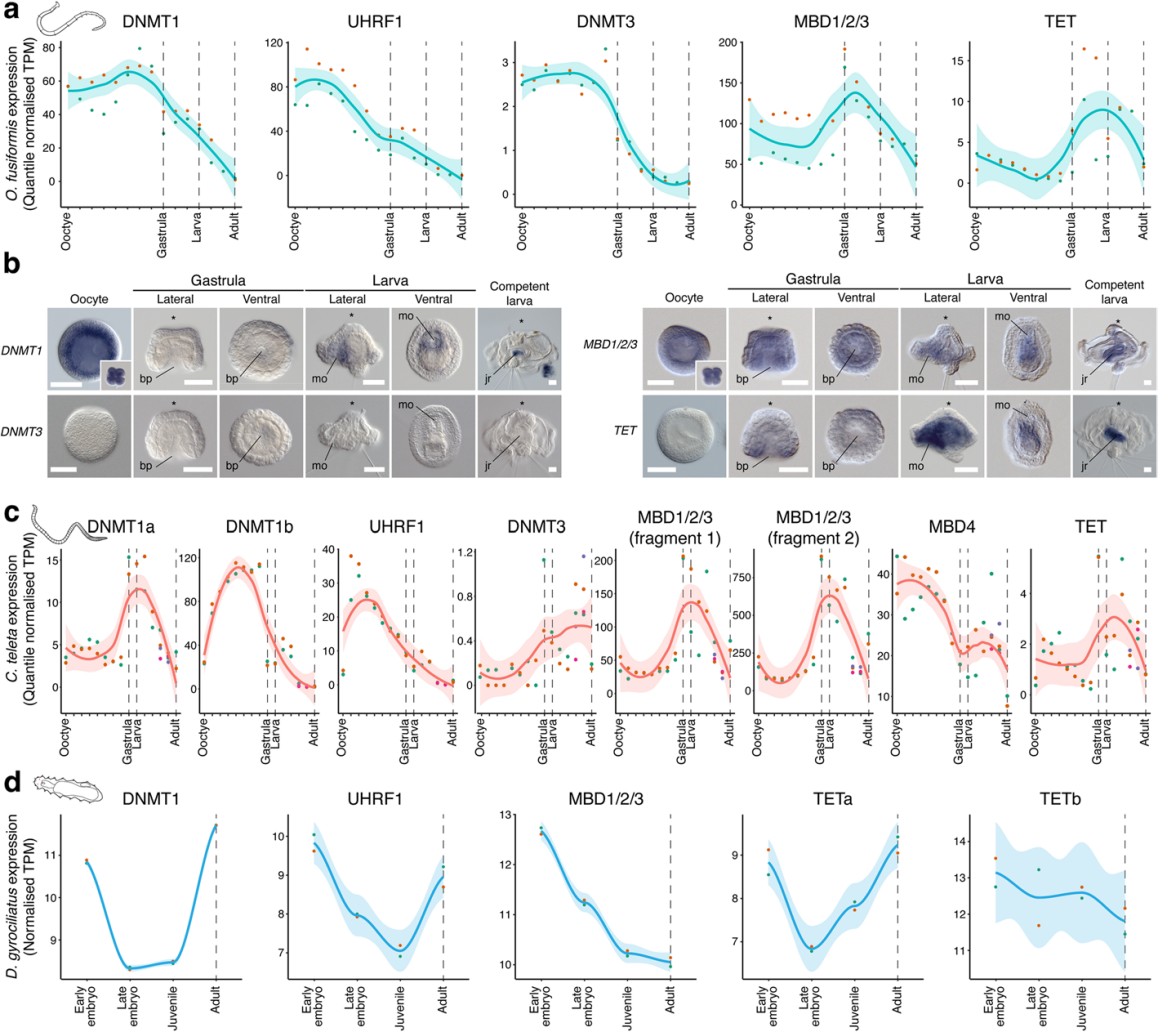
The temporal and spatial expression dynamics of the DNA methylation toolkit in Annelida. **a** Line plots depicting the temporal expression dynamics from the active oocyte to the adult stage for each gene of the DNA methylation toolkit in the annelid *O. fusiformis*. **b** Photographs of whole-mount in situ hybridization of *DNMT1*, *DNMT3*, *MBD1/2/3* and *TET* genes during the embryogenesis of *O. fusiformis*. *DNMT1* is strongly expressed in the oocyte and cleavage stages. Its expression decays during embryogenesis and is detected in the ventral side of the early larva and juvenile rudiment of the competent larva. *DNMT3* is expressed at shallow levels (**a**) and undetected by in situ hybridization. *MBD1/2/3* is expressed broadly at all stages of embryogenesis and concentrated mainly in the ventral side of the early larva and juvenile rudiment at the competent stage. *TET* is not detected during early embryogenesis and is expressed in the gut and ventral side of the early larva and juvenile rudiment of the competent larva. **c**, **d** Line plots depicting the temporal expression dynamics from the active oocyte to the adult stage in *C. teleta* (**c**) and from early embryogenesis to the female adult in *D. gyrociliatus* (**d**) for each gene of their respective DNA methylation toolkits. In **a**, **c**, and **d**, the stages sampled for genome-wide methylomes are indicated with dotted vertical lines. Scale bars in **b**, 50 µm

The DNA methylation toolkit is dynamically expressed during the life cycles of *O. fusiformis*, *C. teleta*, and *D. gyrociliatus*. In these three species, DNMT1 transcripts (*DNMT1b* in *C. teleta*) are more abundant in early than late embryogenesis (Fig. 2a, c, d) and broadly mirror the transcriptional dynamics of *UHRF1*. *DNMT3* genes are expressed at low levels (Fig. 2a, c), and the *MBD1/2/3* readers show a peak of expression during gastrulation and early embryogenesis (Fig. 2a, c, d). However, *TET* genes increase their expression right before gastrulation and peak at larval stages in *O. fusiformis* and *C. teleta* and the adults of *D. gyrociliatus* (for *TETa*) (Fig. 2a, c, d). Therefore, DNMT and TET genes exhibit inverse expression dynamics during annelid embryogenesis, as observed in some invertebrate deuterostomes [23].

To validate these temporal transcriptional dynamics, we characterize the spatial expression patterns of the DNA methylation toolkit during *O. fusiformis* embryogenesis. *DNMT1* is detected in the oocyte and fast-cycling blastomeres during early cleavage in this annelid (Fig. 2b). While no expression pattern was evident during gastrulation and late embryogenesis, *DNMT1* is localized in the early larva’s ventral and foregut regions and the competent larva’s juvenile rudiment (Fig. 2b). This differential expression is consistent with the expression pattern of *PCNA* in the early and competent larvae (Additional File 1: Fig. S5a). However, DNA replication and mitoses are widespread at the early larva stage (Additional File 1: Fig. S5c), suggesting that expression domains of *DNMT1* and *PCNA* might reflect areas of higher expression and that undetected base levels for these genes likely occur in most, if not all, cells. No expression pattern was detected for the lowly expressed *DNMT3* in *O. fusiformis* (Fig. 2b), whereas *MBD1/2/3* is ubiquitously detected during embryogenesis and later localizes around the foregut, ventral side, and juvenile rudiment during early and late larval stages (Fig. 2b). Finally, *TET* first becomes diffusely expressed in the gastrula of *O. fusiformis*. It is strongly detected in the gut and ventral region of the early larva and juvenile rudiment of the competent larva (Fig. 2b). DNA methylation writers (DNMT1 and DNMT3) and erasers (TET) thus localize to proliferative and differentiating tissues, suggesting a potential role of DNA methylation in regulating transcriptional programs in this animal group.

### Annelida possess the whole diversity of animal methylation patterns

To confirm the presence of DNA methylation in *O. fusiformis*, *D. gyrociliatus*, and *C. teleta*, we generated base pair resolution, genome-wide methylomes for the adults of these three species (Additional File 2: Table S1). In their adult forms, *O. fusiformis* and *C. teleta* contain 5mC in 20.92% and 17.1% of their CGs, respectively (Fig. 1g). However, *D. gyrociliatus* has negligible 5mC levels (0.16%), below the bisulfite non-conversion rate (Fig. 1g). Although this does not exclude the potential occurrence of DNA methylation at earlier stages of the life cycle in this miniature annelid, the ratio of observed vs expected genomic CGs in *D. gyrociliatus* is close to equilibrium (0.9; Additional File 2: Table S2), typical of unmethylated/lowly methylated species, suggesting lack of CG methylation despite encoding DNMT1 (Additional File 1: Fig. S1; Additional File 1: Fig. S4a). The DNA methylation levels observed in *O. fusiformis* and *C. teleta* coincide with those reported in other invertebrate lineages (10–20% CpG methylation levels), within (e.g., the molluscs *C. gigas*, *B. glabrata* and *O. bimaculoides*) and outside of Spiralia (e.g., the lancelet *B. lanceolatum* or the sea anemone *N. vectensis*). However, these levels starkly contrast with those of some annelid lineages belonging to Nereididae, such as *P. dumerilii* and *Alitta succinea*, which have well over 60% of their CGs methylated (Fig. 1g) [8, 36], albeit the available data for these species is not genome-wide. Therefore, Annelida shows diverse levels of DNA methylation, from the possibly ancestral condition of low-to-moderate levels observed in *O. fusiformis* and *C. teleta* to the loss of this base modification in *D. gyrociliatus* and the hypermethylated state in some lineages.

### Global methylation levels decrease and uncouple from transcription during development

**Fig. 3:**
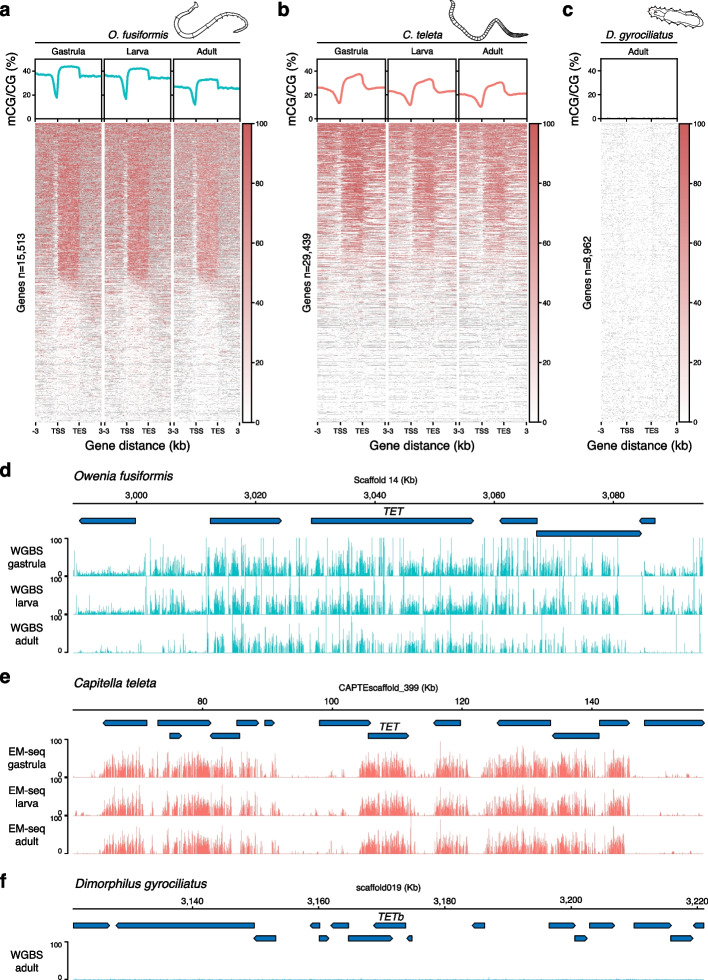
The dynamic methylation landscape in Annelida. **a**–**c** Metagene profiles (top) and heatmaps (bottom) of 5mC levels 3 kb upstream and 3 kb downstream of the gene bodies of *O. fusiformis* (**a**), *C. teleta* (**b**), and *D. gyrociliatus* (**c**) in all sampled stages. **d**–**f** Genome browser views showing 5mC levels (binning size 75 bases) around the *TET* locus in the three focal annelid taxa in all sampled stages. Transcriptional units are represented by blue boxes ending in an arrowhead that marks the direction of transcription

**Fig. 4:**
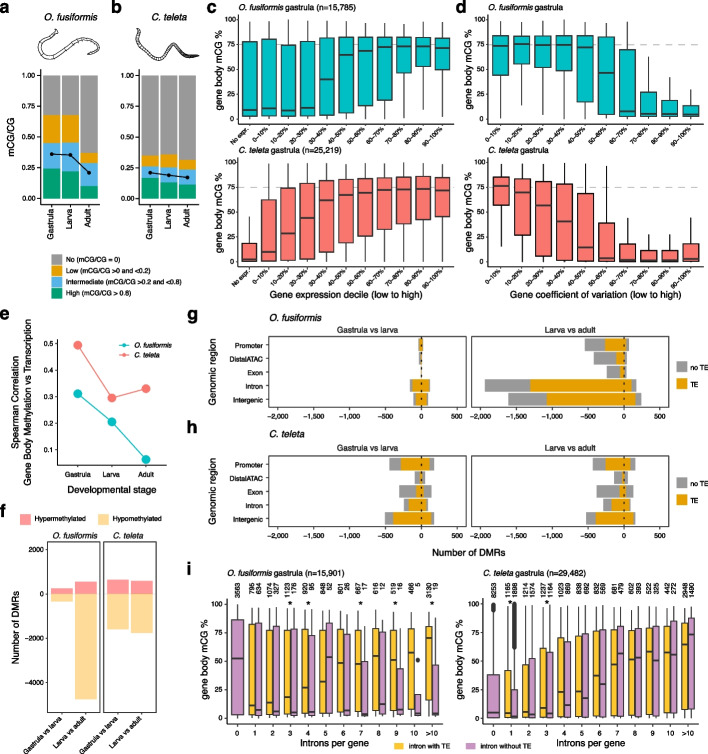
DNA methylation correlates with high and stable transcription.** a**, **b** Global CG methylation levels decrease during *O. fusiformis* (WGBS, **a**) and *C. teleta* (EM-seq, **b**) life cycles. Only CpGs with > 10 × coverage are depicted. **c** Box plots correlating gene body methylation and expression levels in *O. fusiformis* (top) and *C. teleta* (bottom) at the gastrula stage. Genes were divided into ten deciles and non-expressed (TPM < 1). **d** Box plots correlating gene body methylation and transcription stability in *O. fusiformis* (top) and *C. teleta* (bottom) at the gastrula stage. The coefficient of variation was calculated for all genes to rank them in 10 deciles from lower to higher values. Genes with a lower coefficient of variation (i.e., more transcriptionally stable) have higher gene body methylation levels in both species. **e** Correlation between gene body methylation and transcription in *O. fusiformis* (green line) and *C. teleta* (red line). As the life cycle progresses, gene body methylation and transcription are less strongly correlated. **f** Bar plots indicating the number of differentially methylated regions (DMRs) between pairwise comparisons during *O. fusiformis* (left) and *C. teleta* (right) life cycles. As expected by the gradual de-methylation, most DMRs represent a transition to a hypomethylated state. **g**, **h** Bar plots indicating the genomic annotation of the DMRs in *O. fusiformis* (**g**, top) and *C. teleta* (**h**, bottom). Most hypomethylated DMRs have a transposable element (TE) except in exonic and distal regulatory regions. **i** Box plots showing the effect of intronic transposable elements (TEs) in gene body methylation levels in *O. fusiformis* and *C. teleta* at the gastrula stage. Transcriptional units with an intronic TE show higher gene body methylation levels than equivalent genes without a TE in *O. fusiformis* but not *C. teleta*. Asterisks indicate *p*-values < 0.01 (Wilcoxon two-sided tests)

Methylated CpGs show a mosaic distribution in *O. fusiformis* and *C. teleta*, concentrated within gene bodies (Fig. 3a, b, d, e). In both species, the promoter region upstream of the transcription start site (TSS) is hypomethylated, and methylation levels increase between 1.1- and 1.2-fold in gene bodies compared with the background intergenic regions (Fig. 3a, b; Additional File 1: Fig. S6a, b). However, 5mC levels are higher in introns than in coding exons in *O. fusiformis*, while the opposite occurs in *C. teleta* (Additional File 1: Fig. S6a, b). In *O. fusiformis*, whose genome assembly is chromosome scale, DNA methylation levels are lower in the smallest chromosomes and do not correlate with the gene number in each chromosome (Additional File 1: Fig. S6c). To investigate whether the DNA methylation profiles observed in the adults of *O. fusiformis* and *C. teleta* were representative of their entire life cycles, we additionally generated whole-genome methylomes at one embryonic (gastrula, as the time point when DNMTs and TET change transcriptional dynamics) and one larval stage. For *O. fusiformis*, we also produced methylomes for oocytes and sperm as homogenous cell types. In both annelids, there are higher 5mC levels in gastrulae (36.1% in *O. fusiformis* and 20.99% in *C. teleta*) and larvae (35.42% in *O. fusiformis* and 18.93% in *C. teleta*) than in adults (Fig. 4a, b). Notably, 5mC levels in the gametes are comparable to those during embryogenesis and larvae of *O. fusiformis* (Additional File 1: Fig. S6d) and are higher than in the adult. Despite the differences between larvae and adults, the global pattern of CG methylation remains unchanged during the life cycle of these annelids, with a mosaic distribution concentrated in gene bodies and hypomethylated upstream promoters throughout (Fig. 3a, b, d, e). Therefore, the annelids *O. fusiformis* and *C. teleta* have typical invertebrate mosaic DNA methylation landscapes [1]. However, these erode as the life cycle progresses, as observed in two other phylogenetically distant deuterostome invertebrates [23].

To elucidate the interplay between gene body methylation (GbM) and gene expression, we first compared the GbM levels and transcriptional dynamics (transcriptional levels and stability) in *O. fusiformis* and *C. teleta*. Higher GbM correlates with higher gene expression values (Fig. 4c; Additional File 1: Fig. S7a, c) and transcriptional stability (Fig. 4d; Additional File 1: Fig. S7b, d) in both annelids, especially at the gastrula stage. This relationship between GbM and expression levels is also observed when only low and highly methylated gene bodies are considered (Additional File 1: Fig. S8a, b). Genes that do not follow this correlation, for example by being lowly methylated but highly expressed in the gastrula, are enriched for developmental categories (Additional File 1: Fig. S8c, d), suggesting that other variables, such as transcriptional stability, might account for the observed GbM levels. Genes in the highest expressed deciles tend to decrease their GbM levels more acutely than the rest as global methylation lowers during development (Additional File 1: Fig. S7a, c), with an overall decrease in the correlation between GbM and transcriptional levels as the life cycle progresses in both annelids, but especially in *O. fusiformis* (Fig. 4e; Additional File 1: Fig. S8a). Notably, the correlation between 5mC levels, gene expression, and transcriptional stability is also observed in the oocytes of *O. fusiformis* (Additional File 1: Fig. S6e, f), supporting the relationships between DNA methylation and gene expression dynamics observed in more cellularly heterogeneous late lifecycle stages. Therefore, DNA methylation and gene expression positively correlate in annelids, although this association weakens as the life cycle progresses.

The drastic change in global methylation levels from larval to adult stages could be involved in regulating specific developmental processes. To examine this hypothesis, we first identified the genes whose GbM levels varied the most between each time point of development in *O. fusiformis* and *C. teleta* (Additional File 1: Fig. S9). In *O. fusiformis*, 1860 genes (11.6% of the total) and 544 genes in *C. teleta* (1.84% of the total) showed large methylation changes, with 51 and 1809 genes being hyper- and hypomethylated, respectively, in *O. fusiformis* between adult and larval stages, and 125 and 419 genes being hyper- and hypomethylated in *C. teleta* at similar time points (Additional File 1: Fig. S9b, d). Transcriptionally, genes with decreased GbM in the *O. fusiformis* adult behave like the other methylated genes whose methylation status remains stable (Additional File 1: Fig. S10a). In contrast, unmethylated genes in the adult stage show a dynamic expression profile during the life cycle of *O. fusiformis*, with a sustained increase in average transcriptional levels from gastrulation to the juvenile and adult stages, thus indicating that GbM dynamics are not linked to transcriptional changes (Additional File 1: Fig. S10a) and that genes expressed later in development do not require GbM. In *C. teleta*, however, all genes increase their median transcriptional levels as the life cycle progresses, irrespectively of GbM (Additional File 1: Fig. S11a). Only a small fraction (between 12.8% and 25.54%) of genes that change their methylation status in *O. fusiformis* and *C. teleta* adults are differentially expressed between larval and adult stages (Additional File 2: Table S3). Remarkably, the variation in methylation status does not result in significant differences in the differential expression levels in the adults of either species (Figs. S10b and S11b). Further, in both *O. fusiformis* and *C. teleta*, the genes with variable GbM levels in the adults are related to diverse biological processes, from various metabolic processes in *O. fusiformis* (Additional File 1: Fig. S10c, d) to DNA recombination and regulation in *C. teleta* (Additional File 1: Fig. S11c, d). Therefore, changes in GbM during the annelid life cycle––particularly in the adult stage––are unlikely to result from the activity of specific developmental programs and present a poor correlation with transcriptional changes.

Global and gene-level methylation levels decrease during annelid development, yet the loss of methylation might occur preferentially in some regions, e.g., *cis*-regulatory elements [15–17, 23]. To assess this, we identified differentially methylated regions (DMRs) between the three developmental time points, and consistent with the global loss of DNA methylation, the vast majority of DMRs (86.3% and 73.4%) become hypomethylated in *O. fusiformis* and *C. teleta* adult stages, respectively (Fig. 4f). As expected by the extent of 5mC loss between adult and larval stages, the overall number of DMRs is more pronounced in *O. fusiformis* than in *C. teleta* (Fig. 4f). The majority of DMRs (83%) in *O. fusiformis* occur within non-coding (intergenic or intronic) and potentially non-regulatory regions, as they do not overlap with open chromatin as determined by existing ATAC-seq datasets (11.5% DMRs overlap distal ATAC peaks; Fig. 4g). In most of these cases (89.4%), the DMRs contain a TE. In *C. teleta*, hypomethylated DMRs in the adult are also most abundant in non-regulatory, intergenic regions (30.2%), followed closely by promoter regions and transcribed units (exons and introns) (Fig. 4h). As in *O. fusiformis*, the majority of DMRs in intergenic (76.3%), intronic (68.9%), and promoter (60.7%) regions in *C. teleta* include a TE (Fig. 4h). Notably, the temporal expression dynamics of the genes with a hypermethylated DMR in their promoters in the adult stage was not overtly different from those with a hypomethylated promoter DMR in any of the comparisons and annelid species (Additional File 1: Fig. S12a, b). Only a small fraction (between 3.9% and 26.25%) of genes with a promoter DMR are differentially expressed in the larvae and adults of *O. fusiformis* and *C. teleta* (Additional File 2: Table S4), without a consistent relationship between exhibiting a hyper- or hypomethylated promoter DMR and becoming up- or downregulated, respectively (Additional File 1: Fig. S12c, d). Likewise, the genes with a promoter DMR participate in various biological processes in both annelids (Additional File 1: Fig. S13). Therefore, these findings reinforce the observation based on GbM variation (Additional File 1: Fig. S10, S11) that changes in methylation status probably have context-specific or no direct causal effects on transcription in annelids, not following a straightforward link with developmental transcriptional repression or activation.

### DNA methylation is associated with intronic transposable elements in *O. fusiformis*

DNA methylation regulates the activity of transposable elements (TEs) in various animal lineages [50–53]. Because methylation levels decrease at adult stages in annelids, we tested whether this is linked to TE reactivation in these organisms. Consistently, in *O. fusiformis* and *C. teleta*, the most significant changes in TE expression occur between adult and larval stages. However, thousands of TEs are up- or downregulated (Additional File 1: Fig. S14a, f) regardless of their position and family. There is also not a general trend towards global TE upregulation (Additional File 1: Fig. S14b, c, e, g, h, i), which suggests a weak relationship between broad methylation levels and TE activity in these annelids (Additional File 1: Fig. S15) and instead, a link probably restricted to some specific contexts.

To discriminate whether 5mC might specifically target TEs in these annelids, we analyzed GbM levels of genes with and without intronic TEs. In *O. fusiformis* and *C. teleta*, GbM levels increase with the number of introns in an open reading frame (Additional File 1: Fig. S16a, c). Likewise, GbM levels in *O. fusiformis* increase as the number of TE-containing introns rises (Additional File 1: Fig. S16b). In *C. teleta*, however, this correlation is not apparent. While *C. teleta* genes with one to five TE-containing introns have higher GbM than those without, the GbM levels of genes with more than seven TE-containing introns are low (Additional File 1: Fig. S16d). Notably, the presence of a TE-containing intron rapidly raises the GbM levels compared to transcriptional units with the same intron number but no TEs in *O. fusiformis*, which again only applies to genes with fewer introns in *C. teleta* (Fig. 4i; Additional File 1: Fig. S16e, f). Therefore, these data indicate that GbM levels are linked to the presence/absence of TEs in *O. fusiformis*, suggesting that they might be specifically targeted in this annelid species. However, as previously proposed [52], this is probably not a widespread mechanism across invertebrates.

### Changes in transcriptional dynamics explain interspecies differences in DNA methylation

**Fig. 5:**
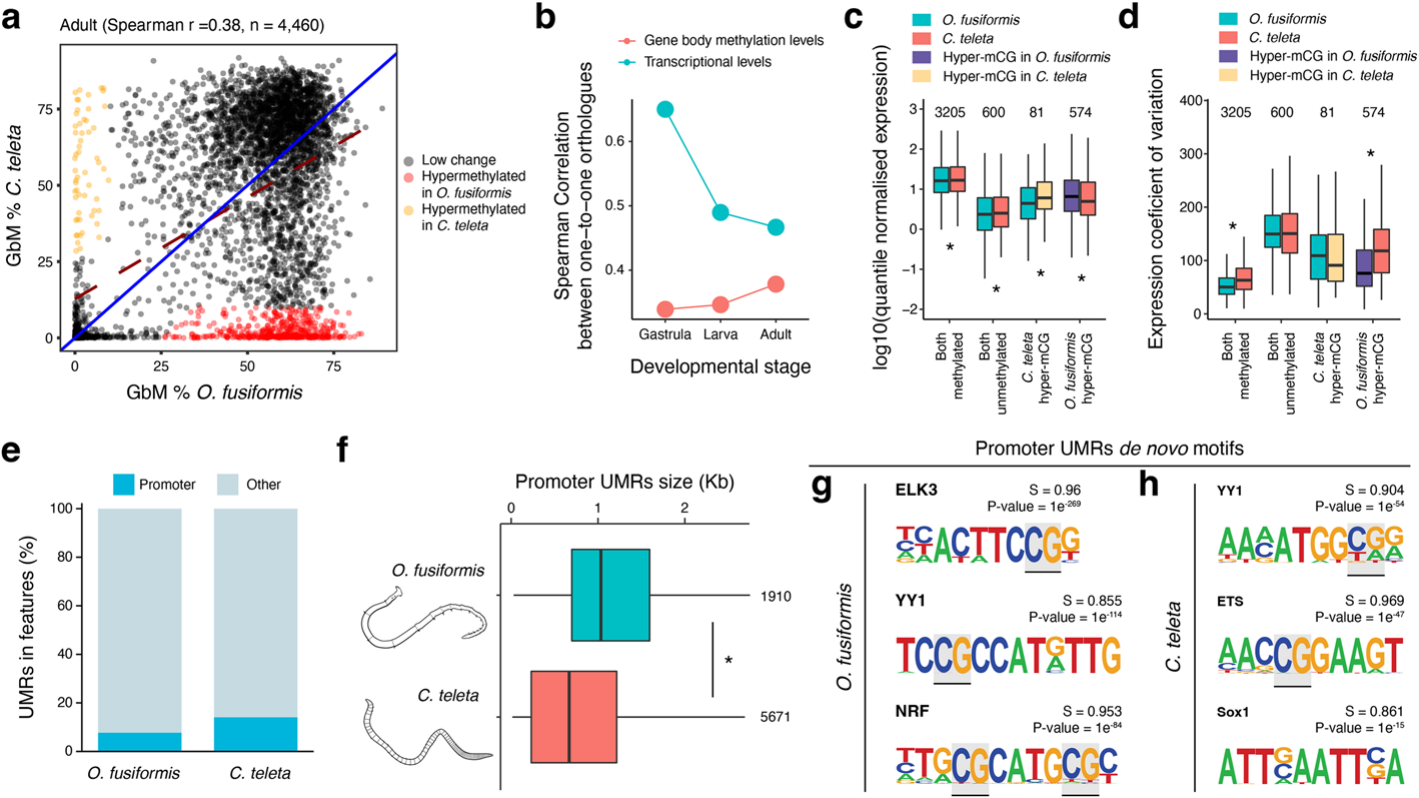
Interspecies differences in DNA methylation dynamics. **a** Scatter plots correlating gene body methylation (GbM) levels in one-to-one orthologous genes between *O. fusiformis* and *C. teleta* at the gastrula (left) and adult (right) stages. Hypermethylated genes in *O. fusiformis* compared to *C. teleta* are in red, and hypermethylated genes in *C. teleta* are in yellow. **b** Correlation in GbM and transcriptional levels between *O. fusiformis* and *C. teleta* at the three sampled stages. While the correlation in transcriptional levels in one-to-one orthologues decreases as the life cycle progresses, global GbM patterns become more similar with age between the two annelids. **c** Box plots of the distribution of expression values of pairs of one-to-one orthologues that are methylated (≥ 20% mCG) and unmethylated (< 20% mCG) in both annelids, hypermethylated in *O. fusiformis*, and hypermethylated in *C. teleta* as per panel (**a**). Consistent with the positive correlation between GbM and expression levels, hypermethylated genes in either annelid correlate with higher expression values on that species than the other one (asterisks represent *p*-value < 0.01, two-tailed *t*-test). **d** Box plots of the distribution of expression values of pairs of one-to-one orthologues that are methylated and unmethylated in both annelids, hypermethylated in *O. fusiformis*, and hypermethylated in *C. teleta*. Consistent with the positive correlation between GbM and expression stability, hypermethylated genes in either annelid correlate with a lower transcriptional variation on that species than the other one (asterisks represent *p*-value < 0.01, two-tailed *t*-test). **e** Bar plots showing the proportion of unmethylated regions (UMRs) in promoters and other genomic regions in *O. fusiformis* and *C. teleta*. Only a tiny fraction of UMRs colocalise with promoters. **f** Box plots showing the size distribution of promoter UMRs in the two studied annelids. Consistent with their different genome sizes, *O. fusiformis* has larger promoter UMRs than *C. teleta* (asterisk indicates *p*-value < 0.01, two-tailed *t*-test). **g**, **h** Transcription factor DNA binding motifs enriched in promoter UMRs in *O. fusiformis* (**g**, left) and *C. teleta* (**h**, right). Promoter UMRs are enriched in methylation-sensitive DNA binding motifs (potentially methylated CGs are highlighted in gray)

While the link between intronic TEs and GbM is restricted to *O. fusiformis*, the association between GbM and transcriptional dynamics does occur in this annelid and *C. teleta*. GbM has been proposed to conserve transcriptional dynamics of highly expressed genes, mainly with housekeeping functions, in a few invertebrate lineages [12]. To explore this in annelids, we compared the transcriptional dynamics and GbM levels of 4460 one-to-one orthologues between *O. fusiformis* and *C. teleta*. Most genes show conserved GbM status between the two species at gastrula (stage with highest 5mC levels) and adult (stage with lowest 5mC levels) stages (Fig. 5a). However, 11.5% of the orthologues are hypermethylated in *O. fusiformis* compared to *C. teleta* in adults. Still, only 1.2% are hypermethylated in *C. teleta* in the same stage, in agreement with the generally lower global GbM levels in this species (Fig. 5a). Interspecies differences in GbM affect broad functional gene categories, as identified through the enrichment of GO categories. Terms related to development, neurogenesis, and core cellular processes are overrepresented in the hypermethylated genes in the adult *O. fusiformis* (Additional File 1: Fig. S17a), whereas immunity, RNA biology, and metabolism are among the overrepresented GO terms in *C. teleta* (Additional File 1: Fig. S17b). Notably, and as previously observed [29], pairs of one-to-one orthologs tend to diverge in their transcriptional dynamics as the life cycle progresses in these two annelids (Fig. 5b). In contrast, the correlation of GbM levels between these gene pairs increases from gastrula to adult stages (Fig. 5b), as expected if GbM methylation is more frequently retained in stable, housekeeping genes that might show less interspecies transcriptional differences as development progresses. Indeed, the differences in GbM between *O. fusiformis* and *C. teleta* are partially attributed to interspecies differences in expression levels (i.e., hypermethylated genes in *O. fusiformis* are more highly expressed than their orthologs in *C. teleta* and vice versa) (Fig. 5c) and transcriptional stability (i.e., hypermethylated genes in *O. fusiformis* are less dynamically expressed during development than their orthologs in *C. teleta* and vice versa) (Fig. 5d). Therefore, the varying levels of global and GbM found in annelids are at least partially the result of species- and gene-specific transcriptional dynamics, reinforcing the connection between this base modification and gene expression in these organisms. However, it is possible that GbM might be a downstream consequence of gene transcription rather than a developmental regulatory mechanism of specific gene regulatory programs in these species.

Depletion of 5mC in upstream promoter regions is a common feature in many organisms that influences the binding affinity of methylation-sensitive transcriptional regulators [7, 54], which might ultimately explain the inter-specific differences in transcriptional dynamics and GbM between species. As such, most unmethylated regions (UMRs) in the hypermethylated genomes of sponges and vertebrates, for example, occur in promoters [7, 9]. However, in *O. fusiformis* and *C. teleta*, only 7.71% and 14.08% of the UMRs correspond with promoter regions (Fig. 5e), respectively. In agreement with the different genome sizes between these two annelids (Fig. 1b), the size of promoter UMRs is larger in *O. fusiformis* than in *C. teleta* (Fig. 5f). Further, in both species, these regions are enriched in transcription factor DNA binding motifs robustly annotated to 5mC-sensitive transcriptional regulators, such as ELK3, YY1, NRF, and ETS (Fig. 5g, h), as in other species [54]. Therefore, transcription factor methyl sensitivity shapes the regulatory information found in unmethylated promoters, which, together with the different dynamics of genome regulation during their embryogenesis [29], might contribute to the gene- and species-specific differences in DNA methylation between these two annelids.

### A gradual, age-dependent erosion of the post-embryonic methylome in *C. teleta*

**Fig. 6:**
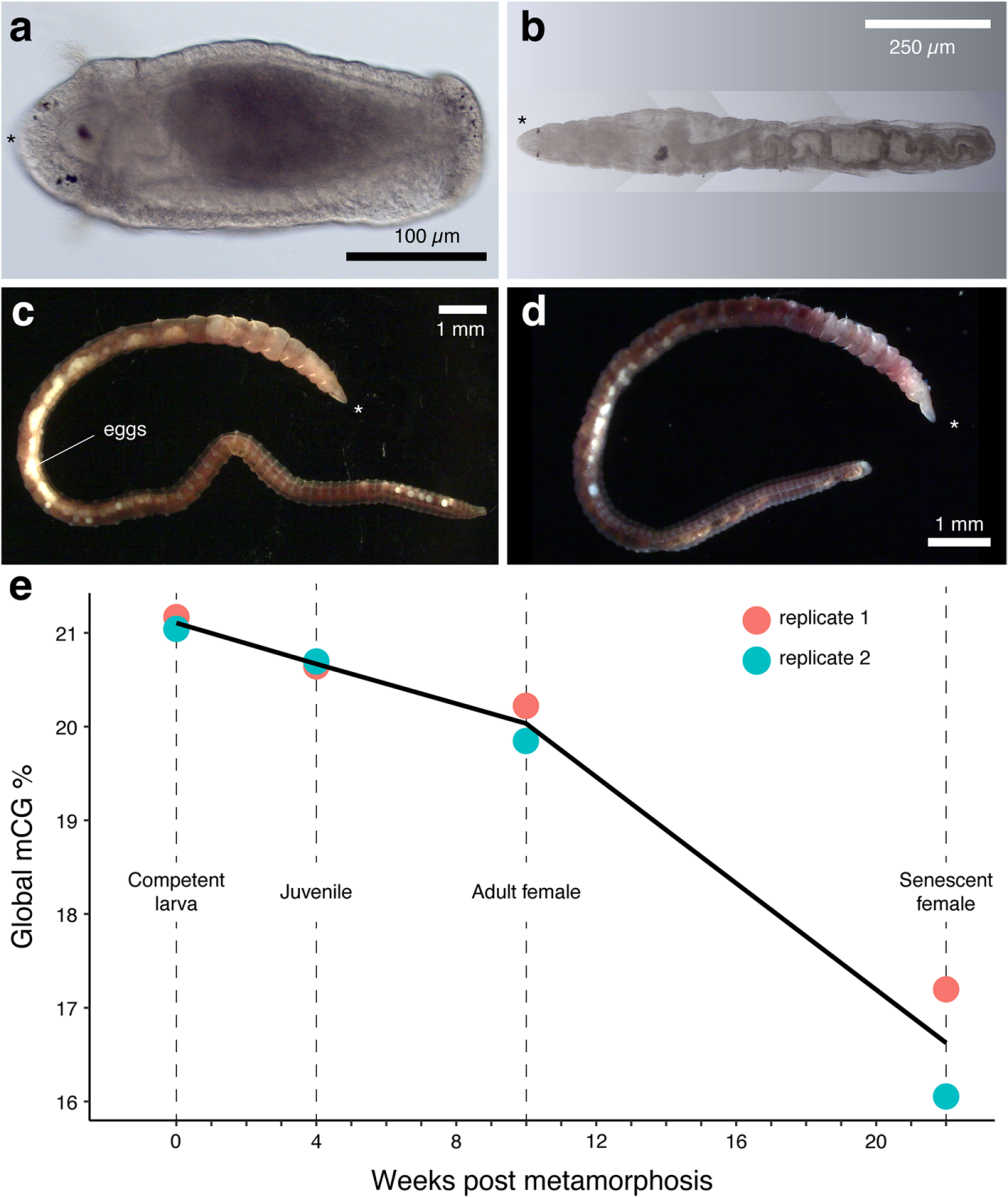
Gradual global demethylation in adult *C. teleta* with aging.** a–d** Photographs of the four sampled stages. **e** Line plot of global methylation levels obtained from multiplexed Nanopore libraries sequenced at shallow coverage from late larval stages until aging adults. The asterisks in **a**–**d** denote the anterior end

The global depletion of 5mC from embryonic to adult stages in *O. fusiformis* and *C. teleta* suggests that these organisms experience a developmental erosion of their methylome. However, this effect could extend post-embryonically in an aging-associated manner, as it is well-established for vertebrates [55–57]. To examine this, we generated replicated low-coverage Nanopore methylomes at four pre- and post-metamorphic ages in *C. teleta*. In this annelid, a lecithotrophic larva with adult-like characters in its late stages (Fig. 6a) [47] undergoes a minimal metamorphosis that results in a juvenile worm (Fig. 6b). After two months with *ad libitum* food and at 15 °C, these juveniles mature sexually and reach their reproductive peak at around three months post-metamorphosis (Fig. 6c). These adults reproduce several times during their lifetime, experiencing a progressive age-dependent anatomical decay (Fig. 6d). Consistent with the data from enzymatic methyl-seq (EM-seq) (Fig. 4b), global 5mC levels gradually decrease from the larva to juvenile stages in *C. teleta*. However, the loss of methylation is more acute as the animal undergoes several reproductive cycles and enters senescence (Fig. 4e). Therefore, post-embryonic age-dependent demethylation occurs in *C. teleta* and perhaps as well in other annelids like *P. dumerilii* [8], suggesting that the link between aging and epigenetic erosion might exhibit broad commonalities between distantly phylogenetically related animals. In the future, deep-coverage methylomes could elucidate whether this age-dependent demethylation concentrates in specific genomic regions and if there is any correlation between transcriptional dynamics with age in this annelid.

### Cytidine analogs disrupt embryogenesis in *O. fusiformis*

**Fig. 7:**
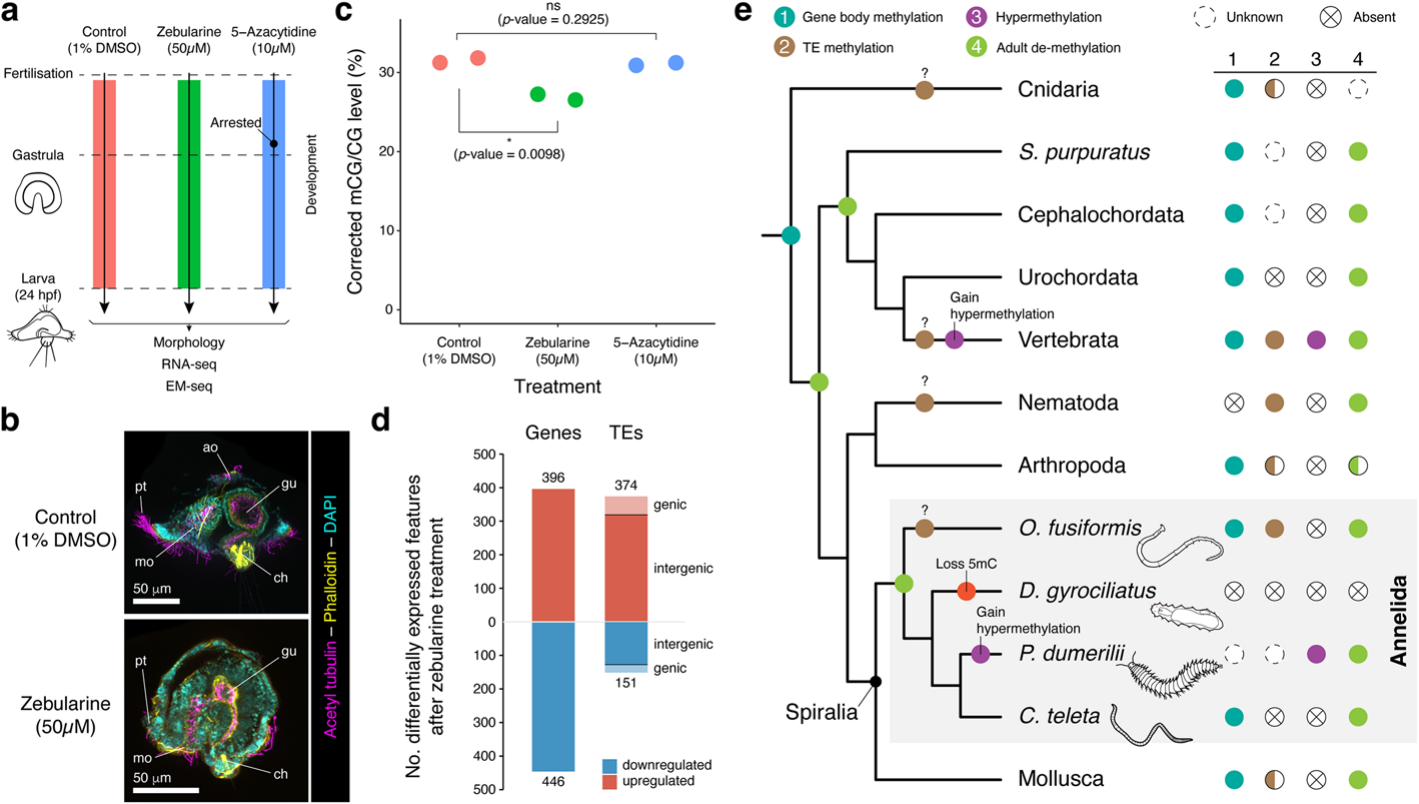
DNA methylation is essential for normal embryogenesis in *O. fusiformis*.** a** Schematic representation of the experimental design. Independent batches of embryos were treated after fertilization with either 1% DMSO (control) or 50 µM zebularine and 10 µM 5-azacytidine for 24 h until the larval stage, when phenotypes were scored. **b** Z-projections of confocal stacks of zebularine-treated and DMSO-control embryos fixed at the early larval stage and stained for acetylated tubulin (magenta), actin (yellow) and nuclei (cyan). Zebularine treatment impairs annelid embryogenesis as treated larvae fail to undergo normal organogenesis in *O. fusiformis*. **c** Zebularine but not 5-azacytidine treatment significantly decreases global methylation levels in *O. fusiformis* as measured with shallow coverage EM-seq (two-tailed unpaired *t*-tests). **d** Bar plots depicting the number of differentially expressed genes and transposable elements (TEs) after zebularine treatment. Reduction of normal methylation levels reactivates TE expression, mainly in intergenic regions. **e** The evolution of DNA methylation in bilaterally symmetrical animals and Spiralia. Gene body methylation is likely the ancestral stage in animals, and the presence of adult de-methylation in deuterostomes and annelids suggests that this might be an ancestral bilaterian feature. TE methylation might have evolved multiple times independently, similarly to the levels of DNA methylation, with numerous independent transitions to a hypermethylation and unmethylation state in different animal lineages

As an approach to functionally test the role of 5mC during annelid development, we treated fertilized oocytes of *O. fusiformis* until larval development with three cytidine analogs––zebularine, 5-azacytidine, and decitabine––that impair DNA methylation by incorporating into DNA and blocking DNMTs (Fig. 7a) [58–60]. Unlike in the annelid *P. dumerilii* [8], decitabine was toxic at all tested concentrations, blocking the division of the zygote. Thus, we did not use it in further analyses. With zebularine treatment, most treated embryos (74.3%) developed into abnormal larvae in a dose-dependent manner (Fig. 7b; Additional File 1: Fig. S18a; Additional File 2: Table S5, S6). As the control larvae, zebularine-treated larvae are bilaterally symmetrical and have a U-shaped gut and a blastocoel. Yet, treated larvae are smaller and less elongated than controls and have general differentiation problems, lacking a well-developed locomotory ciliated band, a usual number of posterior defensive chaetae, a sensory apical organ, and a prominent stomach (Fig. 7b; Additional File 1: Fig. S18a). Treatment with 5-azacytidine either killed the embryo (18%) or resulted in abnormal cleavage and gastrulation failure (82%), with the embryos becoming a disorganized mass with some ciliated cells (Additional File 1: Fig. S18a; Additional File 2: Table S6), indicating that 5-azacytidine, as decitabine, is probably toxic in this annelid. Only zebularine treatment causes a modest yet significant depletion (4.2%, *p*-value = 0.0098; two-sided *t*-test) of global 5mC levels during *O. fusiformis* embryogenesis (Fig. 7c; Additional File 2: Table S7). This depletion is widespread throughout the genome as expected from stochastic loss caused by DNMT sequestration, consistent with the lack of statistically significant DMRs between DMSO and zebularine treatment (FDR < 0.05, dmrseq, Additional File 1: Fig. S19a). Therefore, disruption of DNA methylation levels with zebularine leads to defective embryogenesis in the annelid *O. fusiformis*, although other cytidine analogs cause severe phenotypes without a clear impact on 5mC.

As expected from the treated phenotypes, zebularine impairs normal gene expression in *O. fusiformis*. Replicated transcriptomes of treated and control larvae (Additional File 1: Fig. S18b, c) revealed 396 upregulated and 446 downregulated genes after zebularine treatment (Fig. 7d). Upregulated genes exhibit higher average 5mC levels than downregulated genes (Additional File 1: Fig. S18d) and tend to be methylated (> 10% mCG) in control conditions (56%) more than downregulated genes (38%, *p*-value = 1.76e − 06, Wilcoxon two-sided test). Accordingly, they display more stable transcriptional dynamics during embryogenesis (Additional File 1: Fig. S18e). Modest loss of 5mC is observed in these genes upon zebularine treatment, yet similar levels of 5mC loss are observed for most genes (Additional File 1: Fig. S19b). While the upregulated genes are enriched in GO terms associated with morphogenesis and stimuli response, the downregulated genes are related to stress and ion homeostasis (Additional File 1: Fig. S18f, g), which might explain the smaller volume of the internal fluid-filled blastocoel. Zebularine treatment, however, largely upregulates TE transcription in *O. fusiformis* (Fig. 7d). Notably, most of the dysregulated TEs (85.29% of the upregulated and 84.11% of the downregulated) occur in intergenic regions (Fig. 7d), suggesting that their change in expression is unrelated to differences in gene transcription. In addition, LTRs are the largest upregulated TE class after zebularine treatment (Additional File 1: Fig. S18h). However, these are not the most abundant in the genome of *O. fusiformis* [29], which might reflect some degree of specificity in the regulatory impact of 5mC on TE activity in this annelid species. Additionally, most upregulated TEs were originally methylated in control conditions (72%, Additional File 1: Fig. S19e). Yet, both upregulated and downregulated TEs show overall decreased methylation levels following global mCG reduction (Additional File 1: Fig. S19f), suggesting that chromatin context and locus-specific regulation might dictate methyl sensitivity. Our findings thus functionally support that DNA methylation might be involved in gene and TE regulation during annelid embryogenesis. Still, we found that some originally unmethylated genes and TEs are also dysregulated upon zebularine treatment (Figure S19e), showing that there might be some secondary downstream effects independent of DNA demethylation. Our data and previous findings during posterior regeneration in the annelid *P. dumerilii* [8] indicate that normal levels of 5mC are essential for successfully deploying genetic programs during the annelid life cycle.

## Discussion

This study uses genome-wide, base-resolution profiling to characterize the landscape, dynamics, and function of DNA methylation during the life cycles of three annelids with distinct genomic features and phylogenetic positions, allowing us to reconstruct ancestral and derived traits for this base modification in a major invertebrate clade (Fig. 7e). All possible patterns of 5mC global methylation occur in Annelida, from the potentially hypermethylated genomes of some species, like *P. dumerilii* [8, 36], to the more typical invertebrate low-to-mid levels of *O. fusiformis* and *C. teleta*, and the loss of methylation in *D. gyrociliatus*. Given the DNA methylation levels of molluscs and other invertebrate groups [1, 2, 37], a hypermethylation state is likely a secondary modification in Annelida restricted, so far, to a single clade (Nereididae) (Fig. 7e), which will require confirmation with genome-wide techniques. The two copies of DNMT1 in *C. teleta* mirror the relatively unusual duplications of this gene observed in other animal lineages, such as some fish, marsupials, hymenopterans, and a sponge [7, 19, 61–63]. Interestingly, the two annelid paralogs show divergent domain compositions and expression dynamics, potentially reflecting a case of sub- or neofunctionalization, as might have also occurred with the opossum *DNMT1* paralogs [62]. In *D. gyrociliatus*, the secondary loss of 5mC (Fig. 7e) correlates with a modified DNA methylation toolkit, particularly with the absence of DNMT3 and a divergent N-terminus for DNMT1, which lacks the autoinhibitory zinc finger CXXC domain that recognizes unmethylated CGs [64, 65]. Nonetheless, this DNMT1 retains potential catalytic activity in its methyltransferase domain and an N-terminus replication foci domain (RFD), which drives the localisation of DNMT1 to the replication forks. This is reminiscent of the beetle *Tribolium castaneum*, whose genome also encodes a DNMT1 but lacks DNMT3 and similarly shows the absence of 5mC [66]. Still, in *T. castaneum*, DNMT1 knockdown impairs embryogenesis, suggesting that this gene plays essential roles beyond 5mC deposition in invertebrates and that DNMT1 loss might have pleiotropic effects in some lineages beyond modulating gene expression and 5mC maintenance [67].

As the life cycle progresses, the DNA methylation landscape erodes in *O. fusiformis* and *C. teleta*, with global and CpG level methylation levels falling along development and the correlation with transcription weakening. This aligns with decreased methylation levels between larval and juvenile stages in *P. dumerilii* detected by luminometric methylation assay [8]. 5mC erosion is consistent with inverse temporal patterns of gene expression between DNA methylation writers (DNMT1 and DNMT3), prevalent in early development, and erasers (TET), which upregulate after gastrulation and are highly and broadly expressed in later life stages. Similar adult demethylation and DNMT–TET transcriptional dynamics occur in vertebrates and related deuterostome invertebrates, such as amphioxus and sea urchins [16, 17, 23], suggesting that global demethylation as development progresses is a conserved epigenomic trait, at least for bilaterians (Fig. 7e). In vertebrates, this demethylation occurs in regulatory regions of developmental genes [16, 17, 23]. In deuterostome invertebrates, part of this demethylation occurs in intra-genic regulatory areas marked by ATAC-seq, but it is also widespread across the genome [23]. Although some regions that become hypomethylated during development overlap with potential *cis*-regulatory elements (ATAC-seq peaks) in annelids, this link appears weaker than in deuterostomes. Indeed, most adult-stage hypomethylated DMRs occur in intergenic and intronic regions that do not overlap ATAC peaks in *O. fusiformis* and *C. teleta*, and affect genes involved in diverse biological processes that are not restricted to developmental functions. This indicates that adult demethylation is probably a global dynamic rather than a tightly regulated process linked to the unfolding of specific developmental programs in these animals. In support of this scenario, TET proteins in annelids and other spiralians, but not deuterostomes, lack the N-terminus zinc finger CXXC domain (Fig. 1f) [7, 8, 23, 68, 69], which would confer DNA binding specificity to this DNA methylation eraser [70]. Interestingly, *O. fusiformis* sperm and oocyte methylation patterns resemble those of gastrula and show higher methylation levels than the adult. This might imply that the germline may preserve methylation levels better than somatic tissues during development or, alternatively, methylation is gained at some point during gametogenesis. Age-dependent changes in DNA methylation status at specific CGs are good predictors of biological age in mammals [71, 72], and global, genome-wide hypomethylation is a landmark of senescence in many vertebrate cell types [73]. Notably, the gradual, age-dependent DNA methylation erosion observed in annelids accelerates with aging, at least in *C. teleta*. Therefore, comparable manifestations of senescence occur in distantly related animals, opening the possibility that chronological “epigenetic clocks” [63] might exist more broadly than previously recognized in animals. More importantly, future studies comparing methylation across wild individuals should consider age as crucial in explaining global and GbM levels in invertebrates.

As in most invertebrates with mosaic DNA methylation patterns [1, 2, 6], 5mC accumulates in gene bodies, correlating positively with transcriptional activity and stability in annelids. Although the functional role of GbM is poorly understood, one of the main theories posits that it prevents spurious transcriptional initiation from otherwise highly transcribed regions [20]. The fact that intronic TEs influence GbM, at least in *O. fusiformis*, could support this hypothesis, as potentially, the TEs that land in these methylated regions are less likely to be detrimental and more likely to be kept, or DNA methylation is actively targeting these genes to avoid TE transcription [20]. However, *C. teleta* does not show an association between intronic TEs and GbM, indicating that the link between these features is probably evolutionarily plastic, as previously noted in cnidarians [52]. GbM might also be essential to keep high and robust transcriptional levels of the methylated genes in invertebrates [12]. This correlation with transcription stability is present in *O. fusiformis* and *C. teleta*, and the orthologues that differ in GbM levels across species also show divergent transcriptional levels and dynamics (loss of methylation linked to higher coefficient of variation). However, the zebularine treatment decreases global methylation in *O. fusiformis* yet does not result in general repression of genes with GbM. On the contrary, genes upregulated upon zebularine treatment show slightly higher methylation levels than those downregulated. This starkly contrasts what was observed after DNMT1 knockdown in the wasp *Nasonia vitripennis*, where the loss of GbM was associated with transcriptional downregulation, whereas unmethylated genes were upregulated [74]. This indicates that the causal link between GbM and transcription is still poorly understood in invertebrates and will require precise gene disruption strategies to disentangle the various proposed functions.

5mC TE methylation has been described in diverse animal taxa, from cnidarians to vertebrates and nematodes [11, 52, 53, 75] (Fig. 5e) and proposed to be a mechanism to suppress TE activity, particularly in lineages with high transposon burden [76]. However, the relationship between DNA methylation and TEs appears species-specific in most invertebrates. For instance, DNA methylation outside of gene bodies in molluscs occurs preferentially in some young TE classes in the bivalve *C. gigas*. Yet, the TE-rich genomes of cephalopods do not show strong TE methylation [39, 41]. Likewise, among all studied annelids, the association between DNA methylation and TEs, regardless of the class, is more pronounced and perhaps unique to *O. fusiformis* (Fig. 5e) [42]. Indeed, TE load does not correlate well with global 5mC levels and TE methylation in annelids: the deep-sea species *Paraescarpia echinospica* has a higher TE burden than *O. fusiformis* but does not show TE methylation [29, 42, 77]. Alternative mechanisms to control TE activity, such as the PIWI-interacting RNAs (piRNAs) [78, 79], are more broadly conserved in annelids and molluscs [80, 81]. In addition, although the mechanism of how DNMTs target gene bodies is reasonably well understood (the PWWP domain in DNMT3 orthologues binds to histone lysine 36 trimethylated residues typical of gene bodies) [82], how invertebrate DNMTs might target TEs remains unresolved. In tetrapods, for instance, DNMTs are targeted to TEs guided by KRAB zinc fingers, a fast-evolving transcription factor family that recruits various chromatin modification enzymes [83, 84]. However, vertebrate genomes are mostly hypermethylated by default; thus, targeting both old and new TEs is more straightforward to explain. Species like *O. fusiformis*, in which there is some degree of intergenic TE methylation, will be the best suited to reveal how TEs are targeted in genomes with mosaic methylation patterns. Nonetheless, given the current sampling among invertebrates and the relative scarcity of strong intergenic TE methylation in invertebrates, the convergent evolution of this mechanism across invertebrates and vertebrates from an ancestral animal pattern mostly restricted to gene bodies is the most parsimonious scenario (Fig. 7e).

Successful posterior adult regeneration and normal embryogenesis are impaired when using cytidine analogs in the annelid *P. dumerilii* and the bivalve *C. gigas*, respectively [8, 40]. In the annelid *O. fusiformis*, decreasing global DNA methylation levels with the cytidine analog zebularine also affects normal organogenesis, resulting in well-patterned larvae with immature organs. Notably, the phenotype described in *C. gigas* with 5-azacytidine––exogastrulation and development into disorganized ciliated cells [40]––resembles the outcome observed in *O. fusiformis* after an equivalent treatment. Still, it is likely a toxic effect because 5-azacytidine treatment does not affect normal 5mC levels. Unlike previous functional works in free-living spiralians, our study explores the amount of 5mC loss upon treatment and the transcriptional consequences of cytidine analogs during embryogenesis in *O. fusiformis*. Consistent with the TE methylation observed in this annelid, genome-wide hypomethylation during embryogenesis reactivates TEs, particularly LTRs in intergenic regions, but also dysregulates transcription. Although TEs in gene bodies tend to have higher methylation levels in *O. fusiformis*, more intergenic TEs are upregulated upon zebularine treatment, which might reflect a different chromatin state in those regions. For example, H3K36me3 is typically found in the gene bodies of methylated genes [85] and could be helping at inhibiting spurious transcriptional start sites within gene bodies [86]. These significant changes in gene transcription levels do not appear to affect specific gene programs or genes with determined GbM levels and result in overall even up- and downregulation of genes. This also agrees with the observed mild developmental phenotype, which affects the general morphology of the larva rather than the formation of specific tissues and organs, thus not suggestive of a role of 5mC in controlling regulatory networks. However, cytidine analogs are known to have toxic off-target effects [87], and their effects might thus reflect mild toxicity. Indeed, we observe strong phenotypes in *O. fusiformis* when using 5-azacytidine without observing global changes in 5mC, which is a cautionary tale when performing this kind of treatment in invertebrates. Species-specific sensitivity to cytidine analogs is probably more common than expected from vertebrate models [88]. Yet, the causal factor driving these differences in sensitivity across species remains unclear and is likely influenced by the different chemical properties of these molecules. In *O. fusiformis*, the reduction in global methylation levels when using zebularine is modest (4.2%), implying a lot of cell-to-cell variability in methylation loss, hampering the resolution of how this loss might influence transcription. Furthermore, the dysregulation of unmethylated genes and TEs upon zebularine treatment indicates that some downstream transcriptional effects are independent of 5mC. Further studies should incorporate treatments and approaches (e.g., CRISPR) with lower off-target toxicity effects and higher demethylation rates to help dissect the causal role of 5mC in invertebrate gene regulation and development.

## Conclusions

Our comprehensive, base-resolution characterization of the dynamic DNA methylation landscape during the life cycle of three annelids expands our knowledge of the evolution and roles of this pervasive base modification in one of the largest invertebrate clades. While a mosaic pattern of DNA methylation concentrated in active gene bodies is ancestral in annelids, this landscape has diverged in some lineages, with cases of potentially hypermethylated (Nereididae) and unmethylated (*D. gyrociliatus*) genomes. Likewise, methylation of transposable elements occurs in some lineages (*O. fusiformis*) but not others. However, the erosion of the DNA methylation landscape as the life cycle progresses is conserved in Annelida. Since this has also been observed in deuterostome invertebrates and insects, we suggest that developmental epigenetic erosion is an ancestral feature of bilaterians. Postembryonic methylation loss is associated with aging in the annelid *C. teleta*, implying that epigenetic clocks exist in non-vertebrates. Further research should encompass developmental genome-wide, base-resolution methylomes of more species, particularly those with divergent patterns, combined with direct manipulation of methylation patterns with gene disruption approaches to provide a more complete view of the functional implications of DNA methylation in annelids and invertebrate genomes.

## Methods

### Animal husbandry and embryo collection

Sexually mature *Owenia fusiformis* (Delle Chiaje, 1844) adults were acquired from the coasts near the Station Biologique de Roscoff (France) during the reproductive season and cultured in artificial seawater (ASW) at 15 °C as previously described [89]. Following in vitro fertilization [89], embryos were incubated and left to develop at 19 °C in filtered ASW until the desired embryonic and larval stages. *Capitella teleta* (Blake, Grassle & Eckelbarger, 2009) adults were cultured at 19 °C in ASW with embryonic and larval stages collected based on previously established protocols [47]. *Dimorphilus gyrociliatus* (O. Schmidt, 1857) were also cultured at 19 °C in a 4:5 ratio of ASW and freshwater, respectively [45].

### Orthology assignment and domain architecture analyses

DNMT, MBD, and TET sequences were mined from genomic and transcriptomic resources for *O. fusiformis*, *C. teleta*, and *D. gyrociliatus* [29, 45] and aligned against other representative sequences with MAFFT v7.505 in the L-INS-I strategy [90]. Resulting alignments were manually trimmed in Jalview v2.11.2.5 [91] according to the main domain boundaries of each gene family (PF00145 for DNMT, PF01429 for MBD, and PF12851 for TET), followed by removal of poorly aligned regions with TrimAl v1.4.rev15 in automated mode [92]. Maximum likelihood trees were then constructed with IQ-TREE v2.2.0.3 [93] using 1000 ultrafast bootstraps and the “-m TEST” option to identify the best amino acid substitution model for each gene family. Bayesian reconstruction was performed with MrBayes v.3.2.7a [94] using the LG + Gamma model (for DNMTs and TETs) and GTR (for MBDs) until convergence. All trees were visualized and edited in FigTree v.1.4.4 (https://github.com/rambaut/figtree/). The protein domain composition of each gene was characterized with InterProScan5 [95] and constructed in IBS 1.0 [96].

### Gene expression profile of DNA methylation-related genes

Expression dynamics of DNMT1, UHRF1, DNMT3, and TET genes were retrieved from publicly available temporal RNA-seq series for *O. fusiformis*, *C. teleta*, and *D. gyrociliatus* [29, 45]. Additionally, replicated RNA-seq libraries were constructed for the adult stages of *O. fusiformis* and *C. teleta*. Total RNA was extracted with the RNA Miniprep Kit (New England Biolabs, #T2010) according to the manufacturer’s instructions and used for standard strand-specific Illumina library prep, which was sequenced in a NovaSeq 6000 platform in pair-end 150 bases mode. Sequencing adaptors were trimmed with fastp v0.20.1 [97] and pseudo-aligned to gene models with Kallisto v0.46.2 [98]. DNA methylation-related gene expression profiles of *O. fusiformis* and *C. teleta* were plotted in R using ggplot2 v3.4.0 [99] based on quantile normalized transcripts per million (TPM) to account for technical and biological variations between libraries. Normalized counts were used to plot these gene expression profiles for *D. gyrociliatus*.

### Whole-mount in situ hybridization

DNMT1, DNMT3, MBD1/2/3, TET, and PCNA genes were amplified from cDNA containing various developmental stages of *O. fusiformis* through two successive rounds of nested PCR that added a T7 promoter at the 3’ end of the amplicon. The resulting DNA templates were in vitro transcribed to obtain DIG-labeled riboprobes using T7 enzyme (Ambion MEGAscript kit, #AM1334) and stored at − 20 °C in hybridization buffer at a total concentration of 50 ng/μl. Colorimetric whole-mount in situ hybridization was performed following established protocols [44]. Representative samples were imaged with a Leica DMRA2 upright microscope and Infinity5 camera (Lumenera) using differential interference contrast (DIC) options. The resulting whole images were adjusted for brightness, contrast, and color balance in Adobe Photoshop and assembled into a final figure panel in Adobe Illustrator.

### DNA methylation sequencing

Whole-genome bisulfite sequencing (WGBS) libraries were constructed for *O. fusiformis* (gastrula, larva, and adult) and *D. gyrociliatus* (adult) samples with single replicates each (Additional File 2: Table S1). Bisulfite conversion was performed using the EZ DNA Methylation Gold Kit (Zymo Research), followed by size selection and library amplification. Developmental time points (gastrula, larva, and adult) of *C. teleta* and oocytes and sperm of *O. fusiformis* were assayed by EM-seq (NEB #E7120) with single replicates following the manufacturer-provided protocol. EM-seq was preferred over WGBS for these samples and the validation of cytosine analog treatments (see below) for its lower input requirements and better preservation of DNA, which might reduce coverage biases [100]. For EM-seq negative and positive controls, spikes of unmethylated lambda phage DNA and methylated pUC19 DNA were added before enzymatic treatment, whereas only lambda phage DNA was used in WGBS. All the resulting libraries were sequenced in 150-base paired-end mode on an Illumina platform at deep coverage. Adaptors were trimmed with fastp v0.20.0 [97], aligned to their respective reference genomes, deduplicated, and methylation called for all cytosine contexts (CpG, CHG, and CHH) using Bismark v0.22.3 [101] with default options. Methylation signals were visualized in IGV v2.8.0 genome browser using bigwig files generated from Bismark bedGraph and coverage files via UCSC *bedGraphToBigWig* function. Visualizations of genomic tracks were plotted with pyGenomeTracks [102]. Bismark text files containing methylated and unmethylated cytosines were converted into CGmap files with CGmapTools v0.1.2 [103] and processed into bsseq objects in R for all further downstream analyses. Gene body and TE methylation values were filtered for a minimum mean read coverage of four and a minimal number of five CpGs per gene on both developmental and treatment samples. Non-conversion rates were calculated based on the methylation levels of spike-ins, mitochondrial genomes, and non-CpG contexts. Despite not being replicated, methylation levels inferred from developmental samples were consistent with those obtained from replicated assays generated to investigate age-dependent methylation changes and the effect of cytidine analogs in larvae (see below).

To characterize the age-dependent global demethylation in *C. teleta*, replicated samples of competent larvae (stage 8), 1-month juveniles, gravid females, and 22-week-old senescent females were collected. Genomic DNA was extracted with the MagAttract kit (Qiagen). Nanopore libraries were built with the Rapid barcoding kit (SQK-RBK114.24) following the manufacturer’s instructions and sequenced on MinION Mk1C using a MinION R10.4.1 flow cell (FLO-MIN114). The sequencing reads were then base-called and demultiplexed with Guppy (v6.2.1) using the super accuracy model dna_r10.4.1_e8.2_400bps_modbases_5mc_cg_sup.cfg model for methylated CpGs. Global methylation levels were calculated as previously described, using a genome skimming approach [104].

### Gene body methylation profiling

Positional heatmaps of mCG/CG levels in protein-coding loci were generated using the bssq R object described above and the *computeMatrix* function of deepTools2 [105] and plotted with *plotHeatmap* and *plotProfile* functions with the “scale-regions” option and a bin size of 100 bases. To correlate gene body methylation with transcription, transcriptomic data in TPM (transcripts per million) for *O. fusiformis* and *C. teleta* was imported and processed in R by ranking the mean TPM for each developmental stage and the coefficient of variation for each gene into deciles using the *quantile* function in R and mean_tpm ≥ 1 operation to exclude genes with mean TPM expression of less than 1.

Pairwise changes in GbM in both annelids were obtained only for genes with coverage > 4 in all samples. We first computed the average difference of GbM across all genes between two stages (e.g., gastrula vs larva) and identified the genes that deviated twofold from that average to focus on the genes with changes above the global methylation loss. We first obtained one-to-one orthologues from an Orthofinder2 run with default diamond parameters for inter-species comparisons [29]. Then, we selected the GbM levels from each orthologue in each species, only accepting orthologues with enough coverage threshold.

### Transposable elements

RepeatModeler2 [106] was used to identify repetitive regions in *D. gyrociliatus*, *C. teleta*, and *O. fusiformis*, and the resulting annotations were imported into R as GenomicRanges objects [107], including a filtering step to select for TEs longer than 400 bp. We further classified these TEs based on their location in genic or intergenic regions by overlapping them with the gene annotations. We identified their methylation status, with values equal to or greater than 20% defined as methylated. The RepeatModeler2 built-in script to calculate Kimura divergence values was used to estimate the evolutionary divergence of TE families. To investigate the relationship between TE density and GbM levels, we ranked genes based on their number of introns (including the presence or absence of TEs) and corresponding methylation levels using the GenomicFeatures v1.48.4 package [107]. TE expression was calculated with TElocal v1.1.1 (https://github.com/mhammell-laboratory/TElocal) and differential expression analysis in a pairwise manner using the DESeq2 v1.36.0 [108] package with the function *lfcShrink*. Differentially expressed TEs were selected based on an adjusted *p*-value ≤ 0.01 and log2 fold change ± 2.

### Unmethylated regions (UMRs)

Repositories containing genomic information were manually created for *O. fusiformis* and *C. teleta* using the R package BSgenome v1.64.0 [109]. Using BSgenome and methylomes in bsseq object form, UMRs were identified for each species using MethylSeekR v1.36.0 [110] *segmentUMRsLMRs* function with “meth.cutoff = 0.5” and “nCpG.cutoff = 4” parameters. Next, motifs of UMRs less than 5 kb overlapping promoter features (2 kb upstream and 200 bp downstream of the transcription start sites) were analyzed using HOMER v 4.11 [111] with *findMotifsGenome.pl* function and motif lengths of 6, 8, 10, and 12.

### Differentially methylated regions (DMRs)

The DSS v2.48.0 R package [112] was used to compute developmental DMRs in a pairwise manner, using the *callDMR* function retaining a minimum number of five CGs for these regions with the minCG = 5 and dis.merge = 100 parameters. We applied a more stringent delta = 0.2 parameter, which specifies a difference in methylation greater than 20%. The resulting DMRs were filtered to ensure a minimum mean coverage of four in these regions, and their genomic distributions were defined using the GenomicFeatures v1.48.4 R package [107]. For drug-treated DMRs, we used dmrseq [113], as it allows filtering for FDR (< 0.05) when replicates are available. Distal regulatory elements were defined as publicly available consensus ATAC peaks not overlapping promoters (− 1000/ + 200 bp from each transcriptional start site) [29]. The developmental expression profiles of the genes with promoter DMRs were plotted based on their log10 transformed stage-specific transcriptomic data. The enrichment of Gene Ontology terms in these genes was calculated with the TopGO R package [114]. All graphs were created with the ggplot2 v3.4.0 package [99].

### Inhibitor treatments

Fertilized oocytes of *O. fusiformis* were treated with 50 μM zebularine (Abcam: ab141264) and 10 μM 5-azacytidine (Abcam: ab142744) using equivalent volumes of dimethyl sulfoxide (DMSO) as a negative control. Treated and control embryos developed for 24 h at 19 °C until the larval stage, when drugs were washed off. As detailed above, two biological replicates collected for RNA-seq and EM-seq protocols were immediately snap-frozen in liquid nitrogen and stored at − 80 °C before library preparation and sequencing. Samples collected for immunohistochemistry were relaxed in an 8% magnesium chloride solution before fixation in 4% paraformaldehyde for 1 h at room temperature (RT). Samples were washed with 0.1% Tween-20 phosphate-buffered saline (PTw) and stored at 4 °C in PTW with 1 μM sodium azide.

### EdU and immunohistochemistry

F-actin and antibody staining of oocytes and control and treated *O. fusiformis* larvae were conducted as described elsewhere [89]. Briefly, samples were permeabilised with PBS + 0.1% Triton X-100 + 1% bovine serum albumin (PTx + BSA) and blocked in PTx + 5% normal goat serum (NGS). Samples were incubated with primary antibodies (mouse anti-acetyl-alpha tubulin antibody, clone 6-11B-1 (Millipore-Sigma Aldrich, cat#: MABT868, RRID: AB_2819178), rabbit anti-FMRF-amide antibody (Immunostar, cat#: 20091, RID: AB_572232), mouse anti-PCNA antibody (Cell Signaling Technology, cat#: 2586), and rabbit anti-phospho-Histone H3 (Cell Signaling Technology, cat#: 9701S)) diluted (1:500, except for anti-phospho-Histone H3 that was used at 1:1000) in PTx + NGS overnight at 4 °C and washed several times with PTx + BSA before overnight incubation with 1:800 diluted Alexa-labeled secondary antibodies (ThermoFisher Scientific), 1:100 Alexa Fluor™ 488 Phalloidin (ThermoFisher Scientific), and 5 µg/ml DAPI in PTx + NGS. Secondary antibodies were washed in PTx + BSA, and samples were cleared with 70% glycerol in PBS and stored at 4 °C before imaging. EdU was performed as described elsewhere [89], with an incubation time of 30 min. Representative samples were imaged with a Leica SP5 confocal laser scanning microscope in all cases. Resulting z-stack projections were made with Fiji [115], and these images were then edited in Adobe Photoshop and assembled into a final figure panel in Adobe Illustrator.

### RNA-seq profiling and differential expression analyses of control and treated samples

Adapter sequences and poor-quality bases were trimmed with fastp v0.20.1 [97] and aligned to the *O. fusiformis* reference genome and annotation [29] (GCA_903813345) using STAR vX [116] and Kallisto v0.46.2 [98], respectively. Resulting bam files were processed with TElocal v1.1.1 (https://github.com/mhammell-laboratory/TElocal) to obtain gene and transposable elements expression counts. Differential expression analyses between zebularine-treated and control samples were performed with the R package DESeq2 v1.36.0 [108] with a significant threshold adjusted to a *p*-value of ≤ 0.01 and a log_2_ fold change of ± 2. A Pearson correlation matrix of the libraries was computed with corrplot v0.92 (https://github.com/taiyun/corrplot), Gene Ontology enrichments were performed with TopGO [114], and all other plots were generated with ggplot2 v3.4.0 [99].

### Supplementary Information

Additional file 1: Supplementary Figures. Figures S1 to S19.

Additional file 2: Supplementary Tables.

Additional file 3. Review history.

## Data Availability

The datasets generated and analyzed during the current study are available in Gene Expression Omnibus (project accession number GSE250187) [117]. Scripts generated to analyze these datasets are available in a public repository on GitHub (https://github.com/ChemaMD/annelid5mC) [118] and Zenodo (10.5281/zenodo.12607959) [119] under an Apache License 2.0. This work also uses previously published sequencing data publicly available at the European Nucleotide Archive (project PRJEB38497) [29] and Gene Expression Omnibus (accession numbers GSE184126, GSE202283, GSE192478, GSE210813, and GSE210814) [29].
