# Supplementary material for: Annelid methylomes reveal ancestral developmental and aging-associated epigenetic erosion across Bilateria
Source: Genome Biol. 2024 Aug 1;25:204. doi: 10.1186/s13059-024-03346-z (PMC11292947; doi:10.1186/s13059-024-03346-z)
Supplement: Supplementary file 1 — Additional file 1: Supplementary Figures. Figures S1 to S19. [file 13059_2024_3346_MOESM1_ESM.docx]

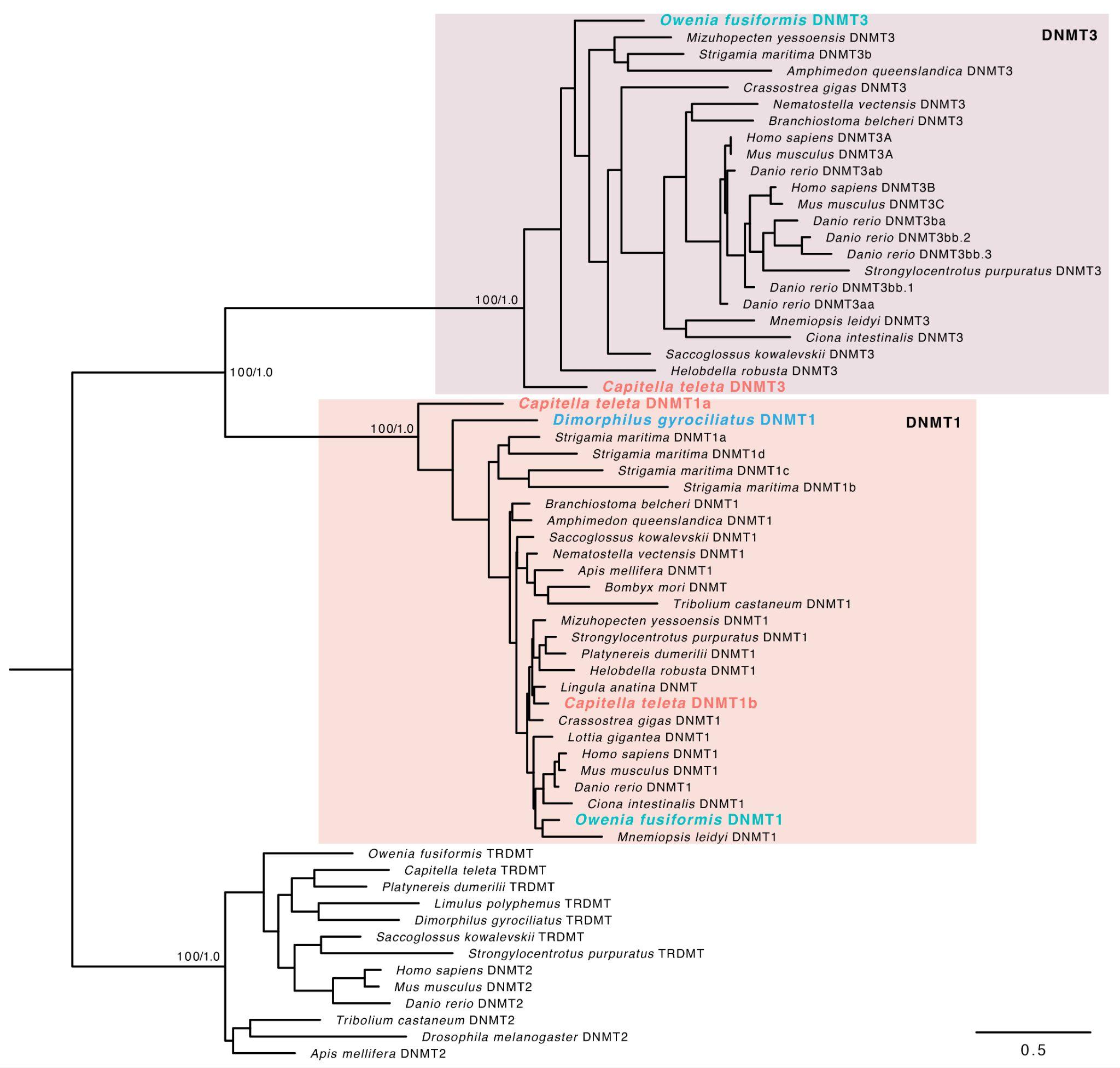


**Fig. S1 – Gene orthology assignment of DNMT genes.** Orthology assignment of DNA methyltransferases using the DNMT2 (also known as TRDMT), which predominantly methylates tRNAs, as the outgroup. The tree topology is based on maximum likelihood reconstruction, and node supports indicate both bootstrap values (from 0 to 100) and posterior probabilities (from 0 to 1) at key nodes. Boxes indicate the DNMT1 and DNMT3 sub-families, and the scale bar represents the number of amino acid substitutions per site alongside the branches.


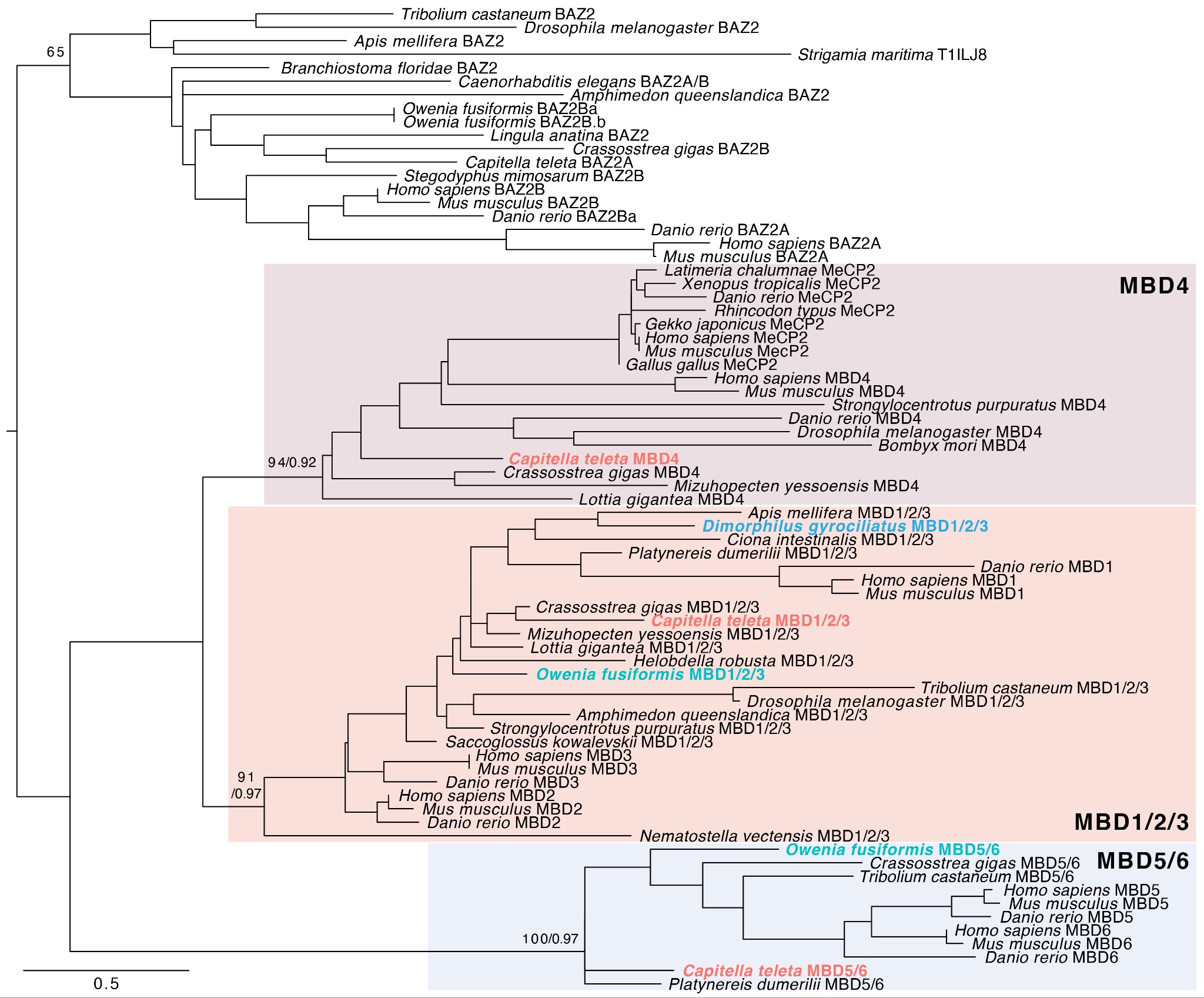


**Fig. S2 – Gene orthology assignment of MBD genes.** Orthology assignment of methyl-CpG-binding domain (MBD) proteins using the Bromodomain Adjacent to Zinc Finger Domain 2 (BAZ2) protein as outgroup. The tree topology is based on maximum likelihood reconstruction, and node supports indicate both bootstrap values (from 0 to 100) and posterior probabilities (from 0 to 1) at key nodes. Boxes indicate the MBD sub-families, and the scale bar represents the number of amino acid substitutions per site alongside the branches.


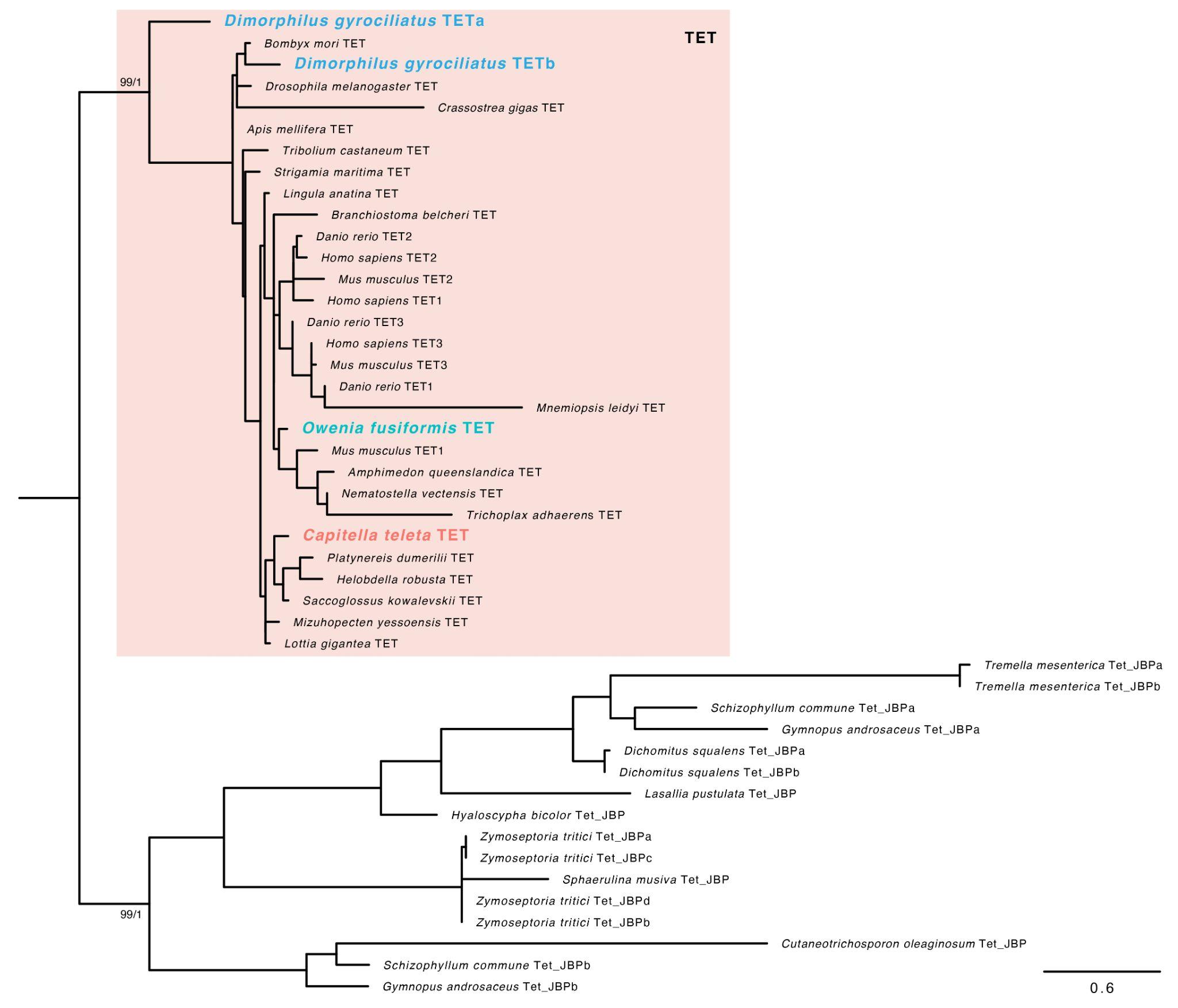


**Fig. S3 – Gene orthology assignment of TET genes.** Orthology assignment of ten-eleven translocation (TET) methylcytosine dioxygenases using the TET-domain containing fungal proteins as outgroup. The tree topology is based on maximum likelihood reconstruction, and node supports indicate both bootstrap values (from 0 to 100) and posterior probabilities (from 0 to 1) at key nodes. Boxes indicate animal TETs, and the scale bar represents the number of amino acid substitutions per site alongside the branches.


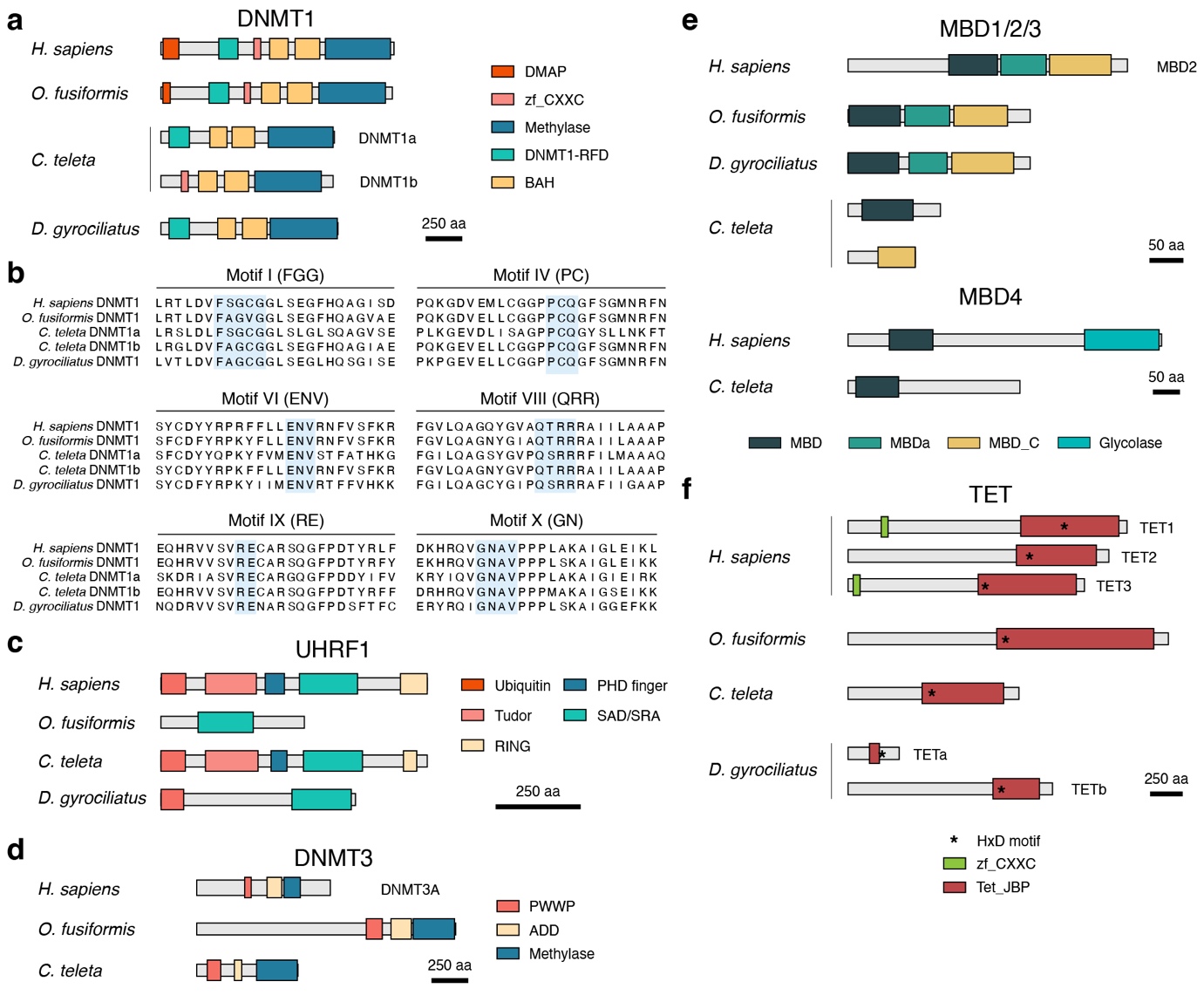


**Fig. S4 – The domain architecture of the annelid DNA methylation toolkit.** (**a**) Schematic drawings of the domain composition of DNMT1 genes in the three focal annelid species compared with the human orthologue. (**b**) Multiple protein alignments of the motifs of the DNA methyltransferase domain with catalytic activity comparing annelid (*O. fusiformis*, *C. teleta* and *D. gyrociliatus*) and human sequences. *Dimorphilus gyrociliatus* has conserved residues in these motifs and, thus, a potentially active domain. (**c**–**f**) Schematic drawings of the domain composition of UHRF1 (**c**), DNMT3 (**d**), MBDs (**e**) and TET (**f**) genes in the three focal annelid species compared with the human orthologue. In (**a**, **c**–**f**), drawings are to scale. Asterisks in (**f**) highlight the catalytic HxD motifs in TET proteins.


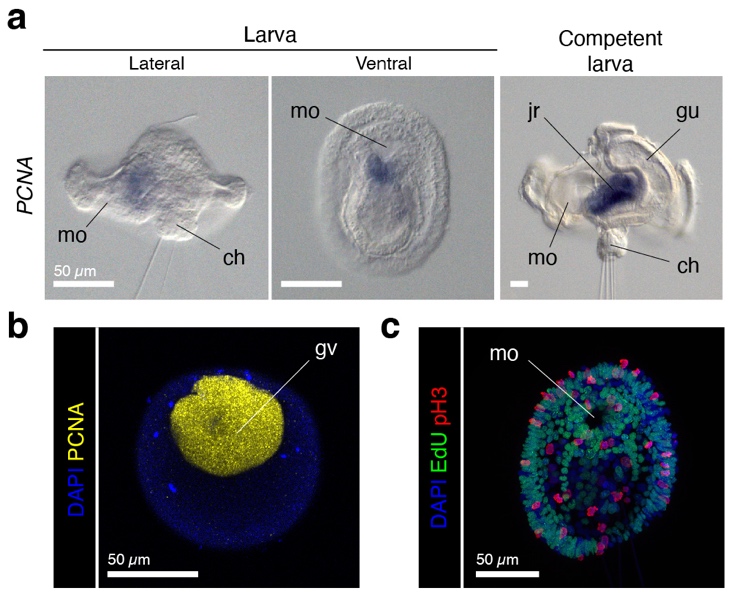


**Fig. S5 – DNA replication and cell divisions in *O. fusiformis* embryogenesis.** (**a**) Whole mount in situ hybridisation of PCNA in early and competent larvae of O. fusiformis. Expression is largely detected in the early larva's foregut and ventral regions and the competent larva's juvenile rudiment. This expression pattern mirrors the one of DNMT1 (see Fig. 2b). (**b**) PCNA protein localises in the germinal vesicle of *O. fusiformis* oocytes, consistent with the expression of DNMT1 at that stage (see Fig. 2b). (**c**) DNA replication (EdU positive cells) and cell divisions (phospho-Histone3 positive cells) are, however, widespread in the early larva. ch, chaetae; gu, gut; gv, germinal vesicle; jr, juvenile rudiment; mo, mouth.


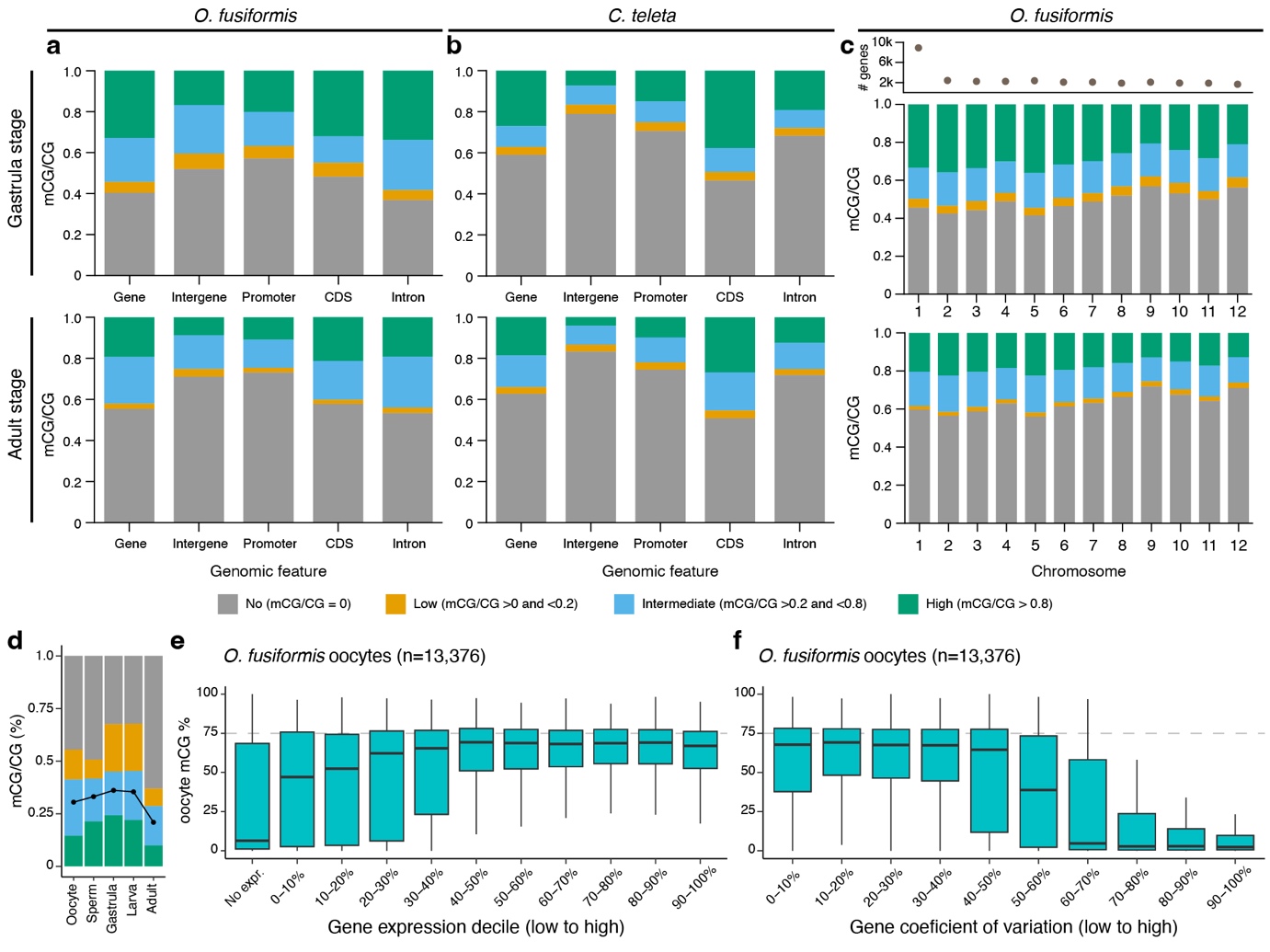


**Fig. S6 – DNA methylation in *O. fusiformis* and *C. teleta* genomes.** (**a**, **b**) Bar plots indicating the proportion of unmethylated CpGs and methylated CpGs with low, intermediate, and high levels according to genomic features at the gastrula and adult stages of *O. fusiformis* (**a**) and *C. teleta* (**b**). (**c**) Bar plots indicating the proportion of unmethylated CpGs and methylated CpGs with low, intermediate, and high levels by chromosome in *O. fusiformis* at the gastrula and adult stages. On top is the number of genes per chromosome. (**d**) Bar plots indicating the genome-wide proportion of unmethylated CpGs and methylated CpGs with low, intermediate, and high levels in oocyte and sperm of *O. fusiformis* compared to gastrula, larval and adult stages. (**e**, **f**) Boxplots depict the correlation between oocyte methylation levels, gene expression (**e**), and gene stability (**f**). As observed in embryonic, larval, and adult stages, 5mC levels positively correlate with gene expression and stability.


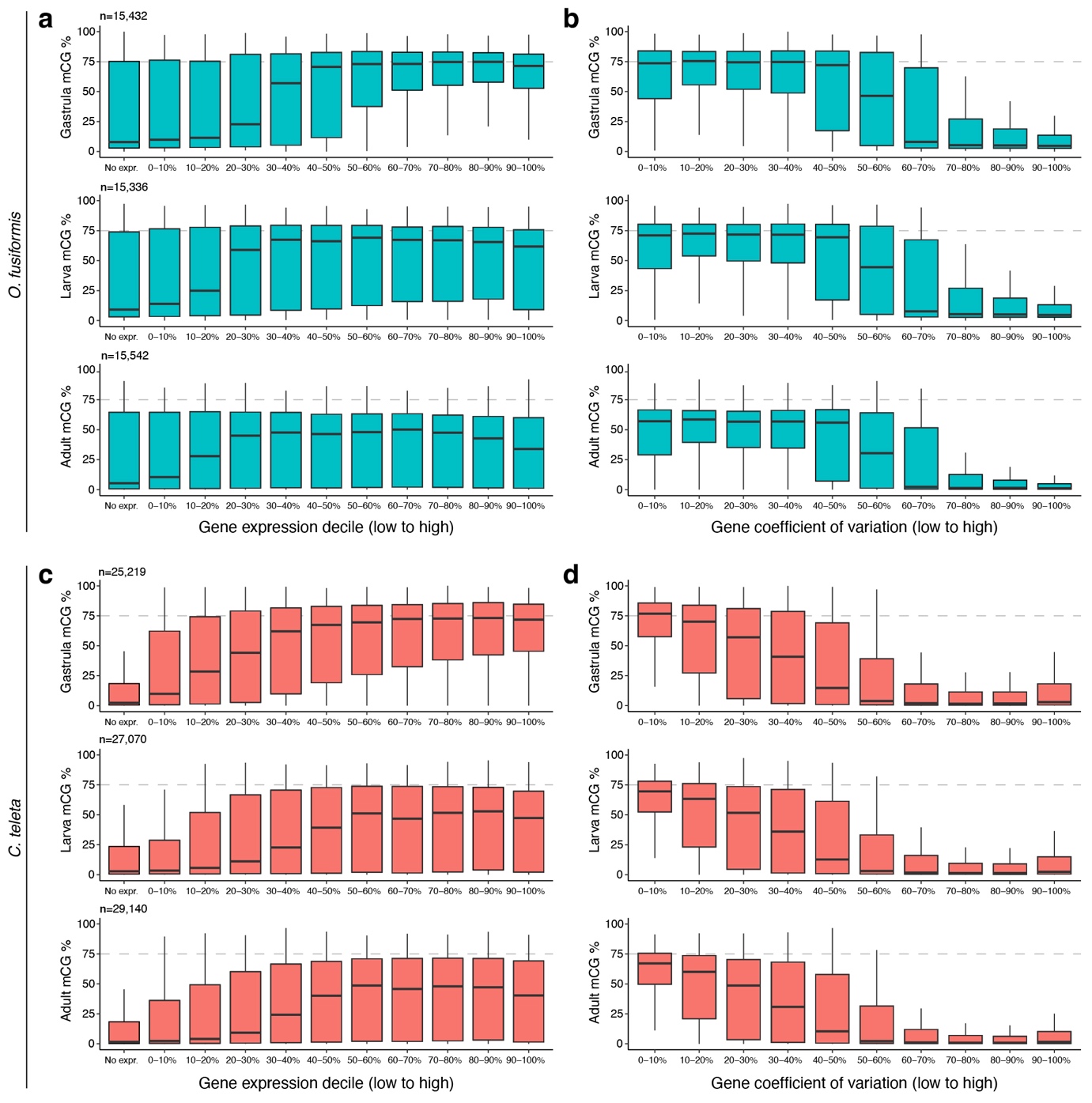


**Fig. S7 – DNA methylation and transcriptional dynamics.** (**a**–**d**) Box plots depicting 5mC levels in gene bodies at gastrula, larval and adult stages of *O. fusiformis* and *C. teleta* according to gene expression (**a**, **c**) and gene coefficient of variation (**b**, **d**) deciles. In both species, highly expressed and more stable genes show higher 5mC levels. Notably, gene body methylation levels of the highest expressed genes decrease as the life cycle progresses in *O. fusiformis* and *C. teleta*.


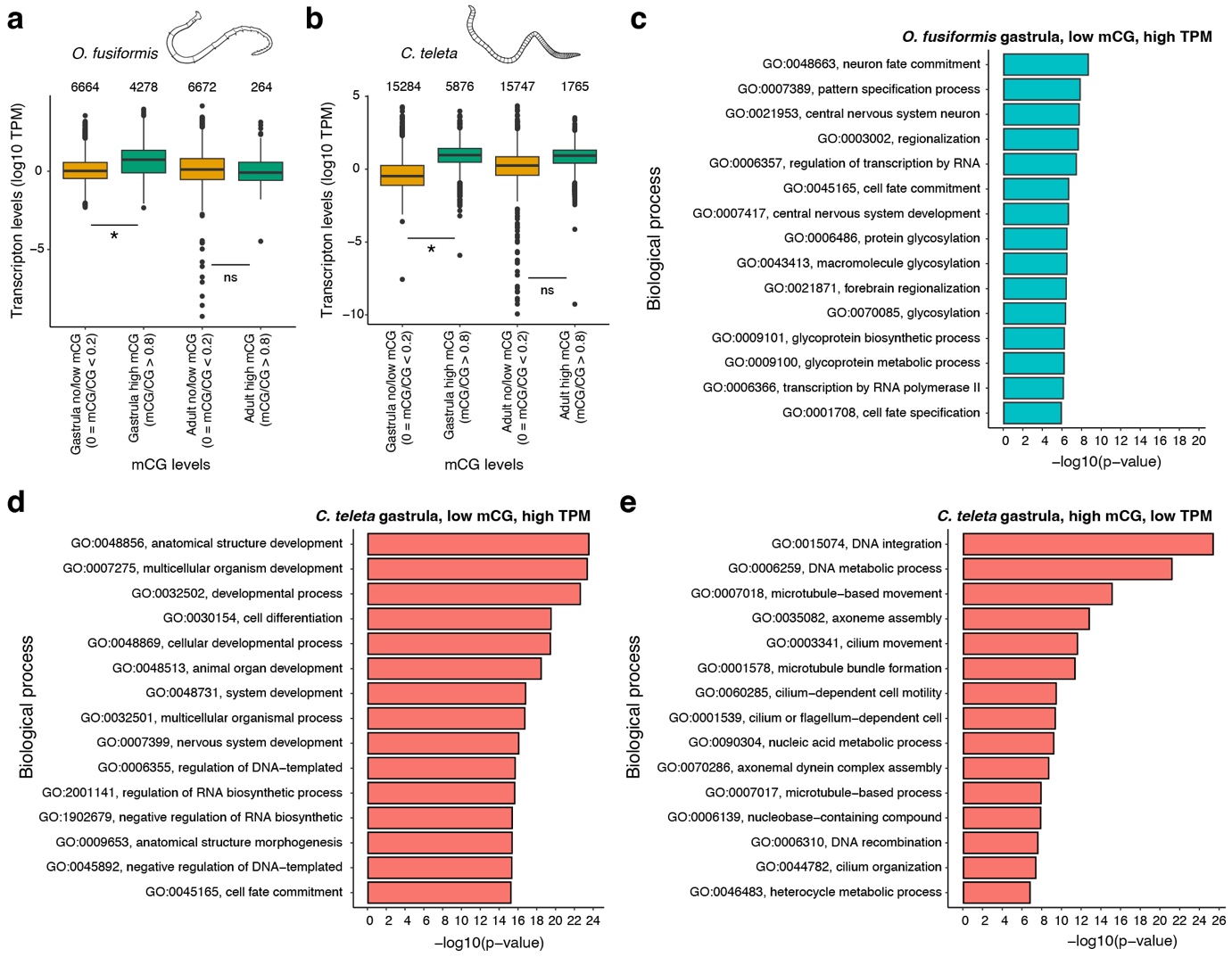


**Fig. S8 – DNA methylation and transcriptional dynamics.** (**a**, **b**) Boxplots depict transcription levels according to no/low and high 5mC gene body methylation levels in *O. fusiformis* (**a**) and *C. teleta* (**b**) at the gastrula and adult stages. Asterisks indicate significance (*p*-value < 0.01; two-tailed t-test). ns, non-significant. (**c**–**e**) Bar plots depicting the top 15 gene ontology (GO) terms for biological processes over-represented in genes whose expression levels are 1.5 times over or under the interquartile difference above the 75th or below the 25th percentile, respectively.


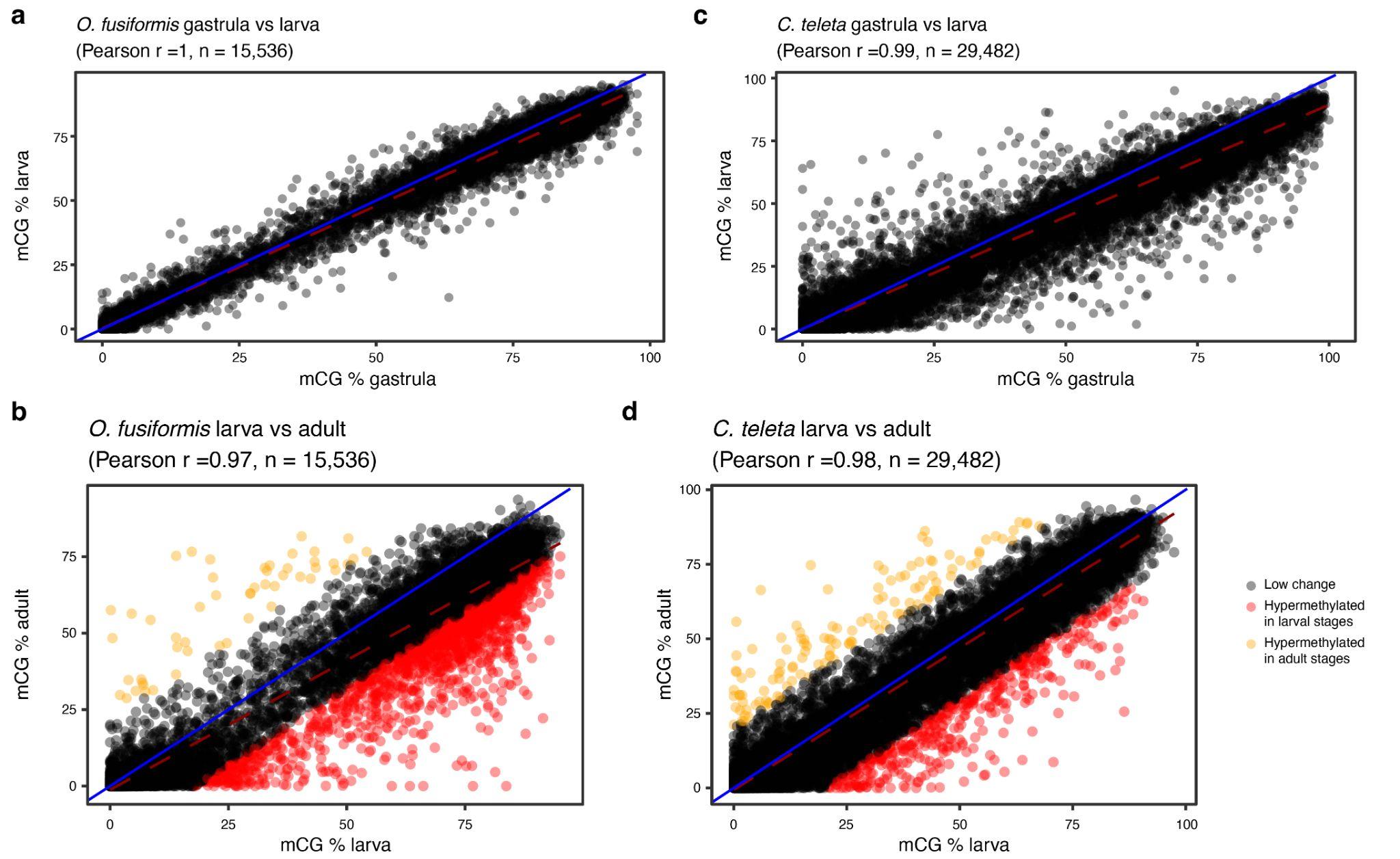


**Fig. S9 – Changes in gene body methylation during the annelid life cycle.** (**a**–**d**) Scatter plots of gene body methylation (GbM) levels between consecutive life stages in *O. fusiformis* (**a**, **b**) and *C. teleta* (**c**, **d**). Genes whose GbM changes more than 20% in their methylation status are coloured in red (hypermethylated in the larval stage) and yellow (hypermethylated in the adult stage). A blue line indicates the theoretic diagonal if values were identical across stages, and the dashed red line indicates the correlation slope.


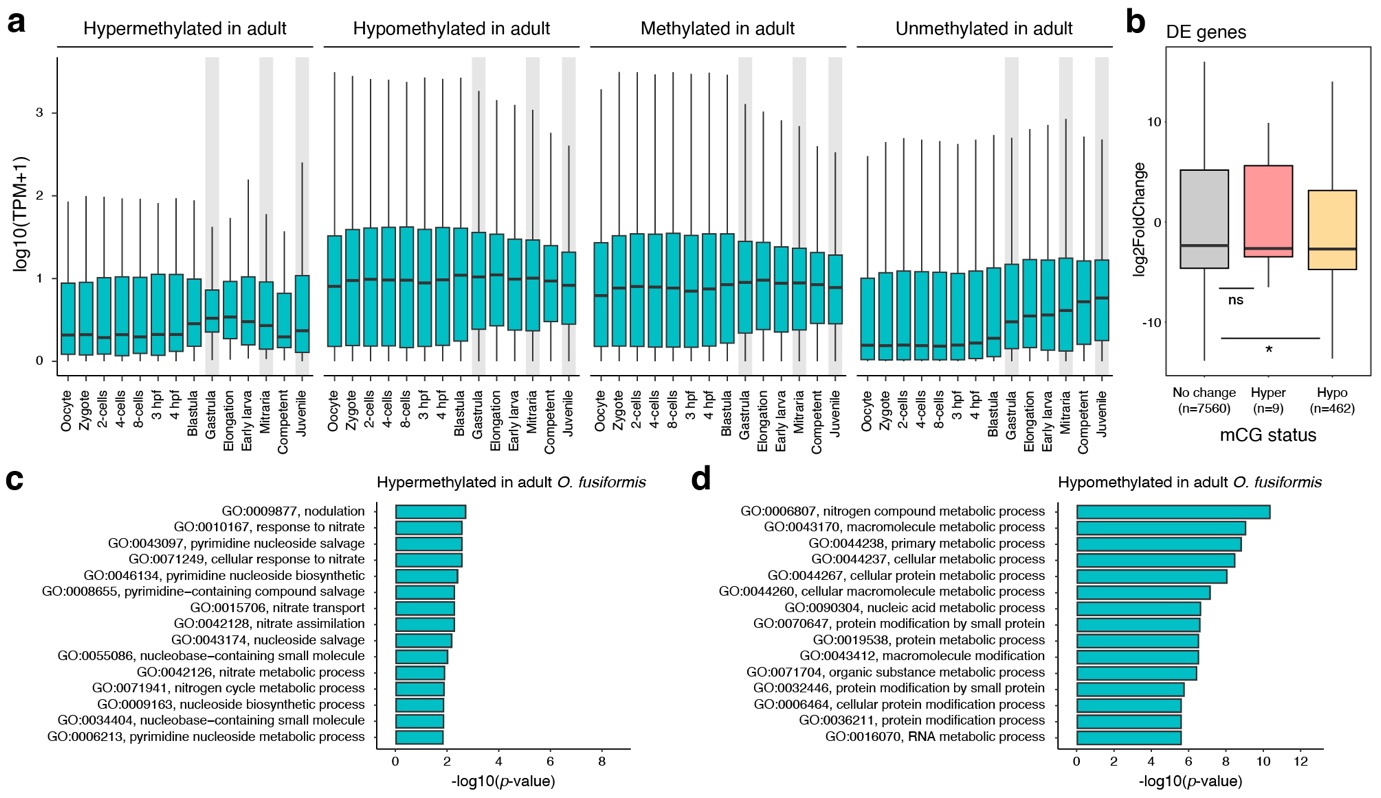


**Fig. S10 – The transcriptional dynamics of genes according to their 5mC levels in *O. fusiformis*.** (**a**) Boxplots of expression levels for genes that are, from left to right, hypermethylated and hypomethylated in the adult compared to the larval stage and methylated and unmethylated in the adult of *O. fusiformis*. Grey bars highlight the stages sampled for genome-wide methylomes. (**b**) Boxplots of fold changes in differential gene expression at the adult stage in genes with no change, hyper-, and hypomethylated methylation status. The asterisk indicates significance (p-value < 0.01, two-tailed t-test). ns, non-significance. (**c**, **d**) Bar plots of Gene Ontology terms enriched in hypermethylated and hypomethylated genes in *O. fusiformis* adults compared to the larval stage.


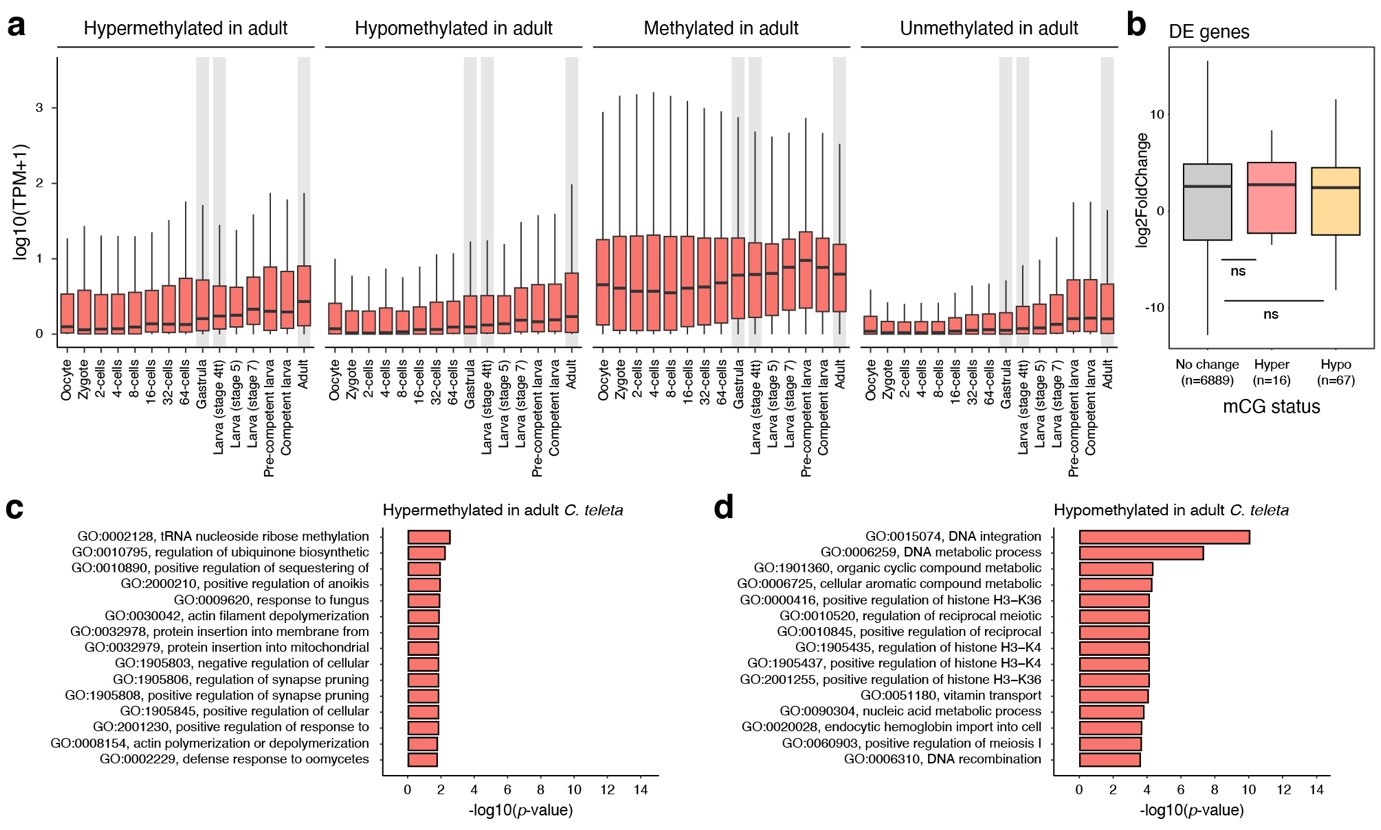


**Fig. S11 – The transcriptional dynamics of genes according to their 5mC levels in *C. teleta*.** (**a**) Box plots of expression levels for genes that are, from left to right, hypermethylated and hypomethylated in the adult compared to the larval stage and methylated and unmethylated in the adult of *C. teleta*. Grey bars highlight the stages sampled for genome-wide methylomes. (**b**) Boxplots of fold changes in differential gene expression at the adult stage in genes with no change, hyper-, and hypomethylated methylation status. ns, non-significance (p-value > 0.01, two-tailed t-test). (**c**, **d**) Bar plots of Gene Ontology terms enriched in hypermethylated and hypomethylated genes in *C. teleta* adults compared to the larval stage.


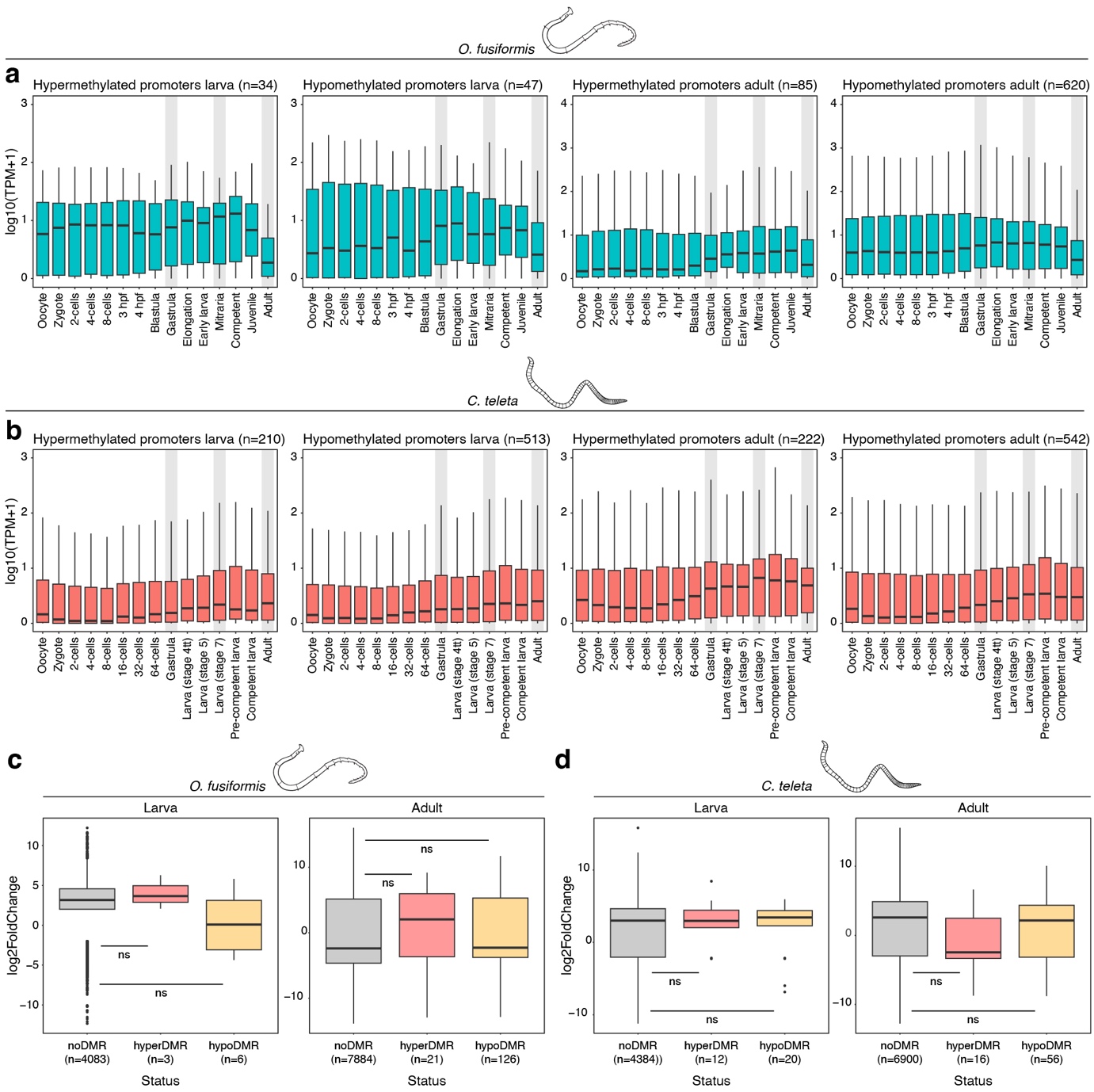


**Fig. S12 – The expression dynamics of genes with promoter DMRs.** (**a**–**b**) Box plots of expression levels from oocyte to adult stages for genes with a hypermethylated (red background) or hypomethylated (yellow background) differentially methylated region (DMR) in their promoter at the larval and adult stages of *O. fusiformis* (**a**) and *C. teleta* (**b**). Grey bars highlight the stages sampled for genome-wide methylomes. (**c**, **d**) Boxplots of fold changes in differential gene expression at larval and adult stages in genes without a promoter DMR, a hypermethylated promoter DMR and a hypomethylated promoter DMR in *O. fusiformis* (**c**) and *C. teleta* (**d**). ns, non-significance (p-value > 0.01, two-tailed t-test).


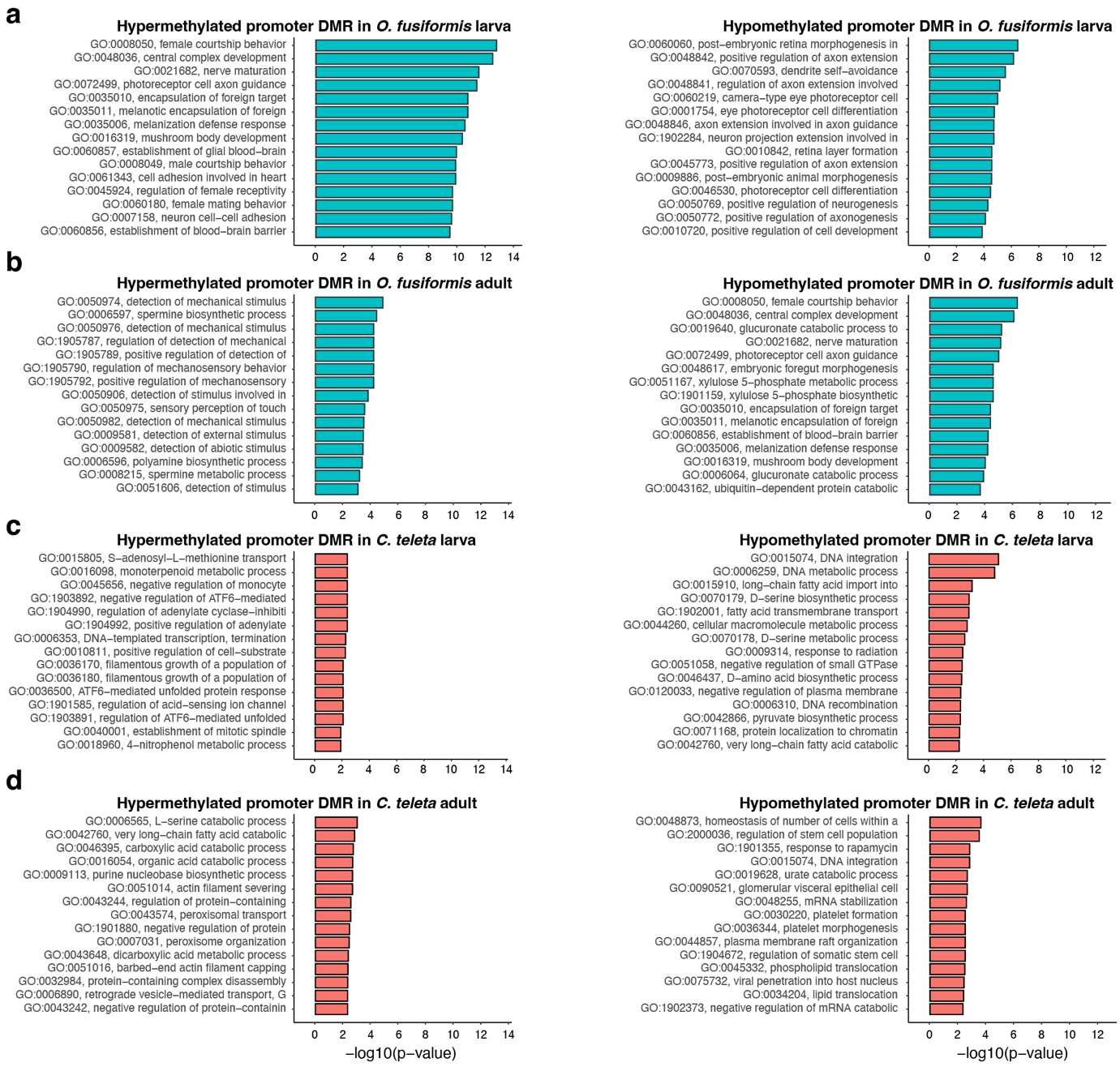


**Fig. S13 – The genes experiencing changes in DNA methylation in their promoters during the life cycle in Annelida.** (**a**–**d**) Bar plots of Gene Ontology terms enriched in genes that exhibit a differential methylated region (DMR) in their promoters in the larva (**a**) and adult (**b**) of *O. fusiformis*, and larva (**c**) and adult (**d**) of *C. teleta*.


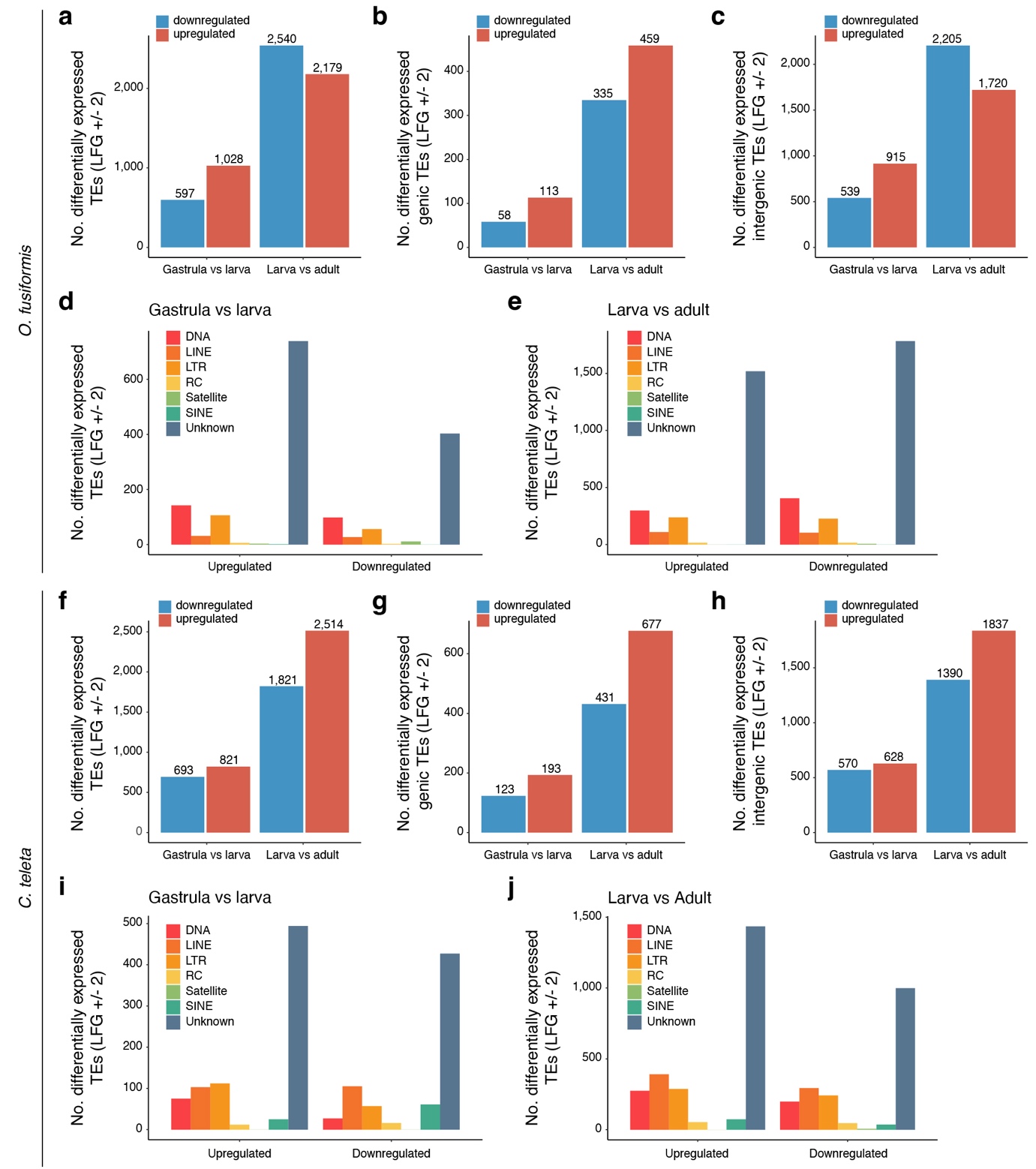


**Fig. S14 – The expression dynamics of transposable elements in *O. fusiformis* and *C. teleta*.** (**a**) Bar plots of upregulated and downregulated transposable elements (TE) between the three consecutive life stages sampled for genome-wide methylomes in *O. fusiformis*. (**b**–**e**) Bar plots of upregulated and downregulated TEs between the three consecutive life stages of *O. fusiformis* depending on whether they are within a gene body (genic; **b**) or not (intergenic; **c**) and the TE class (**d**, **e**). (**f**) Bar plots of upregulated and downregulated transposable elements (TE) between the three consecutive life stages sampled for genome-wide methylomes in *C. teleta*. (**g**–**j**) Bar plots of upregulated and downregulated TEs between the three consecutive life stages of *C. teleta* depending on whether they are within a gene body (genic; **g**) or not (intergenic; **h**) and the TE class (**i**, **j**)


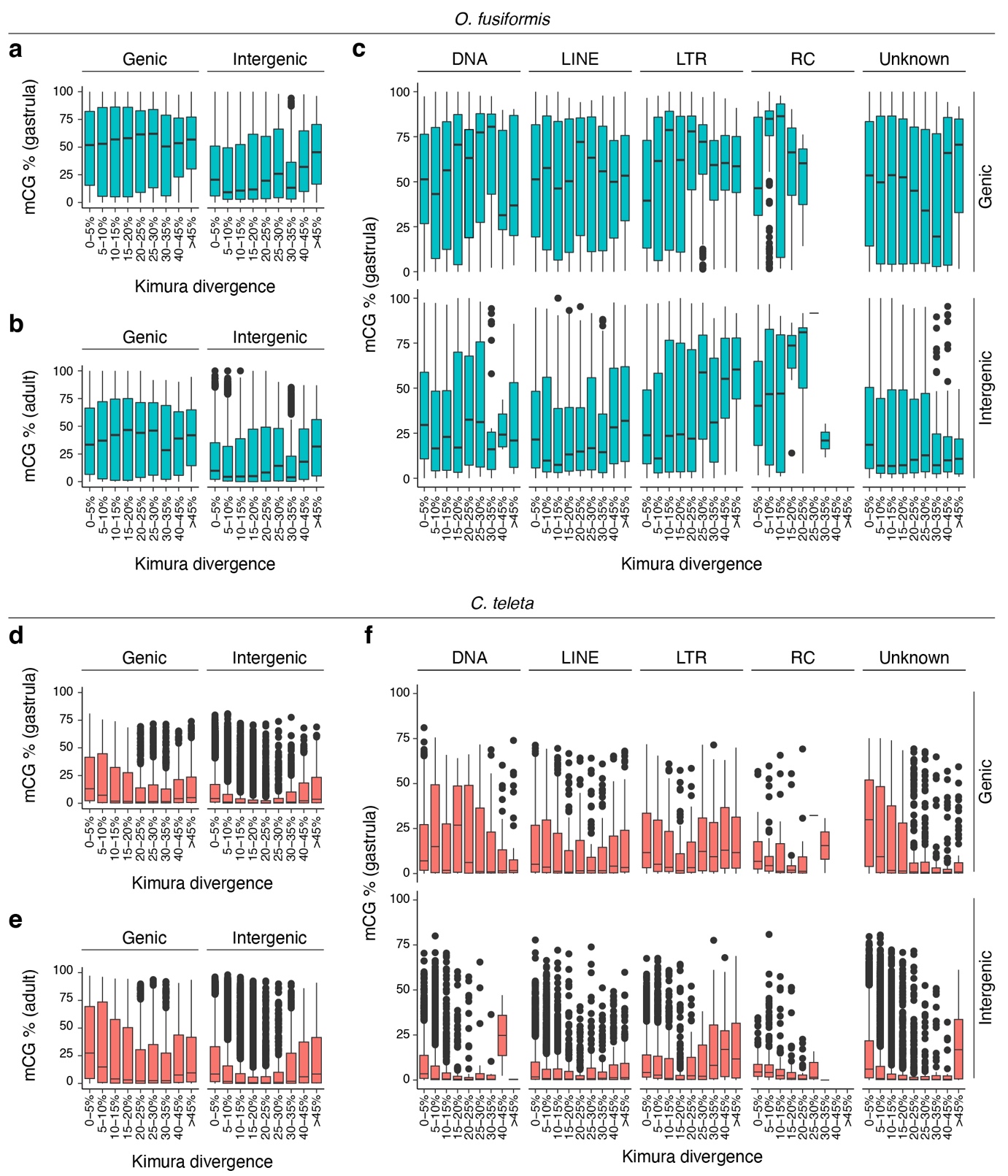


**Fig. S15 – 5mC levels and transposable elements in *O. fusiformis* and *C. teleta*.** (**a**–**f**) Box plots of 5mC levels in transposable elements according to their estimated age from the Kimura divergence value in *O. fusiformis* (green background) and *C. teleta* (red background). In (**a**, **b**, **d**, **e**), transposable elements are subdivided depending on whether they are within a gene body (genic) or not (intergenic). In (**c**) and (**f**), transposable elements are subdivided according to class.

**Fig. S16 – The impact of transposable elements in gene body methylation.** (**a**–**d**) Box plots depicting 5mC levels in gene bodies at gastrula (**a**, **c**) and adult (**b**, **d**) stages of *O. fusiformis* (green background) and *C. teleta* (red background) according to the number of introns (**a**, **c**) and number of introns with a transposable element (TE) (**c**, **d**). (**e**, **f**) Gene body methylation levels according to the number of introns and the presence of an intronic TE in *O. fusiformis* (**e**) and *C. teleta* (**f**). Genes with intronic TEs (yellow) have higher gene body methylation levels than those without intronic TEs (violet) in *O. fusiformis* but not *C. teleta*.

**Fig. S17 – The genes with interspecific differences in gene body methylation in Annelida.** (**a**, **b**) Bar plots of Gene Ontology terms enriched in genes that hypermethylated in *O. fusiformis* compared to *C. teleta* (**a**) and hypermethylated in *C. teleta* compared to *O. fusiformis* (**b**).


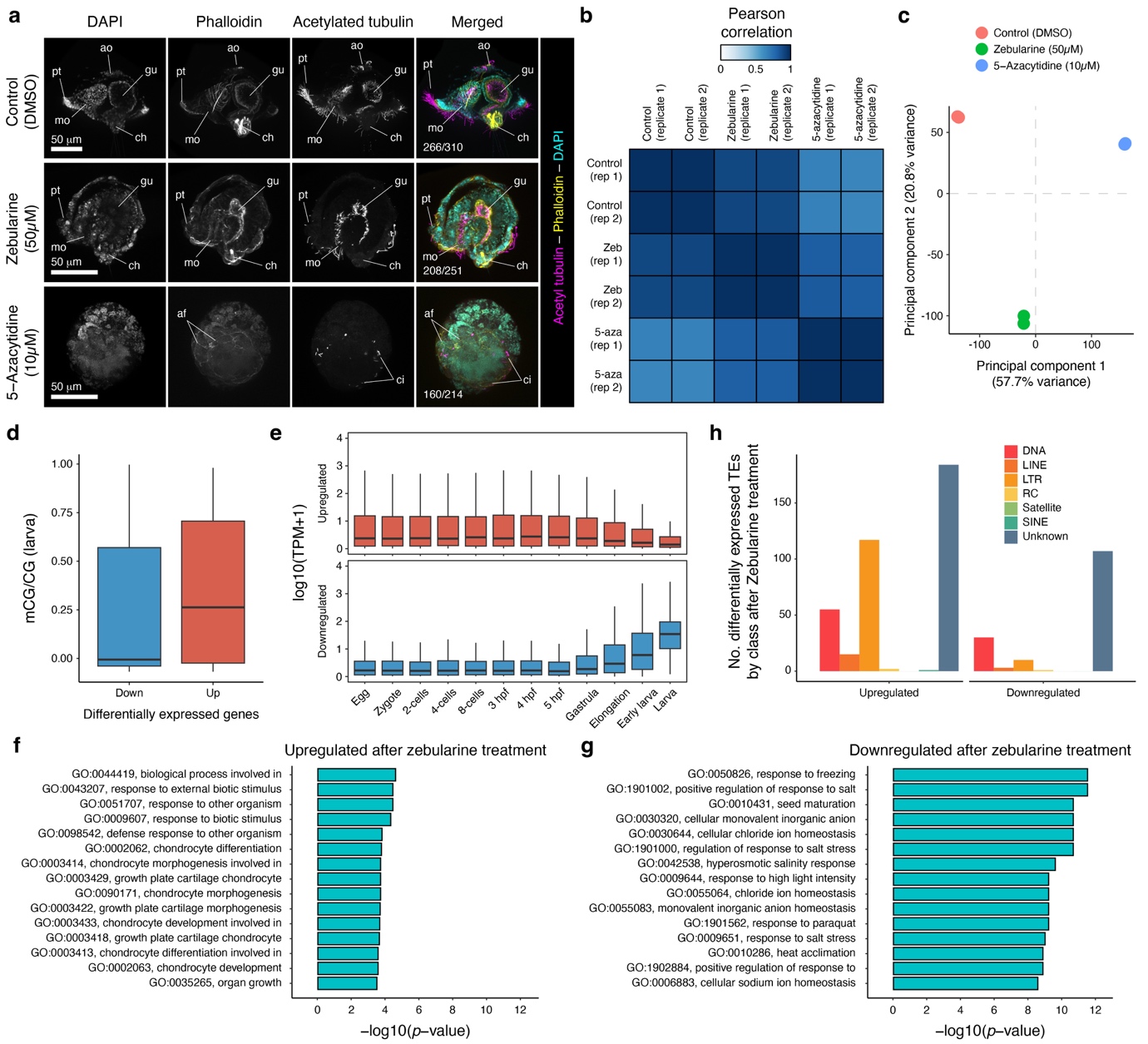


**Fig. S18 – The impact of DNA methylation in the early embryogenesis of *O. fusiformis*.** (**a**) Z-projections of confocal stacks of zebularine- and 5-azacytidine-treated and DMSO-control embryos fixed at the early larval stage and stained for acetylated tubulin (magenta), actin (yellow) and nuclei (cyan). Zebularine-treated embryos fail to undergo normal organogenesis in *O. fusiformis*, while 5-azacytidine prevents gastrulation. (**b**) Heatmap of Pearson correlation coefficients and (**c**) principal component analysis of RNA-seq samples of treated and control conditions. (**d**) Box plot of gene body methylation levels at the larval stage of upregulated and downregulated genes after zebularine treatment. (**e**) Bar plots of differentially expressed transposable elements (TEs) by class after zebularine treatment. (**f**, **g**) Bar plots depicting the enrichment of Gene Ontology terms in the gene set that is upregulated (**f**) and downregulated (**g**) after zebularine treatment in *O. fusiformis*.


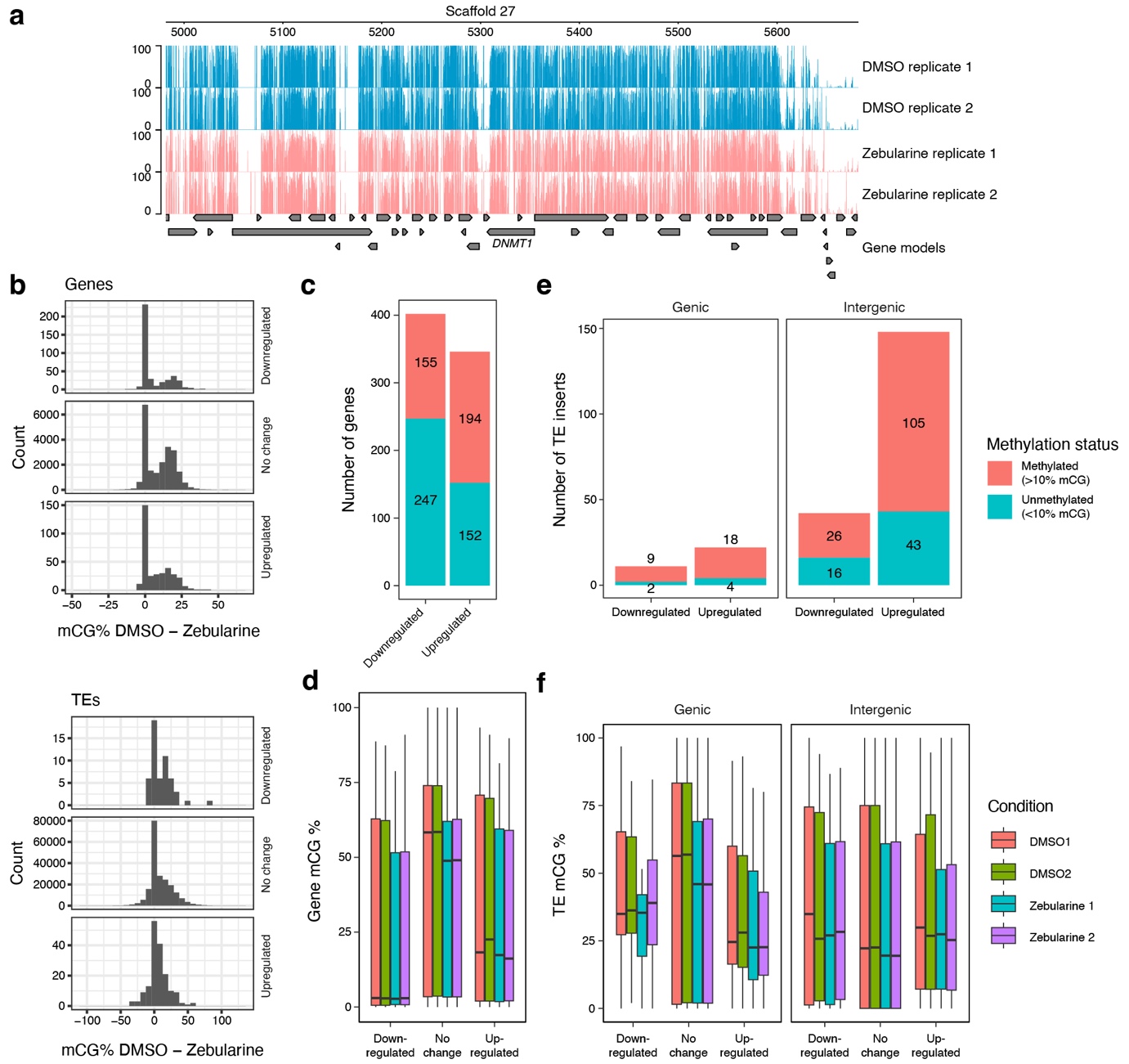


**Fig. S19 – The effect of zebularine in DNA methylation in *O. fusiformis*.** (**a**) Genome browser view showing 5mC levels (binning size 75 bases) around the *DNMT1* locus in *O. fusiformis* comparing DMSO and zebularine treated conditions. Transcriptional units are represented by blue boxes ending in an arrowhead that marks the direction of transcription. Consistent with the estimated 5mC levels, the treated larvae have a modest global demethylation. (**b**) Distribution bar plots of genes (upper) and TEs (bottom) comparing the change in methylation levels between DMSO control and zebularine-treated larvae in downregulated, upregulated and unchanged genomic elements. (**c**, **e**) Bar plots representing the number of genes (**c**) and TEs (**e**) that are methylated or unmethylated in down- and upregulated elements. (**d**, **f**) Box plots representing methylation levels in unchanged, down- and upregulated genes (**d**) and TEs (**f**) in the replicates of DMSO control and zebularine-treated larvae.
