# Supplementary material for: Annelid methylomes reveal ancestral developmental and aging-associated epigenetic erosion across Bilateria
Source: Genome Biol. 2024 Aug 1;25:204. doi: 10.1186/s13059-024-03346-z (PMC11292947; doi:10.1186/s13059-024-03346-z)
Supplement: Supplementary file 2 — Additional file 2: Supplementary Tables. [file 13059_2024_3346_MOESM2_ESM.docx]

**Table S1. Datasets produced in this study.**

| **Species** | **Stage** | **Sequencing type** | **Depth*** | **Replicates** |
| --- | --- | --- | --- | --- |
| *O. fusiformis* | Gastrula | WGBS | 180.2M | 1 |
|  | Larva | WGBS | 159.4M | 1 |
|  | Adult | WGBS | 255.8M | 1 |
|  | Oocyte | EM-seq | 16.4M | 1 |
|  | Sperm | EM-seq | 13.5M | 1 |
|  | 1% DMSO | EM-seq | 9.6M; 10.3M | 2 |
|  | 50 µM Zebularine | EM-seq | 10.1M; 9.1M | 2 |
|  | 10 µM 5-azacytidine | EM-seq | 6.7M; 4.6M | 2 |
|  | Adult | RNA-seq | 22.7–23.4M | 2 |
|  | 1% DMSO | RNA-seq | 31.8–41.3M | 2 |
|  | 50 µM Zebularine | RNA-seq | 30.8–42.5M | 2 |
|  | 10 µM 5-azacytidine | RNA-seq | 35.6–34.8M | 2 |
| *C. teleta* | Gastrula | EM-seq | 71.3M | 1 |
|  | Larva | EM-seq | 75.1M | 1 |
|  | Adult | EM-seq | 69.3M | 1 |
|  | Competent larva | Nanopore | 446–834 | 2 |
|  | Juvenile | Nanopore | 3,115–4,900 | 2 |
|  | Mature female | Nanopore | 2,535–7,696 | 2 |
|  | Senescent female | Nanopore | 23,959–59,749 | 2 |
|  | Adult | RNA-seq | 27–22.3M | 2 |
| *D. gyrociliatus* | Adult | WGBS | 256.5M | 1 |

*Million paired end reads (RNA-seq, EM-seq and WGBS) and number of reads (Nanopore)

**Table S2. Summary of CpG frequency calculations in annelids**

| **Species** | *O. fusiformis* | *C. teleta* | *D. gyrociliatus* |
| --- | --- | --- | --- |
| **CpG dinucleotides** | 8762852 | 9478658 | 1556535 |
| **Cytosine (C)** | 86554427 | 55823136 | 10814336 |
| **Guanine (G)** | 86441988 | 55831156 | 10808663 |
| **Length of genome** | 500139420 | 333283208 | 77897245 |
| **‘N’ nucleotides** | 1020559 | 56624585 | 9614643 |
| **Observed/expected CpGs** | 0.584569 | 0.841396 | 0.909279 |

**Table S3. Number of genes with a hyper- or hypomethylated status and differentially expressed at the adult stage.**

| **Species** | **5mC status** | **Total** | **Differentially expressed** | **Percentage** |
| --- | --- | --- | --- | --- |
| *O. fusiformis* | Hypermethylated | 51 | 9 | 17.65% |
| *O. fusiformis* | Hipomethylated | 1809 | 462 | 25.54% |
| *C. teleta* | Hypermethylated | 125 | 16 | 12.80% |
| *C. teleta* | Hipomethylated | 418 | 67 | 16.03% |

**Table S4. Number of genes with a hyper- or hypomethylated promoter DMR and differentially expressed.**

| **Species** | **5mC status** | **Stage** | **Total** | **Differentially expressed** | **Percentage** |
| --- | --- | --- | --- | --- | --- |
| *O. fusiformis* | Hypermethylated | Larva | 28 | 3 | 10.71% |
| *O. fusiformis* | Hipomethylated | Larva | 42 | 6 | 14.29% |
| *O. fusiformis* | Hypermethylated | Adult | 80 | 21 | 26.25% |
| *O. fusiformis* | Hipomethylated | Adult | 551 | 126 | 22.87% |
| *C. teleta* | Hypermethylated | Larva | 210 | 12 | 5.71% |
| *C. teleta* | Hipomethylated | Larva | 513 | 20 | 3.90% |
| *C. teleta* | Hypermethylated | Adult | 222 | 16 | 7.21% |
| *C. teleta* | Hipomethylated | Adult | 542 | 56 | 10.33% |

**Table S5. List of drug treatments tested in *O. fusiformis*.** This summarises the concentrations tested across two spawning seasons (2020-2021) of *O. fusiformis*. The asterisk (*) mark indicates the first concentration tested.

| Drug treatments | Concentrations tested (μM) | Results |
| --- | --- | --- |
| Zebularine | 5*, 10, 15, 20, 25, 50 | Dose-dependent effect; morphological defect in larval at 25 μM. Strongest phenotype at 50 µM. |
| 5- Azacytidine | 5*, 10, 15, 20, 25 | Dose-dependent effect; arrested at cleavage at 10 μM. |
| Decitabine | 0.1, 1, 2, 5* | Cell death in all concentrations tested. |

**Table S6. Phenotypic penetrance of cytosine analogues in *O. fusiformis*.** This summarises the phenotypes observed after treating *O. fusiformis* embryos with 0.1 µM decitabine, 10 µM 5-azacytidine (5-aza) and 50 µM zebularine (zebu). 1% DMSO was used as a control.

| Replicate | Treatment | No. embryos | Normal | Undeveloped | No gastrulation | Rounded larva |
| --- | --- | --- | --- | --- | --- | --- |
| 1 | DMSO | 301 | 266 | 35 | 0 | 0 |
| 1 | 5-aza | 214 | 0 | 54 | 160 | 0 |
| 1 | zebu | 251 | 0 | 43 | 0 | 208 |
| 2 | DMSO | 222 | 175 | 47 | 0 | 0 |
| 2 | 5-aza | 261 | 0 | 56 | 205 | 0 |
| 2 | zebu | 195 | 0 | 84 | 0 | 111 |
| 3 | DMSO | 210 | 150 | 60 | 0 | 0 |
| 3 | 5-aza | 353 | 0 | 39 | 314 | 0 |
| 3 | zebu | 309 | 0 | 67 | 0 | 242 |

**Table S7. Methylation and non-conversion rate values after cytosine analogue treatments.** Global methylation levels after treating *O. fusiformis* embryos with 10 µM 5-azacytidine (5-aza) and 50 µM zebularine (zebu). 1% DMSO was used as a control.

| Replicate | Treatment | mCG | λ mCG |
| --- | --- | --- | --- |
| 1 | DMSO | 31.246 | 0.214 |
| 2 | DMSO | 31.832 | 0.118 |
| 1 | zebu | 26.533 | 1.073 |
| 2 | zebu | 27.251 | 0.332 |
| 1 | 5-aza | 31.227 | 0.163 |
| 2 | 5-aza | 30.904 | 0.233 |
