# Supplementary material for: Annelid methylomes reveal ancestral developmental and aging-associated epigenetic erosion across Bilateria
Source: Genome Biol. 2024 Aug 1;25:204. doi: 10.1186/s13059-024-03346-z (PMC11292947; doi:10.1186/s13059-024-03346-z)
Supplement: Supplementary file 3 — Additional file 3. Review history. [file 13059_2024_3346_MOESM3_ESM.docx]

Review History

**First round of review**

**Reviewer 1**

**Were you able to assess all statistics in the manuscript, including the appropriateness of statistical tests used?**

Yes, and I have assessed the statistics in my report.

**Comments to author:**

By using base pair resolution genome-wide profiling approaches, Guynes et al. investigated the 5mC levels in three annelid species, including Owenia fusiformis, Dimorphilus gyrociliatus and Capitella teleta, during development. This study reveals that O. fusiformis and C. teleta display moderate 5mC levels and a typical mosaic pattern, but D. gyrociliatus shows negligible methylation as adults. DNA methylation positively correlates with transcriptional levels and stability, and normal methylation levels are essential for successful embryogenesis in O. fusiformis. They also reveal that the global DNA methylomes erode along development and aging in these annelids. It provides a valuable resource to investigate the function of DNA methylation in annelid species. However, some major concerns need to be addressed.

1.The authors mainly concern the relationship between GbM and gene expression. Actually, the methylation state of promoter is also an important mechanism regulating gene expression. However, the results of differentially methylated promoters during development are not provided. Are the differentially methylated promoters associated with the differentially expressed genes during development in O. fusiformis and C. teleta?

2.For genes with hypomethylated or hypermethylated GbM at adult stage for each species, are there any differentially expressed genes when comparing the expression level of each gene between larval and adult stages? How many genes with differentially methylated gene bodies showing differential expression between larval and adult stages?

3.For Nanopore methylomes at four ages in C. teleta, how many DMRs can be detected between adjacent stages, especially between adult stage and senescent stage? How about the genomic distribution of these DMRs? What are the functions of the DMR-associated genes?

4.The authors mentioned that treatment with 10uM 5-azacytidine either killed the embryo (25.23%) or resulted in abnormal cleavage and gastrulation failure. It seems that the concentration of 5-azacytidine used for embryo treatment is too high. How was the concentration of 5-azacytidine used for embryo treatment determined? Whether the treatment with lower concentrations of 5-azacytidine were performed?

5.The DMRs are not analyzed between the embryos treated with zebularine or 5-azacytidine and the control embryos? Is there any relationship between the DMRs and abnormally expressed genes or TEs?

6. The authors should explain why some EM-seq or WGBS assays have only one replicate?

Minor Points:

1.In C. teleta, only DNMT1b has a zf_CXXC domain, whereas DNMT1a does not have zf_CXXC domain (Fig. S1a). Thus, the labels in Fig.1c for C.teleta DNMT1 are not correct.

2.In human genome, there are 3 TET genes (TET1, TET2 and TET3). Does TET1 and TET3 orthologous genes in annelids?

3.Scale bars are missing in some panels, such as Fig. 1e, Fig. S5b etc.

4.In Fig. 2i, the results of statistical tests are not shown. The numbers of genes in each group are also not provided. Fig. 3c,d,f have the same problems.

5.In Page 12 Lines 20-24, they claimed that "Transcriptionally, genes with decreased GbM in the adult behave like the other methylated genes whose methylation status". However, the results are not shown. Some heat maps can be provided.

**Reviewer 2**

**Were you able to assess all statistics in the manuscript, including the appropriateness of statistical tests used?**

No, I do not feel adequately qualified to assess the statistics.

**Comments to author:**

In this manuscript, Guynes et al describe computational and functional studies to investigate the DNA methylation landscape and correlation with gene and transposon expression in 3 species of annelid worms. This is valuable as this clade is understudied in the epigenomics field and annelids represent a huge variety of animals. The use of genomics and functional studies in this work elevates it to one that is relevant to epigeneticists, genomicists and developmental biologists. An important aspect of this study is that it addresses a a dogma in the field that there is a direct correlation between gene body methylation and expression in invertebrates. While this work makes some novel findings about this correlation (or lack there of) more detailed analysis are required to solidify this important advance.

Using a range of methods to assess cytosine methylation, the authors find that one species of annelids has virtually no DNA methylation. In contrast, 2 others have a low level of DNA methylation that is distributed across the genome in what is considered a stereotypic pattern for many invertebrates: concentrated on a subset of gene bodies and introns, and absent from promoters and transposons. They investigate this pattern across developmental stages and in adult animals, including senescent females, which is aimed at determining whether DNA methylation patterns show an age-associated randomization as found in other species (mostly vertebrates). Previous studies have suggested that the function of DNA methylation in species with low methylation (like 2 of the species studied here, O. fusiformis and C. teleta) is to maintain the expression of housekeeping or highly expressed genes. The authors examine this and find little to no support for this hypothesis. Finally, to functionally test the requirement for DNA methylation in O. fusiformis by treating larvae with DNA methyltransferase inhibitors, revealing some defects in development and changes in gene and TE expression.

Overall, this is an interesting study that is both broad and deep. The data is presented clearly and the manuscript is well written. There are several features of this work that are interesting and novel, including the comparison of 3 species in the same clade with divergent DNA methylation patterns, the convincing evidence that gene body methylation is not a likely mechanism regulating gene expression and the intriguing idea that DNA methylation changes during aging in at least one species. Additionally, the inclusion of experimental data is a strength of this study. While these are all positive aspects of this study, there are a number of areas that would benefit from attention, including some controls for the experimental data, a tissue specific analysis of the methylation and gene expression patterns and expanding on some of the details, which can involve bringing some of the materials from the supplement into the main figures.

Major

1. The major issue that would benefit this paper is using tissue specific methylome/transcriptome to address the correlation between gene body methylation and gene expression in these animals. All of the studies are performed with cells from a wide range of lineages, making it nearly impossible to draw correlations between how and where a gene is expressed and the state of it's methylation. Unless they show that the is a static methylation pattern in all cells, as has been reported in vertebrates, one of the main points of this paper is supported by data that is not clear.

2. The intermediate methylation data can confound the correlation data. If only the high (green bars in figure 2A-B) and low/no methylation (orange and grey bars in Figure 2A-B) are separated, what is the correlation with expression? This is addressed only partially in Figure 2C-D, and it looks like only those with very high methylation are highly expressed; it would be interesting to look at this in more detail to see what are the genes that correlate with this pattern and what do not.

3. One of the most important findings in the paper is the functional validation of the importantce of DNA methylation in annealid development. In Fig.5D, zebularine treatment of O. fusiformis results in expression of TEs from intergenic regions, but the analysis . However, DNA methylation values on TEs after treatment are missing and should be included.

4. Figure 1 would benefit from presenting data that gives an overview of these species. Figure 1b could be adjusted to not only include TE abundance in these genomes, but could also include the other genomic elements (promoters, exons, introns etc) to give a sense of how these genomes are organized. ]

5. Similarly, an overview of where 5mC is distributed in each genome would be of interest.

6. Data in figure 1 and S5 show that there is a spatiotemporal pattern of the DNMTs, MBDs and TETs during development. The interpretation of this data is cursory. If these genes are patterning the methylome, which is present in all cells, then how is it that not all cells express them? Instead, if they are functioning during only discrete developmental events, then how is the methylome in the rest of the embryo getting set? Finally, Dnmt1 typically shows an expression pattern that mirrors cells in S-phase. Can that explain the discrete pattern of expression seen in late embryos and larvae? Some additional staining with other genes - such as Uhrf1 and Pcna - would help clarify this.

7. THe data on TEs would benefit from some more detailed investigation. The pie charts in Figure 1 shows transposable elements (TEs) but in the caption they are referred as repetitive elements. This is an impotant distinction. It would be nice to have these elements fully annotated and correlated with

Minor

1. Uhrf1 is missing from the comparison of conservation of methylation machinery in figure 1C

2. Given that TETs lack the key residues for cytosine demethylation in the species investigated, it is important to comment on whether the TETs in the species studied here are predicted to function in DNA methylation or if it has assumed a different function.

3. The data in S5 would be useful to have in main Figure 1, as it gives a broad overview of what is happening when during development of these animals. THe in situs are valuable data.

4. The data in Figure S6 is really nice and would benefit from being in the main figure.

5. One interesting finding in Fig.S4 and S5 is that C. teleta has two DNMT1s, with one missing CXXC domain (DNMT1a) while the other missing RFD domain (DNMT1b). Since Fig. S5 showed that DNMT1b has an expression profile similar to UHRF1, perhaps this is the DNA methylation maintenance enzyme in this organism while DNMT1a may have another other role. It seems that C. teleta has split different roles of DNMT1 in two distinct genes. Could the authors comment on this and report if to their knowledge there are other organisms that invoke such strategy in their genomes?

6. The phenotypic differences in the embryos treated with zebularine and 5-azacytodine are very clear, but it is not clear if all embryos have this response or if it is only some, and if it is only some, which were sequenced? A simple scoring of phenotypic penetrance would be helpful.

7. In Fig 1c, the crossed-circle for 'zf-CXXC domain loss' is missing in one of the paralogs of DNMT1 in C. teleta. Also, a 'visual code' for missing RFD domain in DNMT1.

8. In Fig.3c-d, the color coding of Hyper-mCG in O. fusiformis in box plot could be more divergent to the color used for C. teleta to make the graph more readable.

9. In Fig.S14, it seems that panels are missing about adult or gastrula respectively in each animal. In O. fusiformis box plots of TEs divided by class are presented only for gastrula and not for adult, while for C. teleta only adult panel are reported and gastrula data are missing.

**Reviewer 3**

**Were you able to assess all statistics in the manuscript, including the appropriateness of statistical tests used?**

No, I do not feel adequately qualified to assess the statistics.

**Comments to author:**

This study is important, as it contributes vital knowledge about the highly evolutionarily dynamic patterns of methylation among animal species. Base-resolution studies such as these are crucial for the field to move forward. There are many nice observations and important results, however there are major flaws that need to be addressed and clarified.

Major concerns:

The functional analysis is of most concern, as only base-analogs were used to disrupt methylation, and this approach has many caveats that make results hard to interpret in any model system (see review Seelan et al., 2018 DOI: 10.1080/03602532.2018.1437446

). In the present case only one of the drugs was shown to have a potentially specific effect, as the azacytidine and decitabine appeared to be toxic without affecting DNA methylation. The reason why no changes in DNA methylation were observed despite obvious effects of the drugs is not explored, and is concerning. For the putatively functional zebulanine, the reduction of methylation was apparently quite modest in magnitude. Given that the other drugs give massive morphological phenotypes and presumably major alterations in gene expression patterns, despite no changes in methylation. It is entirely possible that the mild gene expression effects and phenotypes are due simply to a milder toxicity and are not related the minor reduction in methylation levels. The authors do not address this possibility, but it is something that needs to be falsified in order for their claims about the functions of methylation to be credible (especially in the discussion).

Missing from the methods is how many replicates were done for both the EM-seq and RNA-seq after drug treatment, this can also affect the credibility of the results and should be included. Finally, it appears that CpG conversion in lambda DNA was noticeably less efficient in the zebulanine experiment. The exact magnitude is not clear by eye in the figure, but the authors should give an indication of the expected increase false negative methylation calls in the whole genome sample based on the rate in the phage DNA. Would correcting for this evaporate the statistical significance of the modest methylation reduction observed.

One possibility to partially address these concerns could be to correlate genes with changed expression levels, with observed changes in methylation. It seems that they did genome wide methylation sequencing for the drug treated samples, so this should be possible. If methylation was important, there should be some corrlelation of changes in gene expression, and changes in methylation at the particular locus.

Similarly, the results with TE's after drug treatment needs more support. Activation of TE's is common result of stress, so it would not be suprising to see them activated after a toxic treatment. Consistent with TE activation not being related to changes in methylation, the authors state that the upregulated TEs are almost exclusively intergenic. However, earlier they show that it is the intragenic TE's that are associated with high levels of methylation. One would therefore expect the intragenic TE's to be more susceptible to changes in methylation, and explanation is needed why this is not the case.

Minor points:

The acronym EM-seq should be defined on first use It would be worthwhile to discuss the advantages/disadvantages/interchangeability of this method vs WGBS.

In the figure legend, figure 1f is described out of order, and should follow the alphabetic order in the figure.

**Authors Response**

**Point-by-point responses to the reviewers’ comments:**

Reviewer #1:

By using base pair resolution genome-wide profiling approaches, Guynes et al. investigated the 5mC levels in three annelid species, including Owenia fusiformis, Dimorphilus gyrociliatus and Capitella teleta, during development. This study reveals that O. fusiformis and C. teleta display moderate 5mC levels and a typical mosaic pattern, but D. gyrociliatus shows negligible methylation as adults. DNA methylation positively correlates with transcriptional levels and stability, and normal methylation levels are essential for successful embryogenesis in O. fusiformis. They also reveal that the global DNA methylomes erode along development and aging in these annelids. It provides a valuable resource to investigate the function of DNA methylation in annelid species. However, some major concerns need to be addressed.

1. The authors mainly concern the relationship between GbM and gene expression. Actually, the methylation state of promoter is also an important mechanism regulating gene expression. However, the results of differentially methylated promoters during development are not provided. Are the differentially methylated promoters associated with the differentially expressed genes during development in O. fusiformis and C. teleta?

*RESPONSE: To explore the relationship between differential methylation in promoters and gene expression, we have plotted the developmental expression dynamics of the genes with a promoter DMR (Supplementary Figure 12a, b) and calculated the number of genes with a promoter DMR that shows a significantly different expression change (log fold change > or < 2; Supplementary Table S4). However, differentially expressed genes with a promoter DMR are only a small fraction of all differentially expressed genes in larvae and adults and do not show significantly different expression levels than the other differentially expressed genes (Supplementary Figure 12c, d). We included these new findings in the main text (lines 292–296).*

2. For genes with hypomethylated or hypermethylated GbM at adult stage for each species, are there any differentially expressed genes when comparing the expression level of each gene between larval and adult stages? How many genes with differentially methylated gene bodies showing differential expression between larval and adult stages?

*RESPONSE: Less than 25% of the genes with hyper- or hypomethylated status in the adult are differentially expressed (Supplementary Table S3). Notably, genes whose methylation status changes in the adult show a similar distribution of fold changes in gene expression versus the larval stage compared to genes without changes in gene body methylation (Supplementary Figures 10b and 11b). We refer to these findings in the main text (lines 265–269).*

3. For Nanopore methylomes at four ages in C. teleta, how many DMRs can be detected between adjacent stages, especially between adult stage and senescent stage? How about the genomic distribution of these DMRs? What are the functions of the DMR-associated genes?

*RESPONSE: These are low-coverage methylomes (line 374–375 and Supplementary Table S1) that are enough to measure global methylation levels rather than high-coverage base-resolution changes in the methylation landscape with age. Still, Nanopore global methylation levels reproduce those of EM-seq, despite being based on totally different principles (enzymatic conversion of unmethylated cytosine vs direct modification detection). Given the length of this experiment extends beyond the 8-week timeline for submitting the revisions (~5.5 months from egg deposition to senescent adult) and the comment of reviewer #2 asking to minimise this part of our work, we have not repeated this analysis using another technique. We also do not have the original libraries to re-sequence because the library preparation method of multiplexed sequencing with Nanopore uses limited amounts of input DNA (compared to a typical Nanopore ligation method), and we sequenced the whole library until all pores of the instrument allowed. However, we now indicate that high-coverage methylomes are warranted to discern whether the de-methylation concentrates in specific regions or is a more general phenomenon and how it might impact transcriptional dynamics during senescence (lines 386–388: “In the future, deep-coverage methylomes will show whether this age-dependent demethylation concentrates in specific genomic regions and its correlation with changes in transcriptional dynamics with age in this annelid.”).*

4. The authors mentioned that treatment with 10uM 5-azacytidine either killed the embryo (25.23%) or resulted in abnormal cleavage and gastrulation failure. It seems that the concentration of 5-azacytidine used for embryo treatment is too high. How was the concentration of 5-azacytidine used for embryo treatment determined? Whether the treatment with lower concentrations of 5-azacytidine were performed?

*RESPONSE: As shown in Supplementary Table S3, the first concentration tested for 5-azacytidine was 5 µM, mirroring the zebularine treatment. These concentrations were devised based on published treatments in other invertebrates and chemical treatments during embryogenesis in O. fusiformis (e.g., DOIs: https://doi.org/10.1186/s12915-021-01074-5 and https://doi.org/10.1038/s41467-022-30004-4). For 5-azacytidine, we did not observe any phenotype at the lowest concentration, with the phenotypes appearing at 10 µM, which also agrees with results in the oyster Crassostrea gigas (DOI: 10.1007/s10126-013-9523-2). This is consistent with a gradual transition from no effect (5 µM) to toxicity, with arrested cleavage at 10 µM being a mild toxic effect (inferred by the lack of methylation depletion), and with the action mode of these drugs (cytosine deaminase inactivates most cytidine analogues, hence higher concentrations are needed to produce an effect, whilst zebularine also inhibits cytidine deaminases and therefore is more effective at lower concentrations).*

5. The DMRs are not analyzed between the embryos treated with zebularine or 5-azacytidine and the control embryos? Is there any relationship between the DMRs and abnormally expressed genes or TEs?

*RESPONSE: Our original EM-seq experiment was performed to low coverage just to determine global methylation levels between treatments and control. However, following the reviewer’s suggestion, we have sequenced those libraries to a deeper coverage (aiming at ~20x per library) to look for DMRs. Since we have replicates, we used dmrseq, a powerful method that provides statistics on the DMRs. Unsurprisingly, we obtained no DMRs that passed the FDR correction threshold (0.05). We manually inspected the best DMRs, which had “significant” p-values (<0.01) that, upon multiple test corrections, were not significant on the genome browser, and indeed, neither of them looked convincing (few CpGs involved, noisy coverage). This is not surprising given that the mechanism of action of zebularine (or any of the cytidine analogues) depends on sequestering DNMTs when bound to DNA, leading to their degradation. This, in turn, results in a global, genome-wide stochastic loss of 5mC. 5-azacytidine or zebularine should not, in principle, be sequence-specific or lead to loss on some regions more than others, as loss of maintenance and de novo methyltransferase activity will act independently on every cell. A global reduction of 4.2% suggests that the 5mC loss occurs in a subset of cells or that in each cell, it has a limited effect and likely a low incorporation rate. Indeed, by inspecting the genome browser, 5mC patterns of zebularine recapitulate those of DMSO controls, but with a globally reduced level at most CG sites (new Supplementary Figure S19).*

*Although DMR analysis did not provide regions statistically more demethylated than others, we focused on the TEs and genes transcriptionally de-regulated on zebularine-treated samples. We observe a mild depletion of methylation on those genes, yet again this is the result of modest global reduction (~4% mCG), which implies that it is not most of the cells in the larva that will have those regions demethylated, but perhaps some stronger methyl-sensitivity that can be picked up at the bulk level or a generally low incorporation rate. We have now included new figures about these results in Supplementary Figure 19. We also observe that several originally unmethylated genes (247 downregulated and 152 upregulated) and TEs (18 downregulated and 47 upregulated) are also dysregulated in Owenia, which suggests some level of indirect drug effects on gene expression. This might be a downstream regulatory cascade effect of other genes or responses to a potential mild toxicity of the drug (as suggested by reviewer #3). However, most TEs that are upregulated upon zebularine treatment were originally methylated (72%), suggesting that some of the upregulation might be associated with 5mC loss. We discuss these results and nuanced interpretations of the drug treatments in the main text (lines 413–438 and 546–578).*

6. The authors should explain why some EM-seq or WGBS assays have only one replicate?

*RESPONSE: The EM-seq and WGBS samples of the different annelid life stages are unreplicated deep-sequenced methylomes to obtain high-coverage base-resolution information. Importantly, the global methylation levels inferred from them are consistent with those derived from replicated methylomes obtained for other analyses. For example, the EM-seq methylation levels in the larvae and adults of C. teleta (Figure 4b) are consistent with those from Nanopore data (Figure 6e). Likewise, the larval methylation levels in O. fusiformis obtained through WGBS (Figure 4a) are comparable to those of DMSO-control larvae obtained with EM-seq (Figure 7c). We now include a sentence in the Methods highlighting that although some of our samples are unreplicated and/or produced with different approaches, the whole characterisation of the methylomes of C. teleta and O. fusiformis is internally consistent and follows congruent developmental trajectories (lines 668–671). During the development of the project, the usage of WGBS was substituted by EM-seq due to lower input material requirements and reported lower coverage bias of this technique, allowing easier obtention of replicates, especially at observations based on low-coverage sequencing essays (Nanopore / original EM-seq drug treatment screenings).*

Minor Points:

1. In C. teleta, only DNMT1b has a zf_CXXC domain, whereas DNMT1a does not have zf_CXXC domain (Fig. S1a). Thus, the labels in Fig.1c for C.teleta DNMT1 are not correct.

*RESPONSE: Corrected.*

2. In human genome, there are 3 TET genes (TET1, TET2 and TET3). Does TET1 and TET3 orthologous genes in annelids?

*RESPONSE: As Supplementary Figure 3 suggests, the multiple TET copies in humans are likely the result of vertebrate-specific expansions. Most annelids show only one TET gene, as many other invertebrates, and their domain composition is comparable to TET2 in humans (Supplementary Figure 4).*

3. Scale bars are missing in some panels, such as Fig. 1e, Fig. S5b etc.

*RESPONSE: Scale bars have been added to the former Figure S5 (now Figure 2) and any new figure.*

4. In Fig. 2i, the results of statistical tests are not shown. The numbers of genes in each group are also not provided. Fig. 3c,d,f have the same problems.

*RESPONSE: We have added the number of genes in each group in those figure panels and others and performed statistical tests.*

5. In Page 12 Lines 20-24, they claimed that "Transcriptionally, genes with decreased GbM in the adult behave like the other methylated genes whose methylation status". However, the results are not shown. Some heat maps can be provided.

*RESPONSE: Amended. This sentence referred to the former Supplementary Figure 9a.*

Reviewer #2:

In this manuscript, Guynes et al. describe computational and functional studies to investigate the DNA methylation landscape and its correlation with gene and transposon expression in three species of annelid worms. This is valuable as this clade is understudied in the epigenomics field, and annelids represent a huge variety of animals. The use of genomics and functional studies in this work elevates it to one that is relevant to epigeneticists, evolutionary genomicists, and developmental biologists. An important aspect of this study is that it addresses a dogma in the field: that there is a direct correlation between gene body methylation and expression in invertebrates (and vertebrates for that matter). While this work makes some novel findings about this correlation (or lack thereof), more detailed analysis is required to solidify this important advance.

Using a range of methods to assess cytosine methylation, the authors find that one species of annelids has virtually no DNA methylation. In contrast, two others have a low level of DNA methylation that is distributed across the genome in what is considered a stereotypical pattern for many invertebrates: concentrated on a subset of gene bodies and introns, and absent from promoters and transposons. They investigate this pattern across developmental stages and in adult animals, including senescent females, which is aimed at determining whether DNA methylation patterns show an age-associated randomization as found in other species (mostly vertebrates). While interesting, this age related part of the study is the least fleshed out and could be minimized.

*RESPONSE: Following the reviewer’s suggestion, we have shortened this section but maintained these findings as a main figure. We would like to highlight that this observation is quite novel in the field. We are still far from developing methylation clocks for any invertebrate, but the construction of those mathematical models has only been allowed by microarrays in mammals, which make 5mC detection cheaper and scaling up the sample size needed for clocks feasible. Still, finding that global methylation loss correlates with age in invertebrates indicates this is probably achievable.*

Previous studies have suggested that the function of DNA methylation in species with low methylation (like two of the species studied here, O. fusiformis and C. teleta) is to maintain the expression of housekeeping and/or highly expressed genes. The authors examine this and find little to no support for this hypothesis in these animals. Finally, to functionally test the requirement for DNA methylation in O. fusiformis by treating larvae with DNA methyltransferase inhibitors, revealing some defects in development and changes in gene and TE expression, although they find that the different DNMT inhibitors have different levels of efficacy in this experiment, which is curious and not explained.

*RESPONSE: We agree that it is curious that not all DNMT inhibitors have an effect on this species. Despite all the tested cytidine analogues working similarly, for unknown reasons, some work better in some species than others. We have found similar cases of this in unicellular eukaryotes (e.g., DOI: https://doi.org/10.1101/2024.01.08.574619), where only 5-azacytidine produced global methylation loss, whereas zebularine and decitabine did not. This is probably due to the different permeability of the drugs, stability in seawater, incorporation rates, or capacity to trap and degrade various DNMTs. Yet, these options remain untested in this work. In the main text, we highlight this disparity and recommend directly assessing the methylation effects of treatments with cytidine analogues with a quantitative readout (such as sequencing), as many of the previous reports assume cytidine analogues will work in any system, but we show that this is not the case. We discuss this in the revised version of the manuscript (lines 546–578).*

Overall, this is an interesting study that is both broad and deep. The data is presented clearly, and the manuscript is well-written. There are several features of this work that are interesting and novel, including the comparison of three species in the same clade with divergent DNA methylation patterns, the convincing evidence that gene body methylation is not a likely mechanism regulating gene expression, and the intriguing idea that DNA methylation changes during aging in at least one species. Additionally, the inclusion of experimental data is a strength of this study. While these are all positive aspects of this study, there are a number of areas that would benefit from attention, including some controls for the experimental data, a tissue-specific analysis of the methylation and gene expression patterns, and expanding on some of the details, which can involve bringing some of the materials from the supplement into the main figures.

Major Issues:

1. The major issue that would benefit this paper is using tissue-specific methylome/transcriptome to address the correlation between gene body methylation and gene expression in these animals. All of the studies are performed with cells from a wide range of lineages, making it nearly impossible to draw correlations between how and where a gene is expressed and the state of its methylation. Unless they show that there is a static methylation pattern in all cells, as has been reported in vertebrates, one of the main points of this paper is supported by data that is not clear.

*RESPONSE: These animals' small size and anatomy make tissue-specific dissections remarkably difficult, if impossible. In a previous work (DOI: https://doi.org/10.1038/s41586-022-05636-7), we isolated body parts and organs from O. fusiformis, which are not tissue-specific and thus have cells from drastically different embryonic lineages and would thus make a methylome of a “head” or “trunk” as heterogeneous as the whole-body samples reported in the manuscript. However, to address the reviewer’s point, we have instead generated deep-coverage EM-seq methylomes of oocytes and sperm of O. fusiformis. These are single, homogeneous cell types and thus overcome the challenges of adult somatic tissue dissections. The methylation levels in these gametes are comparable (slightly lower, yet this might be due to EM-seq vs WGBS slight coverage biases) to those in gastrula and larval stages but higher than in adults, suggesting that in the germline there is less methylation loss than in the somatic tissues or that there is a re-methylation process in the germ line. This is currently hard to discriminate, as dissecting the germline is not feasible, and we lack a proper understanding of germline formation in the studied annelids. However, the correlation between gene body methylation and transcriptional levels/stability also applies to oocytes as it does in developmental samples, strengthening our previous observations. These data are now in Supplementary Figure 6d–f and the main text (lines 225–227, 246–249, and 487–490).*

2. The intermediate methylation data can confound the correlation studies. If only the high (green bars in Figure 2A-B) and low/no methylation (orange and grey bars in Figure 2A-B) are separated, what is the correlation with expression? This is addressed only partially in Figure 2C-D, and it looks like only those with very high methylation are highly expressed; it would be interesting to look at this in more detail to see what genes correlate with this pattern and what do not.

*RESPONSE: Following the reviewer’s suggestion, we have plotted the transcriptional levels of genes in the gastrula and adult stages of O. fusiformis and C. teleta based on two broad methylation states (low: 0 = mCG < 0.2; high: mCG > 0.8; Supplementary Figure S8). We found that highly methylated genes tend to have higher expression levels than lowly methylated genes. We have further explored the genes that do not fit this pattern in the gastrula stage of O. fusiformis and C. teleta, e.g. highly expressed unmethylated genes or lowly expressed methylated genes. These genes suggest that transcriptional stability might underpin the cases where methylation levels do not positively correlate with expression levels, as many developmental genes with variable expression might remain unmethylated despite having high expression at a given time point (lines 238–242).*

3. One of the most important findings in the paper is the functional validation of the importance of DNA methylation in annelid development. In Fig. 5D, zebularine treatment of O. fusiformis results in the expression of TEs from intergenic regions, but the analysis of DNA methylation values on TEs after treatment are missing and should be included.

*RESPONSE: We have now sequenced the control DMSO and zebularine-treated EM-seq libraries deeper and provided the methylation levels on differentially expressed TEs and genes in new Supplementary Figure S19. Methylation levels on both TEs and genes appear slightly lower in zebularine-treated samples (see quartile ranges) yet still largely overlap. This is, however, not surprising, given the modest global difference of ~4%.*

4. Figure 1 would benefit from presenting data that gives an overview of these species. Figure 1b could be adjusted to not only include TE abundance in these genomes but could also include the other genomic elements (promoters, exons, introns, etc.) to give a sense of how these genomes are organized.

*RESPONSE: Figure 1b now includes the fraction of the genome corresponding to protein-coding exons and a more detailed annotation of TEs (see point below). In addition, we have made the new panels c, d, and e that expand the comparison between the genomes of these three annelids for gene and intron numbers, as well as CDS and intron lengths.*

5. Similarly, an overview of where 5mC is distributed in each genome would be of interest -i.e. by genomic element, chromosome etc.

*RESPONSE: We now include this in the new Supplementary Figure 6a–c, which complements Supplementary Figure 15 (5mC in TEs). We characterised the distribution of methylation states for each chromosome in O. fusiformis (the only annelid of our study with a chromosome-level genome assembly) and different features of O. fusiformis’ and C. teleta’s genome (genes, intergenic regions, promoters, CDS, and introns). These analyses support the gene body methylation and higher levels of 5mC in the introns of O. fusiformis, likely caused by the higher number of intronic TEs in this species (Supplementary Figure 15). We included these new findings in the main text (lines 215–219).*

6. Data in figure 1 and S5 show that there is a spatiotemporal pattern of the DNMTs, MBDs, and TETs during development. The interpretation of this data is cursory. If these genes are patterning the methylome, which is present in all cells, then how is it that not all cells express them? Instead, if they are functioning during only discrete developmental events, then how is the methylome in the rest of the embryo patterned? Finally, Dnmt1 typically shows an expression pattern that mirrors cells in S-phase. Can that explain the discrete pattern of expression seen in late embryos and larvae? Some additional staining with other genes – such as Uhrf1 and Pcna – would help clarify this.

*RESPONSE: We have performed in situ hybridisation and immunolabelling of PCNA in O. fusiformis, demonstrating that PCNA localises to the germinal vesicle in the oocyte, and the expression pattern shows asymmetric localisation in the foregut and ventral region of the early larva and juvenile rudiment of the competent, mirroring what we originally described for DNMT1. However, as EdU and phosphorylated histone 3.3 staining revealed, cycling and dividing cells are widespread in the larva, suggesting that in situ hybridisation might be only detecting those tissues with higher levels and that low background expression is likely common to all cells, as the reviewer pointed out. All these new data are in Supplementary Figure 5 and discussed in more detail, as requested by the reviewer, in lines 178–182.*

7. The data on TEs would benefit from some more detailed investigation. The pie charts in Figure 1 show transposable elements (TEs) but in the caption, they are referred to as repetitive elements. This is an important distinction. It would be nice to have these elements fully annotated and correlated with DNA methylation levels, age, and genomic location.

*RESPONSE: We have updated Figure 1b to include the different classes of TEs and amended the caption accordingly. In addition, the updated Supplementary Figure 15 presents a detailed correlation between DNA methylation and TE elements (by class, age, and genomic location) (see the point below).*

Minor Issues:

1. Uhrf1 is missing from the comparison of conservation of methylation machinery in Figure 1C.

*RESPONSE: Added as suggested. Furthermore, in Supplementary Figure S4, we also show the domain architectures of C. teleta and H. sapiens URHF1 orthologues.*

2. Given that TETs lack the key residues for cytosine demethylation in the species investigated, it is important to comment on whether the TETs in the species studied here are predicted to function in DNA methylation or if they have assumed a different function.

*RESPONSE: Following reviewer #1's point, we have added the domain architecture of the more canonical human TET1 and TET3 and highlighted the position of the catalytic motif HxD, which is present in all annelid TET proteins, including the more divergent TETa of D. gyrociliatus. We mention in the text that the lack of a zfCXXC motif probably decreases the DNA binding specificity of these TETs, as is likely the case in many other invertebrate TET genes (line 159). However, it is known that TETs can act independently of their catalytic activity, as they seem to do in organisms lacking DNA methylation (e.g. Drosophila melanogaster), so it is possible that TETs may play other roles other than 5mC oxidation in annelids.*

3. The data in S5 would be useful to have in the main Figure 1, as it gives a broad overview of what is happening when during development of these animals. The in situs are valuable data.

4. The data in Figure S6 are really nice and would benefit from being in the main figure.

*RESPONSE: We have moved both supplementary figures to the main text and removed the summary panels d, e, and g in Figure 1 accordingly.*

5. One interesting finding in Fig. S4 and S5 is that C. teleta has two DNMT1s, with one missing CXXC domain (DNMT1a) while the other missing RFD domain (DNMT1b). Since Fig. S5 showed that DNMT1b has an expression profile similar to UHRF1, perhaps this is the DNA methylation maintenance enzyme in this organism while DNMT1a may have another role. It seems that C. teleta has split different roles of DNMT1 into two distinct genes. Could the authors comment on this and report if to their knowledge there are other organisms that invoke such strategy in their genomes?

*RESPONSE: We now refer to the cases of DNMT1 duplications in some animal lineages (insects, marsupials, sponges) and the possible sub- or neofunctionalisation of these two copies in C. teleta, as previously proposed for the opossum (lines 450–454).*

6. The phenotypic differences in the embryos treated with zebularine and 5-azacytodine are very clear, but it is not clear if all embryos have this response or if it is only some, and if it is only some, which were sequenced? A simple scoring of phenotypic penetrance would be helpful.

*RESPONSE: The phenotypic penetrance is indicated in Supplementary Figure S18a in the merged panel, and we have added a new supplementary Table S6 with the observed penetrance in three independent replicates. Percentages in the main originally referred to replicate 1 and are now the average of the three replicates. In all cases, embryos were pre-sorted to include mostly those with a phenotype (or wild-type morphology in the control) for downstream analyses.*

7. In Fig 1c, the crossed-circle for ‘zf-CXXC domain loss’ is missing in one of the paralogs of DNMT1 in C. teleta. Also, a ‘visual code’ for missing RFD domain in DNMT1.

8. In Fig.3c-d, the color coding of Hyper-mCG in O. fusiformis in the box plot could be more divergent from the color used for C. teleta to make the graph more readable.

*RESPONSE: These two points were corrected as suggested.*

9. In Fig.S14, it seems that panels are missing about adult or gastrula respectively in each animal. In O. fusiformis, box plots of TEs divided by class are presented only for gastrula and not for the adult, while for C. teleta, only adult panels are reported and gastrula data are missing.

*RESPONSE: We have updated this supplementary figure to include both species' Kimura divergence-based analyses for gastrula and adult stages. We focused on the gastrula to analyse methylation levels according to TE class because global methylation levels are higher at this stage.*

Reviewer #3:

This study is important, as it contributes vital knowledge about the highly evolutionarily dynamic patterns of methylation among animal species. Base-resolution studies such as these are crucial for the field to move forward. There are many nice observations and important results, however there are major flaws that need to be addressed and clarified.

Major concerns:

The functional analysis is of most concern, as only base-analogs were used to disrupt methylation, and this approach has many caveats that make results hard to interpret in any model system (see review Seelan et al., 2018 DOI: 10.1080/03602532.2018.1437446

). In the present case only one of the drugs was shown to have a potentially specific effect, as the azacytidine and decitabine appeared to be toxic without affecting DNA methylation. The reason why no changes in DNA methylation were observed despite obvious effects of the drugs is not explored, and is concerning.

*RESPONSE: We have now deep-sequenced the methylomes of control and zebularine-treated embryos to characterise in more detail the changes in DNA methylation between these two conditions. We agree with the reviewer that base analogues are a coarse approach but the most accessible and feasible in non-model species. We now state more obviously the limitations of this approach and refer to the manuscript by Seelan and co-workers, as it matches our observations (i.e., 5-azacytidine has no effect on DNA methylation but alters gene expression) (lines 565–566).*

For the putatively functional zebulanine, the reduction of methylation was apparently quite modest in magnitude. Given that the other drugs give massive morphological phenotypes and presumably major alterations in gene expression patterns, despite no changes in methylation. It is entirely possible that the mild gene expression effects and phenotypes are due simply to a milder toxicity and are not related the minor reduction in methylation levels. The authors do not address this possibility, but it is something that needs to be falsified in order for their claims about the functions of methylation to be credible (especially in the discussion).

*RESPONSE: We now acknowledge the possibility that the gene expression changes and phenotypes after zebularine treatment might be due to the drug's potential mild toxicity and not to the otherwise significant hypomethylation caused by this treatment (lines 565–566). We also discuss the possibility of 5mC independent effects of zebularine on gene expression. Given the modest demethylation, it is hard to tease apart which events are primarily caused by de-methylation and which are secondary effects. Yet, finding that most TEs are originally methylated and upregulated points towards some 5mC-associated roles (see lines 546–578).*

Missing from the methods is how many replicates were done for both the EM-seq and RNA-seq after drug treatment, this can also affect the credibility of the results and should be included.

*RESPONSE: We have now added this information (lines 744–746); this was done in duplicates.*

Finally, it appears that CpG conversion in lambda DNA was noticeably less efficient in the zebulanine experiment. The exact magnitude is not clear by eye in the figure, but the authors should give an indication of the expected increase false negative methylation calls in the whole genome sample based on the rate in the phage DNA. Would correcting for this evaporate the statistical significance of the modest methylation reduction observed.

*RESPONSE: We have added Supplementary Table S7 with the exact methylation and non-conversion rate values and updated the figure panel with the corrected (non-conversion rate subtracted) methylation values. After rerunning the statistical tests, the modest decrease in methylation levels is still significant. When sequencing these samples at deeper coverage for DMR analysis, we again found that the global methylation level is highly reproducible across duplicates (32.6% and 32.7% DMSO, 28.4% and 28.5% Zebularine), and the treatment difference is 4.2% mCG, whereas the lambda non-conversion rate difference varies from 0.3 to 0.5%, therefore making non-conversion issues an unlikely confounder of global methylation loss. Similarly, the pUC19 methylated control exhibited a constant 97% mCG in all samples.*

One possibility to partially address these concerns could be to correlate genes with changed expression levels, with observed changes in methylation. It seems that they did genome wide methylation sequencing for the drug treated samples, so this should be possible. If methylation was important, there should be some corrlelation of changes in gene expression, and changes in methylation at the particular locus.

*RESPONSE: In the original manuscript, we did not have deep sequencing for these treated samples, but now, with deeper sequencing, we can address this question. We have not detected statistically significant DMRs between treatment and DMSO control (dmrseq FDR <0.05), but this is expected since cytidine analogue treatments block DNMTs and are likely to lead to stochastic methylation loss across the genome, not in a region-specific manner. Also, given a global loss of 4.2% mCG, the signal for some regions losing more methylation than the rest is hard to detect. Most are hypomethylated when analysing the methylation levels on genes or TEs that are dysregulated upon zebularine treatment. However, there are plenty of genes that show similar demethylation levels, but they are not among those detected as differentially expressed between treatment and control. This is expected, as demethylation by cytidine analogues in vertebrates does not lead to up-regulation of every single gene but just a subset of methylation-sensitive genes. Triple DNMT-knock-out mouse embryonic stem cell lines also show few differentially expressed genes compared to wild-type cells, which again implies that 5mC can be compensated by other mechanisms and only some loci are sensitive to 5mC loss. In our experiment, we have a lower penetrance of 5mC loss combined with the cell type heterogeneity of the larva. Thus, we probably only detect the genes/TEs with higher methylation sensitivity. There may still be 5mC-indirect effects on transcription, which we acknowledge in the text (lines 546–578).*

Similarly, the results with TE's after drug treatment needs more support. Activation of TE's is common result of stress, so it would not be suprising to see them activated after a toxic treatment. Consistent with TE activation not being related to changes in methylation, the authors state that the upregulated TEs are almost exclusively intergenic. However, earlier they show that it is the intragenic TE's that are associated with high levels of methylation. One would therefore expect the intragenic TE's to be more susceptible to changes in methylation, and explanation is needed why this is not the case.

*RESPONSE: Although TEs in introns reside in regions generally more methylated (gene body methylation), not all will have the same status of conservation or methyl-sensitivity. Also, host-gene transcriptional read-through or their presence within a gene might imply other regulation mechanisms (e.g. H3K36me3), which might impede their upregulation by inhibiting intra-genic transcriptional start sites. We observe similar patterns of methylation loss in intergenic and intra-genic TEs, with similar rates of 5mC loss in each (Supplementary figure S19). As an analogy, not all LINE elements are active in the human genome, and not all those that are unmethylated are transcribed (see PMID: 38309261). The link between 5mC and silencing is complex and still debated in well-established models, including human cell lines, where demethylation is usually associated with transcription but is not the only cause. Therefore, it is hard to dissect what determines methyl sensitivity in a new system with limited tools, such as cytidine analogues in Owenia. We acknowledge this and discuss avenues to improve these observations in the future (lines 546–578).*

Minor points:

The acronym EM-seq should be defined on first use It would be worthwhile to discuss the advantages/disadvantages/interchangeability of this method vs WGBS.

In the figure legend, figure 1f is described out of order, and should follow the alphabetic order in the figure.

*RESPONSE: Both points changed as suggested. We added a sentence highlighting the advantages of EM-seq over WGBS, which particularly overcomes some of the limitations of C. teleta for DNA methylation analyses (lines 652–654).*

**Second round of review**

**Reviewer 1**

All my concerns have been addressed satisfactorily.

**Reviewer 2**

In this revised version, the authors addressed all the concerns raised in the previous review. The newly configured figures along with the new data improved what was already a strong and interesting study. There are few minor points that remain to be fixed:

- In Fig.S4c, the structural domain of UHRF1 protein are reported only for C. teleta, while all the three annelids have a conserved sequence according to main Fig.1f. In addition, the legend naming/color and position in the protein of the domains are incorrect (PHD/SRA swapped and RING missing).

- In Fig.S6a-d, the y-axis label report 0-100 range of 5mC methylation but the data represent a proportional stack bar of binned methylation values by high, intermediate, low and no 5mC methylation values. This is confusing.

**Reviewer 3**

The authors have addressed my concerns by either performing additional experiments or softening their claims. I have no more major concerns.

**Authors Response**

**Point-by-point responses to the reviewers’ comments:**

Reviewer #1: All my concerns have been addressed satisfactorily.

Reviewer #2: In this revised version, the authors addressed all the concerns raised in the previous review. The newly configured figures along with the new data improved what was already a strong and interesting study. There are few minor points that remain to be fixed:

- In Fig.S4c, the structural domain of UHRF1 protein are reported only for C. teleta, while all the three annelids have a conserved sequence according to main Fig.1f. In addition, the legend naming/color and position in the protein of the domains are incorrect (PHD/SRA swapped and RING missing).

- In Fig.S6a-d, the y-axis label report 0-100 range of 5mC methylation but the data represent a proportional stack bar of binned methylation values by high, intermediate, low and no 5mC methylation values. This is confusing.

*RESPONSE: These two points have been modified as the reviewer indicated.*

Reviewer #3: The authors have addressed my concerns by either performing additional experiments or softening their claims. I have no more major concerns.
